# Novel loci for childhood body mass index and shared heritability with adult cardiometabolic traits

**DOI:** 10.1371/journal.pgen.1008718

**Published:** 2020-10-12

**Authors:** Suzanne Vogelezang, Jonathan P. Bradfield, Tarunveer S. Ahluwalia, John A. Curtin, Timo A. Lakka, Niels Grarup, Markus Scholz, Peter J. van der Most, Claire Monnereau, Evie Stergiakouli, Anni Heiskala, Momoko Horikoshi, Iryna O. Fedko, Natalia Vilor-Tejedor, Diana L. Cousminer, Marie Standl, Carol A. Wang, Jorma Viikari, Frank Geller, Carmen Íñiguez, Niina Pitkänen, Alessandra Chesi, Jonas Bacelis, Loic Yengo, Maties Torrent, Ioanna Ntalla, Øyvind Helgeland, Saskia Selzam, Judith M. Vonk, Mohammed H. Zafarmand, Barbara Heude, Ismaa Sadaf Farooqi, Akram Alyass, Robin N. Beaumont, Christian T. Have, Peter Rzehak, Jose Ramon Bilbao, Theresia M. Schnurr, Inês Barroso, Klaus Bønnelykke, Lawrence J. Beilin, Lisbeth Carstensen, Marie-Aline Charles, Bo Chawes, Karine Clément, Ricardo Closa-Monasterolo, Adnan Custovic, Johan G. Eriksson, Joaquin Escribano, Maria Groen-Blokhuis, Veit Grote, Dariusz Gruszfeld, Hakon Hakonarson, Torben Hansen, Andrew T. Hattersley, Mette Hollensted, Jouke-Jan Hottenga, Elina Hyppönen, Stefan Johansson, Raimo Joro, Mika Kähönen, Ville Karhunen, Wieland Kiess, Bridget A. Knight, Berthold Koletzko, Andreas Kühnapfel, Kathrin Landgraf, Jean-Paul Langhendries, Terho Lehtimäki, Jaakko T. Leinonen, Aihuali Li, Virpi Lindi, Estelle Lowry, Mariona Bustamante, Carolina Medina-Gomez, Mads Melbye, Kim F. Michaelsen, Camilla S. Morgen, Trevor A. Mori, Tenna R. H. Nielsen, Harri Niinikoski, Albertine J. Oldehinkel, Katja Pahkala, Kalliope Panoutsopoulou, Oluf Pedersen, Craig E. Pennell, Christine Power, Sijmen A. Reijneveld, Fernando Rivadeneira, Angela Simpson, Peter D. Sly, Jakob Stokholm, Kook K. Teo, Elisabeth Thiering, Nicholas J. Timpson, André G. Uitterlinden, Catharina E. M. van Beijsterveldt, Barbera D. C. van Schaik, Marc Vaudel, Elvira Verduci, Rebecca K. Vinding, Mandy Vogel, Eleftheria Zeggini, Sylvain Sebert, Mads V. Lind, Christopher D. Brown, Loreto Santa-Marina, Eva Reischl, Christine Frithioff-Bøjsøe, David Meyre, Eleanor Wheeler, Ken Ong, Ellen A. Nohr, Tanja G. M. Vrijkotte, Gerard H. Koppelman, Robert Plomin, Pål R. Njølstad, George D. Dedoussis, Philippe Froguel, Thorkild I. A. Sørensen, Bo Jacobsson, Rachel M. Freathy, Babette S. Zemel, Olli Raitakari, Martine Vrijheid, Bjarke Feenstra, Leo-Pekka Lyytikäinen, Harold Snieder, Holger Kirsten, Patrick G. Holt, Joachim Heinrich, Elisabeth Widén, Jordi Sunyer, Dorret I. Boomsma, Marjo-Riitta Järvelin, Antje Körner, George Davey Smith, Jens-Christian Holm, Mustafa Atalay, Clare Murray, Hans Bisgaard, Mark I. McCarthy, Early Growth Genetics Consortium, Vincent W. V. Jaddoe, Struan F. A. Grant, Janine F. Felix

**Affiliations:** 1 The Generation R Study Group, Erasmus MC, University Medical Center, Rotterdam, the Netherlands; 2 Department of Pediatrics, Erasmus MC, University Medical Center, Rotterdam, the Netherlands; 3 Department of Epidemiology, Erasmus University Medical Center, Rotterdam, the Netherlands; 4 Quantinuum Research LLC, San Diego, California, United States of America; 5 Center for Applied Genomics, Division of Human Genetics, Children’s Hospital of Philadelphia, Philadelphia, Pennsylvania, United States of America; 6 Copenhagen Prospective Studies on Asthma in Childhood, Herlev and Gentofte Hospital, University of Copenhagen, Copenhagen, Denmark; 7 Steno Diabetes Center Copenhagen, Gentofte, Denmark; 8 Novo Nordisk Foundation Center for Basic Metabolic Research, Faculty of Health and Medical Sciences, University of Copenhagen, Copenhagen, Denmark; 9 The Bioinformatics Center, Department of Biology, University of Copenhagen, Copenhagen, Denmark; 10 Division of Infection Immunity and Respiratory Medicine, School of Biological Sciences, The University of Manchester, Manchester Academic Health Science Centre, and Manchester University NHS Foundation Trust, Manchester, United Kingdom; 11 Institute of Biomedicine, Physiology, University of Eastern Finland, Kuopio, Finland; 12 Foundation for Research in Health Exercise and Nutrition, Kuopio Research Institute of Exercise Medicine, Kuopio, Finland; 13 Department of Clinical Physiology and Nuclear Medicine, Kuopio University Hospital, Kuopio, Finland; 14 Institute for Medical Informatics, Statistics and Epidemiology, University of Leipzig, Leipzig, Germany; 15 LIFE Research Center for Civilization Diseases, University of Leipzig, Leipzig, Germany; 16 Department of Epidemiology, University of Groningen, University Medical Center Groningen, Groningen, the Netherlands; 17 MRC Integrative Epidemiology Unit at the University of Bristol, Bristol, United Kingdom; 18 Population Health Sciences, Bristol Medical School, University of Bristol, Bristol, United Kingdom; 19 School of Oral and Dental Sciences, University of Bristol, Bristol, United Kingdom; 20 Center for Life Course Health Research, University of Oulu, Oulu, Finland; 21 Wellcome Centre for Human Genetics, University of Oxford, Oxford, United Kingdom; 22 Oxford Centre for Diabetes, Endocrinology and Metabolism, University of Oxford, Oxford, United Kingdom; 23 RIKEN Center for Integrative Medical Sciences, Yokohama, Kanagawa, Japan; 24 Department of Biological Psychology, Vrije Universiteit Amsterdam, Amsterdam, the Netherlands; 25 ISGlobal, Barcelona, Spain; 26 Centre for Genomic Regulation (CRG), The Barcelona Institute for Science and Technology, Barcelona, Spain; 27 BarcelonaBeta Brain Research Center (BBRC), Pasqual Maragall Foundation, Barcelona, Spain; 28 CIBER Epidemiología y Salud Pública (CIBERESP), Madrid, Spain; 29 Division of Human Genetics, Children’s Hospital of Philadelphia, Philadelphia, Pennsylvania, United States of America; 30 Department of Genetics, University of Pennsylvania, Philadelphia, Pennsylvania, United States of America; 31 Institute of Epidemiology, Helmholtz Zentrum München- German Research Center for Environmental Health, Neuherberg, Germany; 32 School of Medicine and Public Health, Faculty of Medicine and Health, The University of Newcastle, Newcastle, Australia; 33 Department of Medicine, University of Turku, Turku, Finland; 34 Division of Medicine, Turku University Hospital, Turku, Finland; 35 Department of Epidemiology Research, Statens Serum Institut, Copenhagen, Denmark; 36 Department of Statistics and Computational Research–Universitat de València, València, Spain; 37 Epidemiology and Environmental Health Joint Research Unit, FISABIO-Universitat Jaume I-Universitat de València, València, Spain; 38 Research Centre of Applied and Preventive Cardiovascular Medicine, University of Turku, Turku, Finland; 39 Centre for Population Health Research, University of Turku and Turku University Hospital, Turku, Finland; 40 Department of Obstetrics and Gynecology, Institute of Clinical Science, Sahlgrenska Academy, University of Gothenburg, Gothenburg Sweden; 41 Region Västra Götaland, Sahlgrenska University Hospital, Department of Obstetrics and Gynecology, Gothenburg Sweden; 42 University Lille, Centre National de la Recherche Scientifique, Institut Pasteur de Lille, UMR 8199—European Genomic Institute for Diabetes, Lille, France; 43 Institute for Molecular Bioscience, The University of Queensland, Brisbane, Australia; 44 Area de Salut de Menorca ib-salut, Menorca, Spain; 45 Institut d'Investigacio Sanitaria Illes Balears (IdISBa), Palma de Mallorca, Spain; 46 William Harvey Research Institute, Barts and the London School of Medicine and Dentistry, Queen Mary University of London, London, United Kingdom; 47 KG Jebsen Center for Diabetes Research, Department of Clinical Science, University of Bergen, Bergen, Norway; 48 Department of Genetics and Bioinformatics, Health Data and Digitalization, Norwegian Institute of Public Health, Oslo, Norway; 49 Social, Genetic and Developmental Psychiatry Centre, Institute of Psychiatry, Psychology and Neuroscience, King’s College London, London, United Kingdom; 50 Department of Epidemiology, GRIAC (Groningen Research Institute for Asthma and COPD), University of Groningen, University Medical Center Groningen, Groningen, the Netherlands; 51 Department of Public Health, Amsterdam Public Health Research Institute, Amsterdam UMC, University of Amsterdam, Amsterdam, the Netherlands; 52 Department of Obstetrics & Gynecology, Amsterdam UMC, University of Amsterdam, Amsterdam, the Netherlands; 53 Department of Clinical Epidemiology, Biostatistics and Bioinformatics, Amsterdam Public Health Research Institute, Amsterdam UMC, University of Amsterdam, Amsterdam, the Netherlands; 54 Université de Paris, CRESS, INSERM, INRA, Paris, France; 55 University of Cambridge Metabolic Research Laboratories and NIHR Cambridge Biomedical Research Centre, Wellcome Trust-MRC Institute of Metabolic Science, Addenbrooke’s Hospital, Cambridge, United Kingdom; 56 Department of Health Research Methods, Evidence, and Impact, McMaster University, Hamilton, Canada; 57 Institute of Biomedical and Clinical Science, College of Medicine and Health, University of Exeter, Exeter, United Kingdom; 58 Division of Metabolic and Nutritional Medicine, Dr. von Hauner Children's Hospital, Ludwig-Maximilians Universität München (LMU), Munich, Germany; 59 University of the Basque Country (UPV/EHU), Leioa, Spain; 60 Biocrues-Bizkaia Health Research Institute, Barakaldo, Spain; 61 CIBER Diabetes y Enfermedades Metabólicas (CIBERDEM), Spain; 62 Wellcome Sanger Institute, Cambridge, United Kingdom; 63 MRC Epidemiology Unit, University of Cambridge, Cambridge, United Kingdom; 64 Medical School, The University of Western Australia, Perth, Western Australia, Australia; 65 Nutrition and Obesities; systemic approaches research unit, Sorbonne University, INSERM, Pitie- Salpêtrière Hospital, Assistance Publique hôpital de Paris, Paris, France; 66 Pediatrics, Nutrition and Development Research Unit, Universitat Rovira i Virgili, IISPV, Reus, Spain; 67 National Heart and Lung Institute, Imperial College London, London, United Kingdom; 68 Department of General Practice and Primary Health Care, University of Helsinki and Helsinki University Hospital, Helsinki, Finland; 69 Department of Obstetrics & Gynecology, Yong Loo Lin School of Medicine, National University of Singapore, Singapore; 70 Neonatal Department, Children's Memorial Health Institute, Warsaw, Poland; 71 Department of Pediatrics, Perelman School of Medicine, University of Pennsylvania, Philadelphia, United States of America; 72 NIHR Exeter Clinical Research Facility, College of Medicine and Health, University of Exeter, and Royal Devon and Exeter NHS Foundation Trust, Exeter, United Kingdom; 73 The Danish Diabetes Academy, Odense, Denmark; 74 Australian Centre for Precision Health, University of South Australia Cancer Research Institute, Adelaide, Australia; 75 Population, Policy and Practice, UCL Institute of Child Health, University College London, London, United Kingdom; 76 South Australian Health and Medical Research Institute, Adelaide, Australia; 77 Department of Medical Genetics, Haukeland University Hospital, Bergen, Norway; 78 Department of Clinical Physiology, Tampere University Hospital, Tampere, Finland; 79 Department of Clinical Physiology, Finnish Cardiovascular Research Center—Tampere, Faculty of Medicine and Health Technology, Tampere University, Tampere, Finland; 80 Department of Epidemiology and Biostatistics, School of Public Health, Imperial College, London, United Kingdom; 81 MRC-PHE Centre for Environment and Health, School of Public Health, Imperial College, London, United Kingdom; 82 Center for Pediatric Research, University Hospital for Children and Adolescents, University of Leipzig, Leipzig, Germany; 83 Integrated Research and Treatment Center (IFB) Adiposity Diseases, University of Leipzig, Leipzig, Germany; 84 CHC–Health Group, Liège, Belgium; 85 Department of Clinical Chemistry, Fimlab Laboratories, Tampere, Finland; 86 Department of Clinical Chemistry, Finnish Cardiovascular Research Center—Tampere, Faculty of Medicine and Health Technology, Tampere University, Tampere, Finland; 87 Institute For Molecular Medicine Finland, FIMM, University of Helsinki, Helsinki, Finland; 88 University of Eastern Finland Library Kuopio, Kuopio, Finland; 89 Biocenter Oulu, Oulu University Hospital, Oulu, Finland; 90 Universitat Pompeu Fabra (UPF), Barcelona, Spain; 91 Department of Internal Medicine, Erasmus MC, University Medical Center, Rotterdam, the Netherlands; 92 Department of Medicine, Stanford School of Medicine, Stanford, California, United States of America; 93 Department of Nutrition, Exercise and Sports, Faculty of Science, University of Copenhagen, Copenhagen, Denmark; 94 Department of Public Health, Section of Epidemiology, University of Copenhagen, Copenhagen, Denmark; 95 National Insitute of Public Health, University of Southern Denmark, Copenhagen, Denmark; 96 Department of Pediatrics, Hvidovre Hospital, Hvidovre, Denmark; 97 The Children’s Obesity Clinic, Department of Pediatrics, Copenhagen University Hospital Holbæk, Holbæk, Denmark; 98 Department of Physiology, University of Turku, Turku, Finland; 99 Department of Pediatrics, University of Turku, Turku, Finland; 100 Interdisciplinary Center Psychopathology and Emotion Regulation, University of Groningen, University Medical Center, Groningen, the Netherlands; 101 Paavo Nurmi Centre, Sports and Exercise Medicine Unit, Department of Physical Activity and Health, University of Turku, Turku, Finland; 102 Wellcome Trust Sanger Institute, Wellcome Genome Campus, Hinxton, Cambridgeshire, United Kingdom; 103 Population, Policy and Practice, UCL Great Ormond Street Institute of Child Health, University College London, London, United Kingdom; 104 Department of Health Sciences, University of Groningen, University Medical Center Groningen, Groningen, the Netherlands; 105 Child Health Research Centre, University of Queensland, Brisbane, Australia; 106 World Health Organization, WHO Collaborating Centre for Children’s Health and Environment, Brisbane, Queensland, Australia; 107 Department of Medicine, McMaster University, Hamilton, Canada; Department of Health Research Methods, Evidence, and Impact, McMaster University, Hamilton, Canada; 108 Netherlands Genomics Initiative (NGI)-sponsored Netherlands Consortium for Healthy Aging NCHA), Leiden, the Netherlands; 109 Bioinformatics Laboratory, Department of Clinical Epidemiology, Biostatistics and Bioinformatics, Amsterdam Public Health Research Institute, Amsterdam UMC, University of Amsterdam, Amsterdam, the Netherlands; 110 Department of Pediatrics, San Paolo Hospital, University of Milan, Milan, Italy; 111 Institute of Translational Genomics, Helmholtz Zentrum München–German Research Center for Environmental Health, Neuherberg, Germany; 112 TUM School of Medicine, Technical University of Munich and Klinikum Rechts der Isar, Munich, Germany; 113 Section of Genomics of Common Disease, Department of Medicine, Imperial College London, London, United Kingdom; 114 Department of Genetics, University of Pennsylvania, Perelman School of Medicine, Philadelphia, Pennsylvania, United States of America; 115 Consortium for Biomedical Research in Epidemiology and Public Health (CIBER en Epidemiologia y Salud Publica-CIBERESP), Barcelona, Spain; 116 Biodonostia Health Research Institute, San Sebastian, Spain; 117 Subdirección Salud Pública de Gipuzkoa, San Sebastian, Spain; 118 Research Unit of Molecular Epidemiology, Institute of Epidemiology, Helmholtz Zentrum Muenchen, Munich, Germany; 119 University of Copenhagen, Faculty of Health and Medical Sciences, Copenhagen N, Denmark; 120 Medical Research Council Epidemiology Unit & Department of Paediatrics, University of Cambridge, Addenbrooke’s Hospital, Cambridge, England; 121 Research Unit for Gynaecology and Obstetrics, Institute of Clinical Research, University of Southern Denmark, Odense, Denmark; 122 University Medical Center Groningen, University of Groningen, Department of Pediatric Pulmonology and Pediatric Allergology, Beatrix Children's Hospital, GRIAC (Groningen Research Institute for Asthma and COPD), Groningen, the Netherlands; 123 Department of Pediatrics and Adolescents, Haukeland University Hospital, Bergen, Norway; 124 Broad Institute of Harvard and MIT, Cambridge, Massachusetts, United States of America; 125 Department of Nutrition and Dietetics, School of Health Science and Education, Harokopio University, Athens, Greece; 126 Medical Research Council Integrative Epidemiology Unit, University of Bristol, Bristol, United Kingdom; 127 Division of Gastroenterology, Hepatology and Nutrition, The Children’s Hospital of Philadelphia, Philadelphia, Pennsylvania, United States of America; 128 Department of Clinical Physiology and Nuclear Medicine, Turku University Hospital, Turku, Finland; 129 Department of Cardiology, Heart Center, Tampere University Hospital, Tampere, Finland; 130 Telethon Kids Institute, The University of Western Australia, Perth, Western Australia, Australia; 131 Institute and Outpatient Clinic for Occupational, Social and Environmental Medicine, Inner City Clinic, University Hospital Munich, Ludwig-Maximilians-Universität of Munich, Munich, Germany; 132 Allergy and Lung Health Unit, Melbourne School of Population and Global Health, The University of Melbourne, Melbourne, Australia; 133 Hospital del Mar Medical Research Institute (IMIM), Barcelona, Spain; 134 Amsterdam Public Health research institute and Amsterdam Reproduction & Development research Institute, Amsterdam, the Netherlands; 135 Oxford National Institute for Health Research (NIHR) Biomedical Research Centre, Churchill Hospital, Oxford, United Kingdom; 136 Center for Spatial and Functional Genomics, Children’s Hospital of Philadelphia, Philadelphia, Pennsylvania, United States of America; The University of North Carolina at Chapel Hill, UNITED STATES

## Abstract

The genetic background of childhood body mass index (BMI), and the extent to which the well-known associations of childhood BMI with adult diseases are explained by shared genetic factors, are largely unknown. We performed a genome-wide association study meta-analysis of BMI in 61,111 children aged between 2 and 10 years. Twenty-five independent loci reached genome-wide significance in the combined discovery and replication analyses. Two of these, located near *NEDD4L* and *SLC45A3*, have not previously been reported in relation to either childhood or adult BMI. Positive genetic correlations of childhood BMI with birth weight and adult BMI, waist-to-hip ratio, diastolic blood pressure and type 2 diabetes were detected (R_g_ ranging from 0.11 to 0.76, P-values <0.002). A negative genetic correlation of childhood BMI with age at menarche was observed. Our results suggest that the biological processes underlying childhood BMI largely, but not completely, overlap with those underlying adult BMI. The well-known observational associations of BMI in childhood with cardio-metabolic diseases in adulthood may reflect partial genetic overlap, but in light of previous evidence, it is also likely that they are explained through phenotypic continuity of BMI from childhood into adulthood.

## Author summary

Although twin studies have shown that body mass index (BMI) is highly heritable, many common genetic variants involved in the development of BMI have not yet been identified, especially in children. We studied associations of more than 40 million genetic variants with childhood BMI in 61,111 children aged between 2 and 10 years. We identified 25 genetic variants that were associated with childhood BMI. Two of these have not been implicated for BMI previously, located close to the genes *NEDD4L* and *SLC45A3*. We also show that the genetic background of childhood BMI overlaps with that of birth weight, adult BMI, waist-to-hip-ratio, diastolic blood pressure, type 2 diabetes, and age at menarche. Our results suggest that the biological processes underlying childhood BMI largely overlap with those underlying adult BMI. However, the overlap is not complete. Additionally, the genetic backgrounds of childhood BMI and other cardio-metabolic phenotypes are overlapping. This may mean that the associations of childhood BMI and later cardio-metabolic outcomes are partially explained by shared genetics, but it could also be explained by the strong association of childhood BMI with adult BMI.

## Introduction

Childhood obesity is a major public health problem with impact on health in both the short and the long term [1]. Besides the well-established lifestyle and behavioral factors, genetics influence the risk of obesity, with reported heritability estimates from twin studies for body mass index (BMI) ranging from 40 to 70% [2,3]. An estimated 17 to 27% seems to be explained by common variants [4–6]. Large genome-wide association studies (GWAS) have identified 941 loci associated with adult BMI, accounting for 5% of the phenotypic variation [7]. Less is known about the genetic background of childhood BMI. A previous GWAS of BMI among 35,668 children identified 15 associated loci, accounting for 2% of the phenotypic variance [8]. Of these loci, 12 were also associated with adult BMI [9,10]. The remaining 3 identified genetic loci, specifically associated with childhood BMI, suggest possible age-specific differences between the two stages of life or could indicate stronger effects for these genetic loci in childhood BMI than in adult BMI [11–13]. Thus far, most common variants explaining the genetic variability of childhood BMI remain undetected. It is well known that obesity in early-life tends to track into later life [14]. Furthermore, childhood obesity has been associated with a lower age at menarche and with non-communicable diseases in later life, including hypertension, dyslipidemia, type 2 diabetes, neurodegenerative disease and asthma [15–19]. Findings from recent studies suggest a shared genetic background for BMI in childhood and adulthood [8,20,21]. To which extent the associations of childhood BMI with common adult diseases are genetically explained, has not been explored in detail.

We aimed to study the genetic background of childhood BMI by performing a two-stage GWAS meta-analysis consisting of 41 studies with a total sample size of 61,111 children of European ancestry. We also examined the genetic correlations of childhood BMI with anthropometric, cardio-metabolic, respiratory, neurocognitive and endocrinological traits in adults, using GWAS summary statistics from various consortia.

## Results

### Identification of genome-wide significant loci for childhood BMI

**Fig 1:**
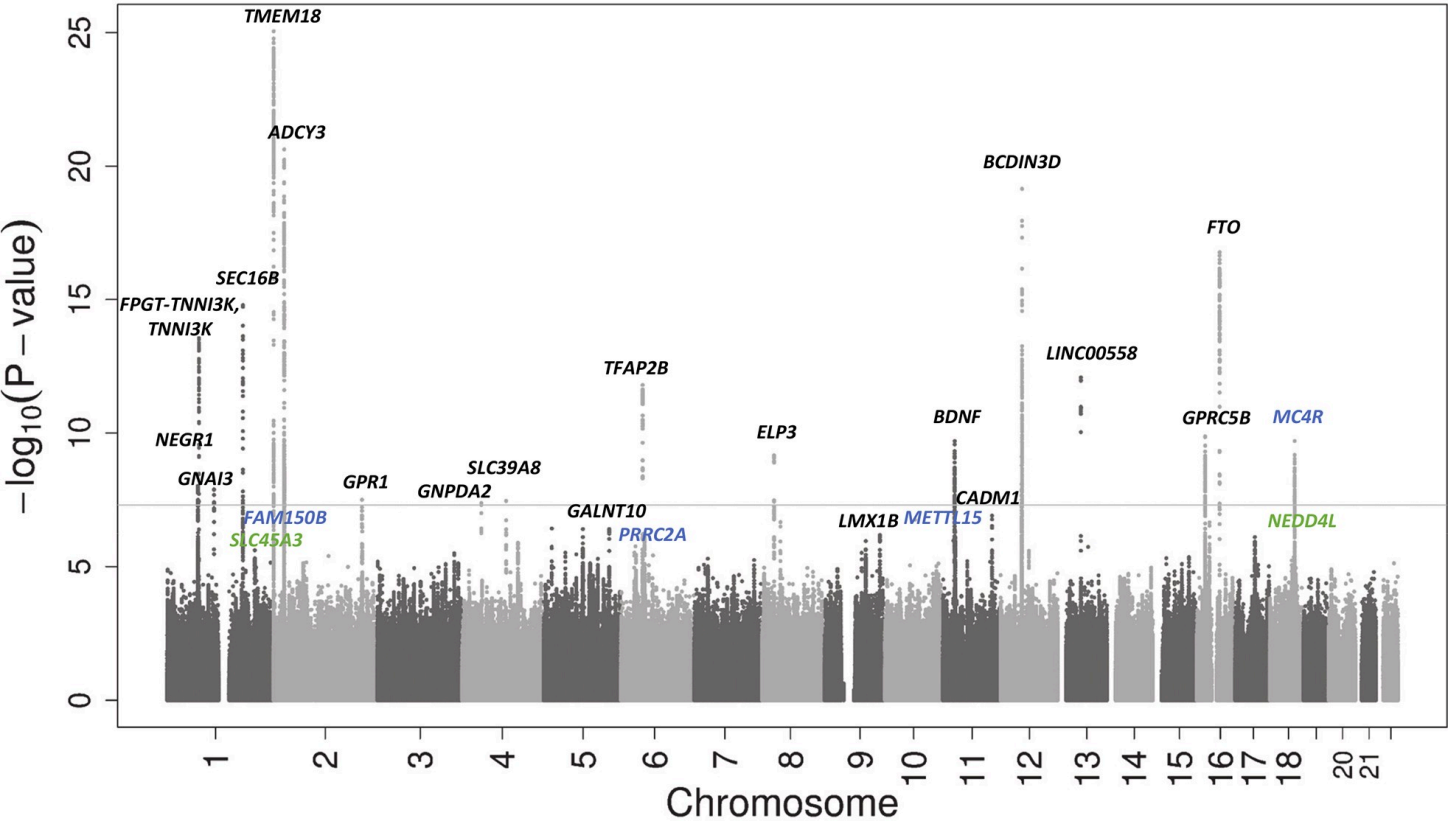
Manhattan plot of results of the discovery meta-analysis of 26 single study GWAS. On the x-axis the chromosomes are shown. On the y-axis the–log 10 of the P-value is shown. Novel SNPs are shown in green. Independent SNPs are shown in blue. Known SNPs are shown in black. The genome wide significance cutoff of 5 × 10^−8^ is represented by the grey dotted line.

Sex- and age-adjusted Standard Deviation Scores (SDS) were created for BMI at the latest time point (oldest age, if multiple measurements were available) between 2 and 10 years using the same software and external reference across all studies (LMS growth; Pan H, Cole TJ, 2012; http://www.healthforallchildren.co.uk). Individual study characteristics are shown in S1 Table. The discovery meta-analysis included data from 26 studies (N_discovery_ = 39,620) with data imputed to the 1000 Genomes Project or The Haplotype Reference Consortium (HRC). We performed a fixed-effects inverse variance-weighted meta-analysis and performed conditional analyses based on summary-level statistics and Linkage Disequilibrium (LD) estimation between SNPs in Genome-wide Complex Trait Analysis (GCTA) to select independently associated SNPs at each locus on the basis of conditional P-values [22]. Seventeen independent SNPs reached genome-wide significance (P-values <5 × 10^−8^) and thirty SNPs showed suggestive association with childhood BMI (P-values >5 × 10^−8^ and <5 × 10^−6^). A Manhattan plot of the discovery meta-analysis is shown in Fig 1. No evidence of inflation due to population stratification or cryptic relatedness or other confounders was observed (genomic inflation factor (lambda) = 1.05; LD-score regression intercept = 1.0) (S1 Fig) [23]. All 47 independent SNPs identified in the discovery meta-analysis were taken forward for analysis in 15 replication cohorts (N_replication_ = 21,491) and results of the two stages were then combined. Results of the discovery, replication and combined meta-analyses are shown in Table 1 and S2 Table and S3 Table. Results of the discovery analysis for SNPs with P-values <5 × 10^−6^ are shown in S4 Table. As the replication stage might lack power to replicate SNPs from the discovery analysis, we consider the joint analysis as the primary analysis.

**Table 1:** Results of the discovery, replication and combined analyses for the 47 loci with P-values <5 x 10^−6^ in the discovery phase.

SNP	CHR	Position	Nearest gene	EA/non_EA	P-value discovery	P-value replication	EAF^a^	Beta combined	SE combined	P-value combined
rs11676272^b^^,^^c^	2	25141538	*ADCY3*	G/A	2.37 x 10^−21^	1.88 x 10^−10^	0.46	0.071	0.006	**3.79 x 10**^**−30**^
rs7138803^b^^,^^c^	12	50247468	*BCDIN3D*	A/G	7.12 x 10^−20^	7.39 x 10^−12^	0.37	0.072	0.006	**4.23 x 10**^**−30**^
rs939584^b^^,^^c^^,^^d^	2	621558	*TMEM18*	T/C	8.85 x10^-26^	2.52 x 10^−6^	0.83	0.092	0.008	**3.73 x 10**^**−29**^
rs17817449^b^^,^^c^	16	53813367	*FTO*	G/T	1.69 x 10^−17^	3.21 x 10^−11^	0.40	0.069	0.006	**2.98 x 10**^**−27**^
rs12042908^b^^,^^c^	1	74997762	*FPGT-TNNI3K*, *TNNI3K*	A/G	2.77 x 10^−14^	2.01 x 10^−12^	0.46	0.064	0.006	**6.37 x 10**^**−25**^
rs543874^b^^,^^c^	1	177889480	*SEC16B*	G/A	1.61 x 10^−15^	6.96 x 10^−8^	0.19	0.075	0.008	**6.02 x 10**^**−22**^
rs56133711^b^	11	27723334	*BDNF*	A/G	2.00 x 10^−10^	3.69 x 10^−6^	0.24	0.056	0.007	**3.75 x 10**^**−15**^
rs2076308^b^^,^^c^	6	50791640	*TFAP2B*	C/G	1.59 x 10^−12^	0.01	0.19	0.058	0.008	**3.07 x 10**^**−13**^
rs4477562^b^^,^^c^^,^^e^	13	54104968	*LINC00558*	T/C	8.29 x 10^−13^	0.01	0.13	0.065	0.009	**5.81 x 10**^**−13**^
rs571312^b^^,^^c^	18	57839769	*MC4R*	A/C	2.00 x 10^−10^	4.84 x 10^−4^	0.23	0.052	0.007	**8.80 x 10**^**−13**^
rs12641981^b^^,^^c^	4	45179883	*GNPDA2*	T/C	4.19 x 10^−8^	7.12 x 10^−6^	0.44	0.045	0.006	**1.29 x 10**^**−12**^
rs62107261^f^	2	422144	*FAM150B*	T/C	3.17 x 10^−7^	6.64 x 10^−6^	0.95	0.121	0.018	**9.93 x 10**^**−12**^
rs114285994^b^	16	19935763	*GPRC5B*	G/A	1.41 x 10^−10^	5.98 x 10^−3^	0.87	0.063	0.009	**1.11 x 10**^**−11**^
rs144376234^b^^,^^c^	1	110114504	*GNAI3*	T/C	1.35 x 10^−8^	2.21 x 10^−3^	0.04	0.111	0.017	**1.38 x 10**^**−10**^
rs1094647	1	205655378	*SLC45A3*	G/A	2.46 x 10^−6^	7.45 x 10^−5^	0.55	0.038	0.006	**7.20 x 10**^**−10**^
rs76227980^f^	18	58036384	*MC4R*	C/T	1.71 x 10^−6^	1.19 x 10^−4^	0.98	0.140	0.023	**8.68 x 10**^**−10**^
rs13107325^b^	4	103188709	*SLC39A8*	T/C	3.51 x 10−^8^	4.84 x 10^−3^	0.07	0.082	0.014	**1.38 x 10**^**−9**^
rs62500888^c^	8	28061823	*ELP3*	A/G	6.91 x 10^−10^	0.09	0.57	0.037	0.006	**1.81 x 10**^**−9**^
rs114670539^c^	2	207064335	*GPR1*	T/C	3.16 x 10^−8^	0.01	0.05	0.088	0.015	**1.92 x 10**^**−9**^
rs61765651^b^	1	72754314	*NEGR1*	C/T	9.50 x 10^−9^	0.03	0.83	0.047	0.008	**4.99 x 10**^**−9**^
rs7719067^b^	5	153538241	*GALNT10*	A/G	3.96 x 10^−7^	4.36 x 10^−3^	0.43	0.036	0.006	**6.54 x 10**^**−9**^
rs11030391^f^	11	28644626	*METTL15*	A/G	4.73 x 10^−7^	6.49 x 10^−3^	0.63	0.036	0.006	**1.51 x 10**^**−8**^
rs184566112	18	55943926	*NEDD4L*	A/T	4.40 x 10^−6^	1.24 x 10^−3^	0.84	0.057	0.011	**4.24 x 10**^**−8**^
rs116664060	6	31592524	*PRRC2A*	C/G	3.03 x 10^−6^	3.28 x 10^−3^	0.18	0.049	0.009	**4.63 x 10**^**−8**^
rs11215427^c^	11	115093438	*CADM1*	G/C	1.25 x 10^−7^	0.05	0.74	0.039	0.007	**4.64 x 10**^**−8**^
rs1336980^c^	9	129377855	*LMX1B*	C/G	6.61 x 10^−7^	0.03	0.36	0.033	0.006	1.17 x 10^−7^
rs146823532^f^	1	74979126	*FPGT-TNNI3K*, *TNNI3K*	A/G	4.09 x 10^−7^	0.03	0.97	0.114	0.022	1.33 x 10^−7^
rs79386556	13	71229046	*LINC00348*	A/G	1.84 x 10^−6^	0.04	0.04	0.095	0.019	4.83 x 10^−7^
rs9942489	6	35323709	*PPARD*	A/T	3.62 x 10^−6^	0.02	0.04	0.081	0.016	5.56 x 10^−7^
rs17086809	9	86708695	*RMI1*	C/T	3.06 x 10^−6^	0.05	0.33	0.038	0.007	9.30 x 10^−7^
rs80332495	5	19191677	*CDH18*	A/G	3.78 x 10^−7^	0.26	0.94	0.090	0.019	1.14 x 10^−6^
rs4594227	15	84497207	*ADAMTSL3*	A/G	4.46 x 10^−6^	0.07	0.56	0.030	0.006	1.75 x 10^−6^
rs2457463	10	70315687	*TET1*	G/T	1.79x 10^−7^	0.43	0.04	0.159	0.034	2.69 x 10^−6^
rs11865086^b^	16	30130493	*MAPK3*	C/A	2.23 x 10^−7^	0.47	0.53	0.029	0.006	3.31 x 10^−6^
rs1565356	6	34046065	*GRM4*	C/A	1.76 x 10^−6^	0.25	0.92	0.057	0.012	4.01 x 10^−6^
rs2952863^b^	4	130759647	*C4orf33*	T/G	3.10 x 10^−6^	0.15	0.30	0.031	0.007	4.27 x 10^−6^
rs2358954	12	66379504	*HMGA2*	T/G	3.07 x 10^−6^	0.17	0.68	0.031	0.007	4.77 x 10^−6^
rs7652876	3	179831733	*PEX5L*	A/C	3.22 x 10^−6^	0.19	0.25	0.033	0.007	6.93 x 10^−6^
rs4923207	11	24757325	*LUZP2*	T/G	1.07 x 10^−6^	0.86	0.81	0.040	0.009	7.05 x 10^−6^
rs7757288	6	55205502	*GFRAL*	G/A	6.64 x 10^−7^	0.47	0.39	0.028	0.006	1.03 x 10^−5^
rs6876477	5	50878621	*ISL1*	A/T	3.03 x 10^−6^	0.35	0.76	0.032	0.007	1.31 x 10^−5^
rs28599560	5	91791853	*FLJ42709*	A/G	3.99 x 10^−7^	0.85	0.61	0.027	0.006	3.48 x 10^−5^
rs117281273	8	42981400	*SGK196*	C/G	2.17 x 10^−7^	0.94	0.97	0.081	0.020	3.48 x 10^−5^
rs9695734	9	96407983	*PHF2*	C/T	1.12 x 10^−6^	0.81	0.84	0.035	0.009	4.99 x 10^−5^
rs72833479	17	45960449	*SP2*	A/G	7.96 x 10^−7^	0.99	0.25	0.029	0.007	6.54 x 10^−5^
rs142367753	2	128938956	*UGGT1*	C/G	4.03 x 10^−6^	0.24	0.98	0.088	0.027	1.34 x 10^−3^
rs6896578	5	76423090	*ZBED3-AS1*	C/T	3.52 x 10^−6^	0.17	0.84	0.025	0.009	4.1 x 10^−3^

CHR, chromosome; EA, effect allele; EAF, effect allele frequency; SE, standard error.

Bolded P-values indicate genome-wide significance in the combined analysis.

Detailed information on beta and SE of the discovery and replication stage separately can be found in S2 Table

a From combined analysis

b Locus previously reported for adult BMI

c Locus previously reported for childhood BMI

d Locus previously reported for adult body fat

e Locus previously reported for childhood obesity

f Independent SNP at the same locus selected by conditional analysis

**Fig 2:**
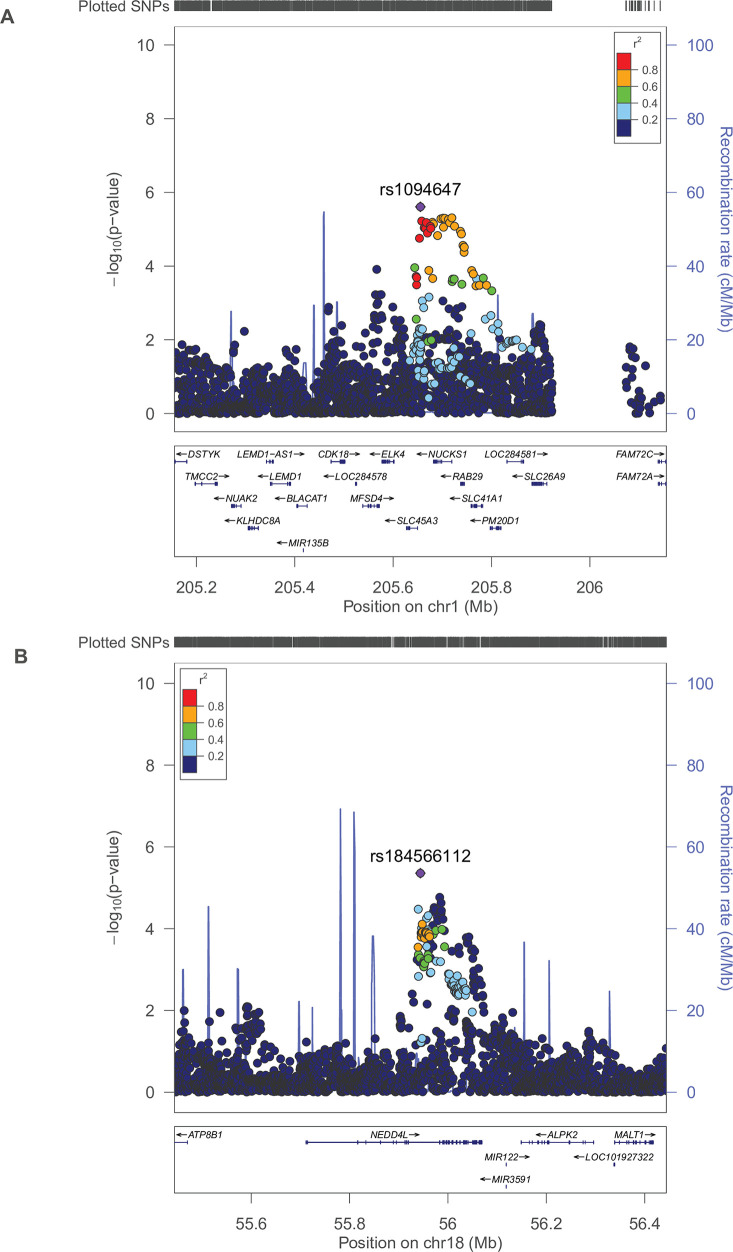
Locus zoom plots of the 2 novel SNPs Regional association plot of the 2 novel SNPs in the 26 childhood BMI discovery studies. SNPs are plotted with their P-values (as–log10; left y-axis) as a function of genomic position (x-axis). Estimated recombination rates (right y-axis) taken from 1000 Genomes, March 2012 release are plotted to reflect the local LD-structure around the top associated SNP (indicated with purple color) and the correlated proxies (indicated in colors). **A.** rs1094647 **B.** rs184566112.

In total, 25 loci achieved genome-wide significance in the combined meta-analysis. We defined a SNP as representing a known BMI-locus if it was within 500 kb of and in LD (r^2^ ≥ 0.2) with a previously reported BMI-associated signal. Of the 25 SNPs, two were novel and had not been previously associated with BMI in either adults or children: rs1094647 near *SLC45A3* and rs184566112 near *NEDD4L*. Per additional risk allele (G, allele frequency = 0.55) of rs1094647 (*SLC45A3*), childhood BMI increased by 0.04 SDS (Standard Error (SE) = 0.01, P-value = 7.20 × 10^−10^), equal to 0.09 kg/m^2^. Per additional risk allele (A, allele frequency = 0.84) of rs184566112 (*NEDD4L*), childhood BMI increased by 0.06 SDS (SE = 0.01; P-value = 4.24 × 10^−8^), equal to 0.11 kg/m^2^. Regional plots of the 2 novel SNPs are shown in Fig 2.

Despite the fact that these novel SNPs were not associated with either childhood or adult BMI previously, they have been reported to be associated with other anthropometric phenotypes. Rs1094647 (*SLC45A3*) has been associated with both height and whole-body fat-free mass in adulthood [24–26]. Additionally, rs708724, which is in high LD with rs1094647 (r^2^ = 0.70) was associated with adult weight [24–26]. Rs184566112 (*NEDD4L*) is located in the same region as rs6567160 (distance = 448 kb, r^2^ <0.2), previously associated with adult body fat [27]. In the current study, we did not observe evidence for association between rs184566112 (*NEDD4L*, effect allele = G, allele frequency = 0.84) and body fat percentage measured by Dual energy X-ray Absorptiometry (age range 24 to 120 months) in 2,698 children from 4 cohorts (0.03 SDS (SE = 0.04, P-value = 0.51)). Individual study characteristics of studies with data on body fat percentage are shown in S5 Table. No evidence of association with childhood obesity was found for the two novel SNPs (P-values >0.11) [28].

We additionally identified 2 independent SNPs (*METTL15* and *PRRC2A*) within 500 kb of previously reported SNPs associated with adult BMI, but only in weak LD with prior reported signals (r^2^ <0.2). Similarly, we found 2 independent SNPs in regions that are known for both childhood and adult BMI (*FAM150B* and *MC4R*) [7,8,10]. Regional plots of the 4 independent SNPs at known loci are shown in S2 Fig. Of the remaining 19 SNPs, 6 mapped to loci previously associated with adult BMI (*BDNF*, *GPRC5B*, *SLC39A8*, *NEGR1*, *GALNT10*, and *CADM1*), 2 mapped to loci previously associated with childhood BMI only (*ELP3* and *GPR1*) and 11 SNPs mapped to loci known to be associated with both adult and childhood BMI (*ADCY3*, *BCDIN3D*, *TMEM18*, *FTO*, *FPGT-TNNI3K/TNNI3K*, *SEC16B*, *TFAP2B*, *LINC00558*, *MC4R*, *GNPDA2*, and *GNAI3)* [7,8,10].

Overall, there was low heterogeneity between studies for the 25 SNPs, except for *FTO* (S2 Table) [29]. The broad age range included in the discovery meta-analysis of this study may conceal age-specific effects. Therefore, we performed a sensitivity analysis excluding studies of children aged <6 years (remaining N_sensitvity analysis_ = 55,354), which showed similar results (S6 Table) [30]. Additionally, we ran a sensitivity analysis excluding case-control studies and one excluding studies with a sample size <n = 500, showing similar results (S7 Table).

### Functional characterization

We used several strategies to gain insight into the functional characterization of the 25 SNPs leading the association signals with childhood BMI. A summary of relevant information from all strategies can be found in S8 Table.

First, we examined gene expression profiles of the nearest genes to the 25 SNPs from the combined meta-analysis with GTEx v7 in 53 tissues, using the tool FUMA [3,31]. We found differential expression of the 25 nearest genes in brain and salivary gland. In a second analysis of gene expression profiles in GTEx, we considered all genes in a region of 500 kb to either side of the 25 SNPs. Using this strategy, we additionally found differential expression in liver, heart, kidney, pancreas, muscle, skin and adipose tissue [31].

Second, we assessed whether the 25 SNPs were associated with gene expression in whole adipose tissue, isolated adipocytes, and isolated stroma-vascular cells from the Leipzig Adipose Tissue Childhood Cohort [32]. Full results can be found in S9 Table. We observed differential gene expression associated with multiple SNPs. Rs1094647 (nearest gene: *SLC45A3*) was associated with gene expression of *PM20D1* (P_FDR_ <0.05) in whole adipose tissue. We additionally found associations of rs114285994 (nearest gene: *GPRC5B*) with expression of *C16orf88* in isolated adipocytes. Rs115181845, which is in moderate LD (r^2^ = 0.47) with rs144376234 (nearest gene: *GNAI3*), was associated with expression of *GSTM1* and *GSTM2* in whole adipose tissue, isolated adipocytes and isolated stroma-vascular cells (S8 Table and S9 Table). No associations with gene expression were observed for any of the other 22 SNPs.

Third, we used Bayesian colocalization analysis to examine evidence for colocalization between GWAs and eQTL signals and to identify additional candidate genes for the 25 SNPs (GTEx v7). Briefly, GWAS summary statistics were extracted for each eQTL for all SNPs that were present in the meta-analysis and that were in common to both GWAS and eQTL studies. In most pairs, no evidence for association was found with either trait. To define colocalization we used restriction to pairs of childhood BMI and eQTL signals with a high posterior probability for colocalization (See Methods and Materials for details) [33]. We found significant colocalizations at 6 loci (*ADCY3*, *DNAJC27-AS1*, *CENPO*, *ADAM23*, *LIN7C*, *TFAP2B*) across a range of tissues (S8 Table and S10A and S10B Table) [8].

Fourth, to explore biological processes, we used DAVID, with the 25 nearest genes as input, using the Kyoto Encyclopedia of Genes and Genomes (KEGG) database [34,35]. Pathway analysis revealed one enriched biological process, cAMP signaling (P-value = 0.03).

Fifth, we performed a look-up in mouse-knockout data of the 25 nearest genes and, additionally, the genes that were indicated by colocalization and gene expression analysis. Mice in which *NEDD4L* was knocked out displayed neuronal abnormalities [36]. No related phenotypes were shown for *SLC45A3* or any of the 4 independent loci (*METTL1*, *PRRC2A*, *FAM150B*, and *MC4R*). Of the 19 known loci, *ADCY3* showed an association with increased total body fat in female heterozygous knockout mice, whereas *NEGR1* was associated with decreased lean body mass in male and female homozygous knockout mice (S8 Table). Full results can be found in S8 Table.

Sixth, among the 25 top SNPs, combined annotation-dependent depletion (CADD) scores >12.37, indicating potential pathogenicity of a SNP, were observed for rs13107325 (*SLC39A8*), rs56133711 (*BDNF*) and rs17817449 (*FTO*) (CADD scores of 34, 15.3 and 15.3, respectively) (S8 Table) [3,37].

### Genetic correlations of childhood BMI with adult phenotypes

**Fig 3:**
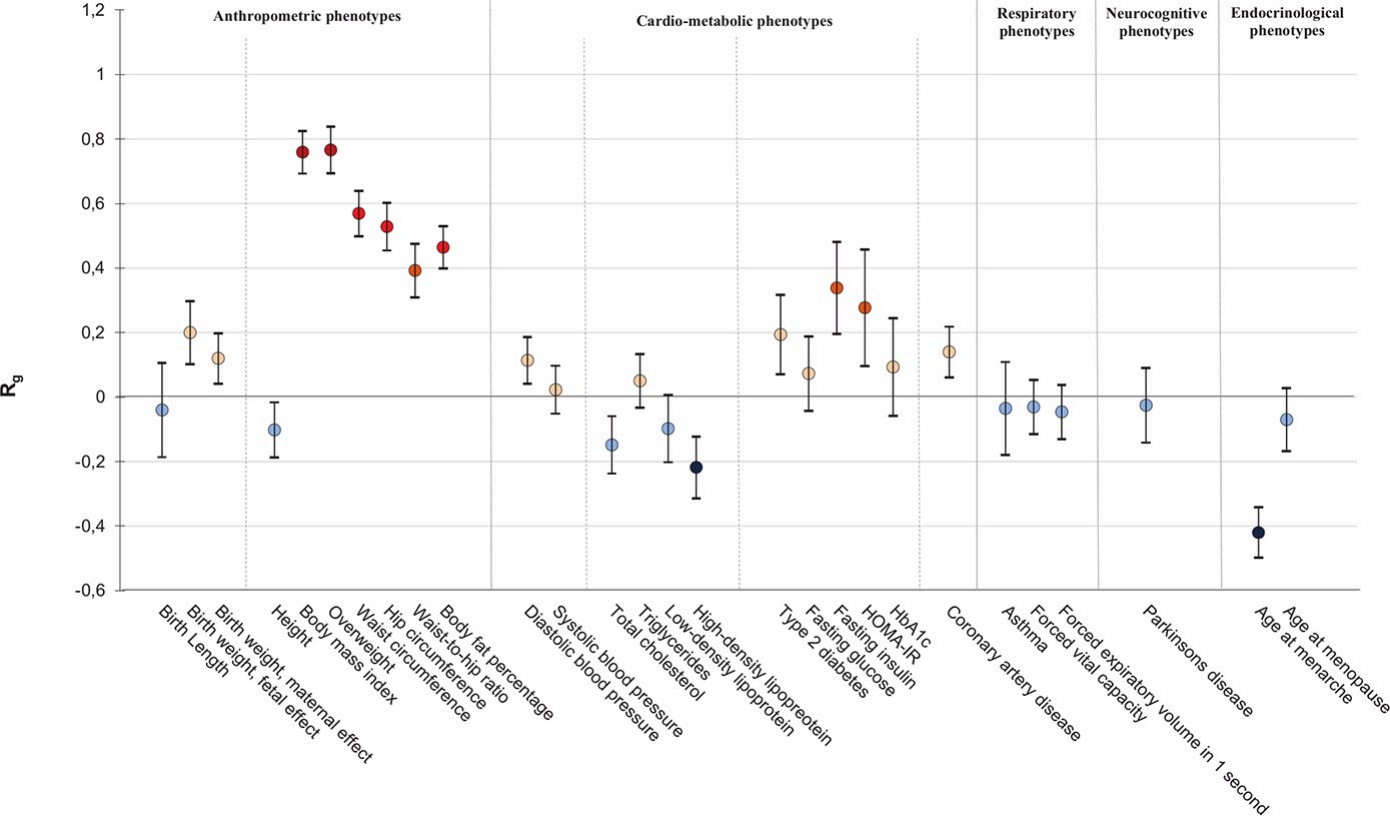
Genome-wide genetic correlations between childhood BMI and adult traits and diseases. On the x-axis the traits and diseases are shown. On the y-axis the genetic correlations (R_g_) and corresponding standard errors, indicated by error bars, between childhood BMI and each trait were shown, estimated by LD score regression. The genetic correlation estimates (R_g_) are colored according to their intensity and direction. Red indicates positive correlation, blue indicates negative correlation. References can be found in S11 Table.

First, to estimate the SNP heritability and the genetic correlations between childhood BMI and other traits from external GWAS meta-analysis data, we used LD-score regression [20]. SNP heritability was 0.23. There were positive genetic correlations of childhood BMI with several anthropometric and cardio-metabolic traits, including adult BMI (R_g_ = 0.76, P-value = 1.45 × 10^−112^), waist-to-hip ratio (R_g_ = 0.39, P-value = 1.57 × 10^−20^), body fat percentage (R_g_ = 0.46, P-value = 7.99 × 10^−44^), diastolic blood pressure (R_g_ = 0.11, P-value = 0.002), type 2 diabetes (R_g_ = 0.19, P-value = 0.002), and coronary artery disease (R_g_ = 0.14, P-value = 0.001) (Fig 3 and S11 Table). For birth weight, there were positive genetic correlations with childhood BMI, both when using fetal genetic effects on birth weight and when using maternal genetic effects on birth weight (R_g_ = 0.20, P-value = 3.19 x 10^−5^ and R_g_ = 0.12, P-value = 0.002 for fetal and maternal effects, respectively). Negative genetic correlations were observed between childhood BMI and total cholesterol (R_g_ = -0.15, P-value = 0.001), high-density lipoprotein (HDL) (R_g_ = -0.22, P-value = 8.65 x 10^−6^), and age at menarche (R_g_ = -0.42, P-value = 1.03 x 10^−32^). We did not find genetic correlations with any of the respiratory and neurocognitive phenotypes. Genetic correlations of childhood BMI with a selection of phenotypes that show evidence of association in observational studies are shown in Fig 3. Full results can be found in S11 Table.

Second, we did a look-up of the 25 SNPs in the adult BMI GWAS [7]. In total, 12 SNPs and 8 proxy SNPs (r^2^ ≥ 0.87) were available in the adult BMI study comprising ~700,000 individuals. No information was available on five loci, *FAM150B*, *GPR1*, *NEGR1*, *NEDD4L*, and *PRRC2A*. The directions of effect of all 20 SNPs were the same in adults as in children. Of these, 18 were genome-wide significantly associated with adult BMI (P-value <5 x 10^−8^) and the other 2 SNPs, *SLC45A3* and *METTL15*, showed suggestive evidence of association (P-values < 2.1 × 10^−6^) (S12 Table). Effect sizes of these 20 SNPs for adult BMI were highly correlated with those for childhood BMI (r^2^ = 0.86).

**Fig 4:**
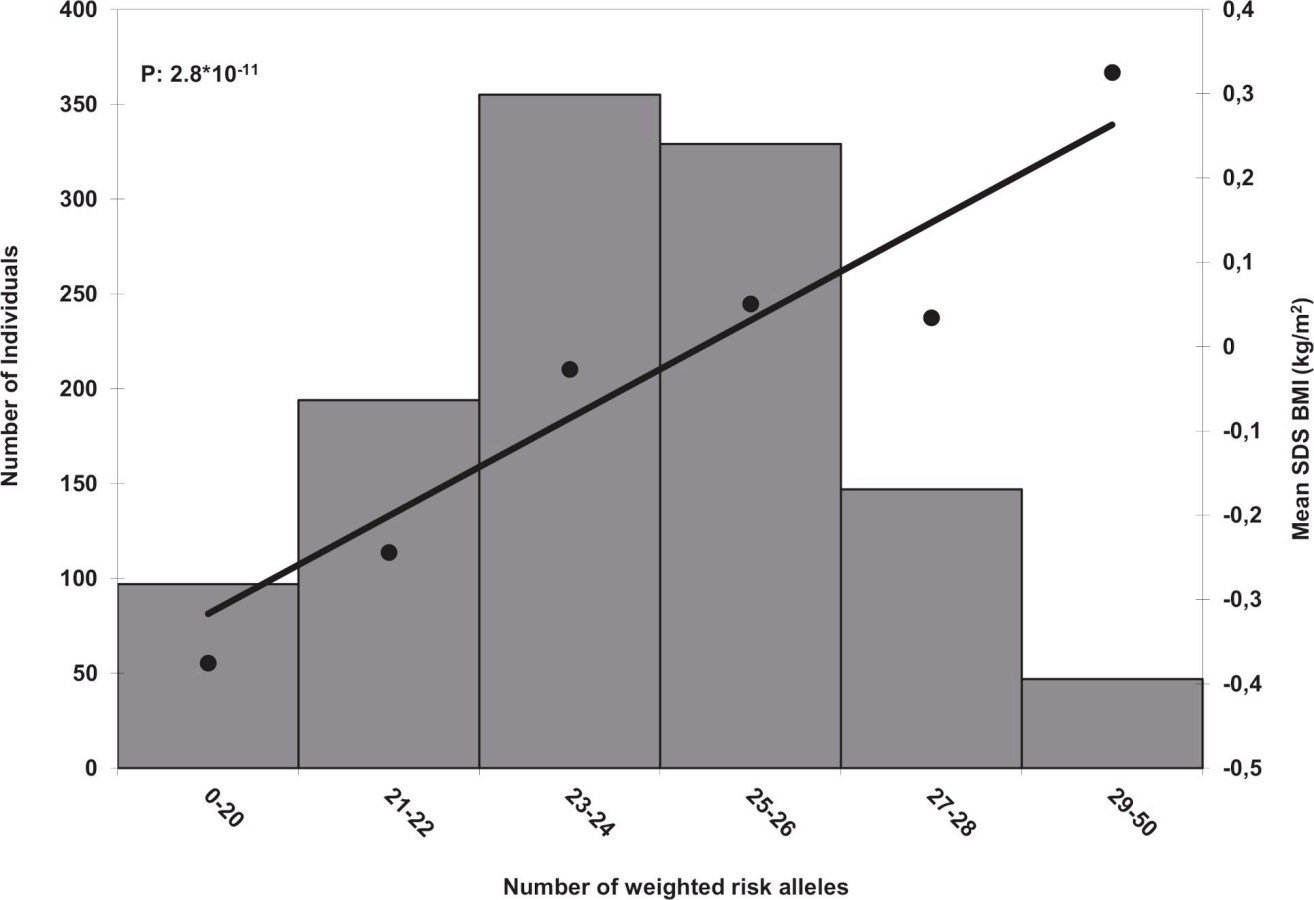
Associations of the weighted risk score with childhood BMI. Along the x-axis, categories of the risk score are shown together with the mean SDS BMI on the y-axis on the right and a line representing the regression of the mean SDS childhood BMI values for each category of the risk score. Along the y-axis on the left a histogram represents the number of individuals in each risk-score category. P-value is based on the continuous risk score. Analysis was performed in the TRAILS cohort (N = 1,169).

Third, we calculated a combined childhood BMI genetic risk score (GRS) of the 25 genome-wide significant SNPs, summing the number of BMI-increasing alleles weighted by their effect sizes from the combined meta-analysis. The GRS was associated with childhood BMI (*P-*value = 2.84 × 10^−11^) in 1,169 children from the Tracking Adolescents’ Individual Lives Survey (TRAILS) Cohort, aged 7 years, one of the largest replication cohorts (Fig 4). For each additional average risk allele in the GRS, childhood BMI increased by 0.06 SDS (SE = 0.009). This GRS explained 3.6% of the variance in childhood BMI. When calculating the risk score for the TRAILS cohort, effect estimates from the combined meta-analysis were used after excluding TRAILS from the meta-analysis. We additionally tested the GRS for association with adult BMI in the three sub-cohorts of the Rotterdam Study [38] (RS-I-1; n = 5,957, RS-II-1; n = 2,147 and RS-III-1; n = 2,998). We found the GRS to be associated with adult BMI in all study samples (P-values = 5.09 × 10^−9^, 0.02, and 1.49 × 10^−10^, respectively). Per additional average risk allele, adult BMI increased by 0.03 SDS (SE = 0.005), 0.02 SDS (SE = 0.009) and 0.04 SDS (SE = 0.007), explaining 0.6%, 0.2%, and 1.3% of the variance in adult BMI, respectively. No association was found of the GRS with birth weight and cardio-metabolic phenotypes, including insulin, triglycerides, low-density lipoprotein, HDL, total cholesterol, diastolic blood pressure and systolic blood pressure in 2,831 children aged 6 years from the Generation R Study if considering a Bonferroni corrected P-value of 0.00625 (S13 Table).

## Discussion

In this large GWAS meta-analysis of childhood BMI among >60,000 children aged 2–10 years, we identified 25 genome-wide significant loci. Two of these loci, rs1094647 near *SLC45A3* and rs184566112 near *NEDD4L* had not been associated with BMI before. We observed moderate to strong genetic correlations of childhood BMI with several anthropometric, cardio-metabolic, and endocrinological traits in adulthood, suggesting a shared genetic background.

The closest genes to the two novel loci, *SLC45A3* and *NEDDL4*, have not been strongly linked to obesity in previous studies and databases, indicating that functional studies are needed to identify possible biological pathways. *SLC45A3*, encoding the solute carrier family 45, member 3 protein, also known as prostate cancer-associated protein 6, has been related to prostate-specific antigen serum concentrations and prostate cancer [39–42]. *NEDD4L*, ubiquitin protein ligase Nedd4-like, known for its role in the regulation of ion channel internalization and turnover, is suggested to play a role in the regulation of respiratory, cardiovascular, renal, and neuronal functions [36,43–45]. The independent SNPs identified at loci known from previous studies on adult or childhood BMI may represent fully independent signals, although due to the low LD, these SNPs might still tag the same causal variant as the previously identified SNPs.

Since there is no strong previous evidence supporting the closest genes to the 25 SNPs as the causal genes, we took multiple approaches for further functional characterization. As many different tissues have been implicated to play a role in body composition we chose to include all available tissues in the gene expression analysis. Using GTEx, we found differential expression of the 25 nearest genes in brain. This may be of interest as appetite regulation might play a role in the development of obesity [46–48]. Gene expression data revealed an association between one of the novel SNPs, rs1094647 (nearest gene: *SLC45A3*), and expression of *PM20D1* in whole adipose tissue. *PM20D1*, Peptidase M20 domain-containing 1, previously identified as a factor secreted by thermogenic adipose cells, is known for its association with insulin resistance, glucose intolerance and enhanced defense of body temperature in cold when knocked out in mice. Furthermore, increased circulating PM20D1, together with adeno-associated virus-mediated transduction, leads to a higher energy expenditure and reduced adiposity in mice [49,50]. We used colocalization analysis to further identify candidate causal genes. This did not identify specific potential causal genes for rs1094647 (*SLC45A3*) and rs184566112 (*NEDD4L*). However, we identified *ADCY3*, *DNAJC27-AS1*, *CENPO*, *ADAM23*, *LIN7C*, *TFAP2B* as candidate genes for known loci across different tissues, including tibial nerve tissue, tibial artery tissue and the skin. No candidate genes were detected in biologically more relevant tissues, including subcutaneous or visceral adipose tissue.

Information on rs184566112 near *NEDD4L* was available in 24 out of 26 discovery cohorts that primarily used 1000 Genomes phase 1 imputed data (N = 37,104), thus clearly surviving our pre-set filter of having information in at least 50% of the number of studies and at least 50% of the total sample size in the discovery analysis. However, it was available in only less than half of the replication studies, mainly using 1000 Genomes phase 3 or HRC imputed data (N = 5,518) as this SNP was not included in these more recent reference panels. No other SNPs in high LD were available as proxy for this SNP in the replication analysis. Therefore, this signal needs to be interpreted with caution. However, no heterogeneity of this SNP between the discovery stage studies (*I*^2^ = 0; P-value for heterogeneity = 0.98), a high imputation quality (weighted mean R^2^ = 0.89) and the known association of another locus in the same region with adult body fat percentage might lends credibility to this signal, although further work is needed to unravel the details [27]. Previous studies have shown that variants might have strong age-dependent effects across childhood [15,51]. We performed a sensitivity analyses excluding children aged <6 years, as the approximate age of the adiposity rebound [30]. However, no difference in main results with the full meta-analysis were observed.

Observational studies suggest that childhood obesity is not only related to several anthropometric and cardio-metabolic phenotypes in later life, such as type 2 diabetes, but also to respiratory and neurocognitive traits, including asthma and Parkinson’s disease and to a lower age at menarche [14–18,52–58]. In observational studies, effect estimates may be influenced by confounding factors and reverse causation, potentially evoking spurious associations [59,60]. Genetic studies can provide more insight into the etiology of complex diseases. We observed a strong positive genetic correlation of childhood BMI with adult BMI. This is in line with previous studies [8,20,21]. We additionally observed positive genetic correlations between childhood BMI and several cardio-metabolic phenotypes in later life, including waist-to-hip ratio, diastolic blood pressure, type 2 diabetes, and coronary artery disease. Negative genetic correlations were found between childhood BMI and HDL-C and age at menarche. These results may suggest that the associations reported in observational studies are partly explained by genetic factors [15–17,58,61]. However, there is also evidence from previous work to support that the associations of childhood BMI with cardiometabolic phenotypes in adulthood are explained by the continuity of a high BMI from childhood until later ages, rather than by an independent effect of childhood BMI on adult cardiometabolic phenotypes [15,62]. From our data, we are not able to distinguish this. Childhood BMI was not genetically correlated with asthma and Parkinson’s disease. This may indicate that the observational associations between childhood BMI and these phenotypes are not strongly explained by shared genetics [18,56,57,63].

The GRS combining the 25 top SNPs was not associated with cardiometabolic phenotypes in children aged 6 years. This may indicate that there is no shared genetic basis between childhood BMI and these phenotypes in childhood. However, the GRS analyses in children had a much lower sample size than the LD score regression analyses in adults and phenotypic variation in these phenotypes is more limited in children, leading to a much lower power to detect associations in these analyses. Additionally, the GRS was composed of the top-associated SNPs, whereas the genetic correlation estimated from the LDSR examined variation genome-wide.

We observed a SNP heritability of 0.23 which is consistent with previous findings [4–6].

Secular trends in obesity across populations and age groups can influence the heritability estimates across distinct population settings, requiring careful interpretation. This contention is also relevant for the interpretation of the genetic correlations estimated between traits. Environmental influences like those giving rise to the increase obesity in the last decades, can influence heritability estimates and hence, the power to identify significant genetic correlations. Before concluding unequivocal absence of some degree of “shared heritability” between childhood BMI and some of the adult traits, genetic correlations should be interpreted in the context of power limitations. Increasingly larger environmental influences along the life-course can result in lower heritability, but recent work has also shown that the increase in phenotypic variance accompanying increasing prevalence of obesity occurs alongside an increase in genetic variance [64–67]. This results in relatively stable (broad sense) heritability estimates across measurement years, as recently shown by a large-scale meta-analysis of adult twin data.

The 25-SNP GRS was positively associated with both childhood and adult BMI, showing slightly larger effect estimates in children suggesting that these specific genetic variants affect BMI in both childhood and adulthood, but with stronger effects at younger ages. A recent study, using genome-wide polygenic scores of 2.1 million common variants, found that the overall effect of those variants on weight starts in early childhood and increases over time [4]. Two previous studies also describe specific genetic variants associated with BMI in infancy only, and overlapping patterns of genetic variants with those in adults emerging from childhood onwards. Three SNPs associated with infant BMI from these studies were not genome-wide significantly associated with childhood BMI in our data (P-values >0.02), which supports their infancy-specific effects [68,69].

Although many of the associated variants from the current study overlap between children and adults, the relative order of the signals differs. Additionally, *SLC45A3*, one of the novel loci did not show genome-wide association in adult data [7]. However, suggestive association of this locus with adult BMI was observed (P-value = 2.7 x 10^−5^). Overall, the effect estimates of the 25 SNPs in childhood were highly correlated with those in adulthood (r^2^ = 0.86). Taken together, evidence from the current and previous studies suggests that biological processes underlying BMI are similar from childhood onwards, but their relative influence may differ depending on the life stage.

## Conclusions

In conclusion, we identified 25 loci for childhood BMI, together explaining 3.6% of the variance in childhood BMI. Two of these are novel and four represent independent SNPs at loci known to be associated with adult or childhood BMI. A strong positive genetic correlation of childhood BMI with adult BMI and related cardio-metabolic phenotypes was observed. Our results suggest that the biological processes underlying childhood BMI largely, but not completely, overlap with those underlying adult BMI. The well-known observational associations of BMI in childhood with cardio-metabolic diseases in adulthood may reflect partial genetic overlap, but in light of previous evidence, it is also likely that they are explained through phenotypic continuity of BMI from childhood into adulthood.

## Materials and methods

### Ethics statement

All individual studies got approval by their medical ethics review committees. All participants gave written informed consent. Study-specific ethics statements are given in S1 Text.

### Study design

We conducted a two-stage meta-analysis in children of European ancestry to identify genetic loci associated with childhood BMI. Sex- and age-adjusted standard deviation scores were created for BMI at the latest time point (oldest age, if multiple measurements were available) between 2 and 10 years using the same software and external reference across all studies (LMS growth; Pan H, Cole TJ, 2012; http://www.healthforallchildren.co.uk). In the case of twin pairs and siblings, only one of each twin or sibling pair was included, either randomly or based on genotyping or imputation quality.

In the discovery stage, we performed a meta-analysis of 26 studies (N = 39,620), including the Avon Longitudinal Study of Parents and Children (ALSPAC, N = 6790), the Bone Mineral Density in Childhood Study (BMDCS, N = 543), BRain dEvelopment and Air polluTion ultrafine particles in scHool childrEn (BREATHE, N = 1633), the Children’s Hospital of Philadelphia (CHOP, N = 5488), the Copenhagen Prospective Studies on Asthma in Childhood 2000 (COPSAC2000, N = 327), the Copenhagen Prospective Studies on Asthma in Childhood 2010 (COPSAC2010, N = 571), the Danish National Birth Cohort- preterm birth study (DNBC-PTB, N = 1007), the French Young Study (French young, N = 304 cases and N = 144 controls), the Generation R Study (GenerationR, N = 2071), the Genetics of Overweight Young Adults (GOYA Male, N = 319), the Helsinki Birth Cohort Study (HBCS, N = 1575), the INfancia y Medio Ambiente [Environment and Childhood] Project, with two subcohorts that were entered into the meta-analysis seperately (INMA-Sabadell and Valencia subcohort, N = 650, and INMA-Menorca subcohort, N = 300), German Infant Study on the influence of Nutrition Intervention PLUS environmental and genetic influences on allergy development & Influence of life-style factors on the development of the immune system and allergies in East and West Germany (GINIplus&LISA, N = 1471), the Manchester Asthma and Allergy Study (MAAS, N = 784), the Norwegian Mother, Father and Child Cohort (MoBa, N = 522), the Northern Finland Birth Cohort 1966 (NFBC 1966, N = 3949), the Northern Finland Birth Cohort 1986 (NFBC 1986, N = 1056), the Netherlands Twin Register (NTR, N = 1767), the Physical Activity and Nutrition in Children Study (PANIC, N = 374), The Danish Childhood Obesity Data and Biobank (TDCOB, N = 158 controls), the Raine Study (the Raine Study (Generation 2), N = 1458), the Special Turku Coronary Risk factor Intervention Project (STRIP, N = 551), the Young Finns Study (YFS, N = 1134), the TEENs of Attica: Genes and Environment (TEENAGE, N = 252), and the British 1958 Birth Cohort Study (1958BC-T1DGC, N = 2081 and 1958BC-WTCCC, N = 2341).

In the replication stage, we included 14 studies (n = 21,491), which did not have genome-wide association data available at the time of the discovery analysis: 888 additional children from the Danish National Birth Cohort- Goya offspring (N = 407 offspring from obese mothers, N = 481 offspring from randomly selected mothers), 294 additional children from the INfancia y Medio Ambiente [Environment and Childhood] (INMA) Project (INMA- Gipuzkoa subcohort, N = 314), 6,828 additional children from the Norwegian Mother, Father and Child Cohort (MoBa, N = 6828), 753 additional children from TDCOB (N = 344 controls and N = 409 cases), the Amsterdam Born Children and their Development- Genetic Enrichment (ABCD, N = 1154), The European Childhood Obesity Project (CHOP Study, N = 369), the Family Atherosclerosis Monitoring In earLY life (FAMILY) study (the FAMILY study, N = 543), the Etude des Déterminants pré- et postnatals précoces du développement et de la santé de l'Enfant (EDEN) mother-child cohort (EDEN, N = 821), the Exeter Family Study of Childhood Health (EFSOCH, N = 542), the Leipzig Research Center for Civilization Diseases—Child study (LIFE-Child, N = 1318), the Prevention and Incidence of Asthma and Mite Allergy study (PIAMA, N = 1958), the Screening of older women for prevention of fracture Study (SCOOP, N = 685), the Småbørns Kost Og Trivsel study (SKOT1, N = 236), the Twin Early Development Study (TEDS, N = 3933), the Tracking Adolescents’ Individual Lives Survey cohort (TRAILS-population cohort, N = 1169). In the EDEN mother-child cohort, information was available about three SNPs only (rs7138803, rs13107325, and rs987237). Characteristics of discovery and replication studies can be found in S1 Table and S1 Text.

### Study-level analyses

Genome-wide association analyses were first run in all discovery cohorts separately. Studies used high-density Illumina or Affymetrix SNP arrays, followed by imputation to the 1000 Genomes Project or HRC. Before imputation, studies applied study specific quality filters on sample and SNP call rate, minor allele frequency and Hardy–Weinberg disequilibrium (see S1 Table for details). Linear regression models assuming an additive genetic model were run in each study to assess the association of each SNP with BMI SDS, adjusting for principal components if this was deemed needed in the individual studies. As BMI SDS is age and sex specific, no further adjustments were made. Before the meta-analysis, we applied quality filters to each study, filtering out SNPs with a minor allele frequency (MAF) below 1% and SNPs with poor imputation quality (MACH r2_hat ≤0.3, IMPUTE proper_info ≤0.4 or info ≤0.4).

### Meta-analysis

We performed fixed-effects inverse-variance weighted meta-analysis of all discovery samples using Metal [70]. Genomic control was applied to every study before the meta-analysis. Individual study lambdas before genomic control ranged from 0.993 to 1.036 (S1 Table). The lambda of the discovery meta-analysis is shown in S1 Fig. After the meta-analysis, we excluded SNPs for which information was available in less than 50% of the studies and less than 50% of the total sample size. We report I2 and p-value for heterogeneity for all findings.

The final dataset consisted of 8,228,795 autosomal SNPs. Genome-wide Complex Trait Analysis (GCTA) was used to select the independent SNPs for each locus [22]. We performed conditional analyses based on summary-level statistics and LD estimation between SNPs from the Generation R Study as a reference sample to select independently associated SNPs based on conditional P-values [22]. Forty-seven genome-wide significant or suggestive loci (P-values <5 × 10^−8^ and <5 × 10^−6^, respectively) were taken forward for replication in 14 replication cohorts. Fixed-effects inverse variance meta-analysis was performed for these 47 SNPs combining the discovery samples and all replication samples, giving a combined analysis beta, standard error and P-value (Table 1). SNPs that reached genome-wide significance in the combined analysis were considered to be genome-wide significant.

### Functional mapping and annotation of genetic associations (FUMA)

To obtain predicted functional consequences for our 25 SNPs, we used SNP2FUNC in FUMA, a web-based platform to facilitate and visualize functional annotation of GWAS results (http://fuma.ctglab.nl) [3]. By matching chromosome, position, and reference and alternative alleles, combined annotation-dependent depletion (CADD) scores were annotated, indicating the deleteriousness of a SNP [37].

To annotate the nearest genes of the 25 SNPs in biological context, we used the GENE2FUNC option in FUMA, which provides hypergeometric tests of enrichment of a list of genes in 53 GTEx tissue-specific gene expression sets (GTEx v 7) [3,31]. We used GENE2FUNC for two sets of genes: 1. Nearest genes of 25 SNPs; 2. Genes located in a region of 500 kb to either side of the 25 SNPs.

### Look-up of the 25 SNPs in expression data

We studied the associations of the 25 SNPs associated with childhood BMI with gene expression levels in adipose tissue samples from the Leipzig Adipose Tissue Childhood Cohort [32]. These associations were examined in the following tissues: whole adipose tissue, isolated adipocytes and isolated stroma-vascular cells using genome- wide expression analysis (Illumina HumanHT-12 v4 arrays). Gene expression raw data of all 47,231 probes was extracted by Illumina GenomeStudio without additional background correction. Data was further processed within R / Bioconductor. Expression values were log2-transformed and quantile-normalised [71,72]. Batch effects of expression BeadChips were corrected using an empirical Bayes method [73].Within pre-processing, gene-expression probes detected by Illumina GenomeStudio as expressed in less than 5% of the samples were excluded as well as probes still found to be significantly associated with batch effects after Bonferroni-correction. Furthermore, gene-expression probes with poor mapping on the human trancriptome [74] were also excluded. In summary, these filters resulted in 23354, 21258, and 22637 valid gene-expression probes from which 20672, 18956, and 20230 probes corresponded to 14455, 13518, and 14256 genes mapping to a unique position in the human genome (hg19) for whole adipose tissue, adipocytes, and stroma/vascular cells, respectively. Three criteria were used to remove samples of low quality: First, the number of detected gene-expression probes of a sample was required to be within ± 3 interquartile ranges (IQR) from the median. Second, the Mahalanobis distance of several quality characteristics of each sample had to be lower than median + 4 x IQR. Third, Euclidean distances of expression values as described [71] had to be lower than median + 4 x IQR. Overall, of the assayed samples, 2, 4, and 2 samples were excluded for quality reasons leaving 203, 63 and 69 unique individuals having also valid data for eQTL analysis for whole adipose tissue, adipocytes, and stroma/vascular cells, respectively. Associations between the genotype and gene expression of genes in *cis* (respective gene area +/- 1 Mb regarding transcription start and transcription end) were analyzed using a gene-dose based linear regression model adjusted for age and sex as implemented in MatrixEQTL [75]. Analysis of variants within one haplotype were done through analyses of linkage-disequilibrium using 1000 Genomes Phase 1 Version 3 and HapMap r28 hg19 CEU as references.

### Colocalization analysis

We used Bayesian colocalization analysis to examine evidence for colocalization between childhood BMI and eQTL signals (GTEx v7).Colocalization analyses were conducted using the R package coloc, hht://cran.r-project.org/web/packages/coloc, as described previously [33]. Briefly, in each of the GTEx v7 tissues, all cis-eQTLs at FDR <5% were identified. For each eQTL, GWAS summary statistics were extracted for all SNPs that were present in >50% of the studies and >50% of the total sample size and that were in common to both GWAS and eQTL studies, within 1 MB of the transcription start site of the gene. For each such locus, colocalization analyses were done with default parameters, testing the following hypotheses [33]:

H_0_: No association with either trait;
H_1_: Association with childhood BMI only;
H_2_: Association with gene expression only;
H_3_: Association with childhood BMI and gene expression, two distinct causal variants;
H_4_: Association with childhood BMI and gene expression, one shared causal variant.

Support for each hypothesis was quantified in terms of posterior probabilities, defined at SNP level and indicated by PP_0_, PP_1_, PP_2_, PP_3_ or PP_4_, corresponding to the five hypotheses and measuring how likely these hypotheses were. S10B Table shows the above-mentioned posterior probabilities for all pairs. In most pairs, no evidence for association was found with either trait. In case association was observed, it was mostly with a single trait. To define colocalization we used restriction to pairs of childhood BMI and eQTL signals with a high posterior probability for colocalization, indicated by a PP4/(PP3+PP4) >0.9 (S10A Table).

### DAVID

To explore biological processes, we used DAVID, with the 25 nearest genes as input, using the Kyoto Encyclopedia of Genes and Genomes (KEGG) database [34,35].

### Linkage-disequilibrium score regression

The use of LD score regression to estimate genetic correlations between two phenotypes has been described in detail previously [20]. Briefly, LD score is a measure of how much a genetic variation is tagged by each variant. A high LD score indicates that a variant is in high LD with many nearby polymorphisms. Variants with high LD scores are more likely to contain true signals and have a higher chance of overlap with genuine signals between GWAS. To estimate LD scores, summary statistics from GWAS meta-analysis are used to calculate the cross-product of test statistics per SNP, which is regressed on the LD score. The slope of the regression is a function of the genetic covariance between traits [20]:

$$E(z1jz2j)=\frac{\sqrt{N1N2\rho g}}{M}lj+\frac{\rho Ns}{\sqrt{N1N2}}$$

where *N*_*i*_ is the sample size of study *i*, *ρ*_*g*_ is the genetic covariance, *M* is the number of SNPs in the reference panel with a MAF between 5% and 50%, *l*_*j*_ is the LD score for SNP *j*, *N*_*s*_ quantifies the number of individuals that overlap both studies, and ρ is the phenotypic correlation amongst the *N*_*s*_ of overlapping samples. A sample overlap or cryptic relatedness between samples will only affect the intercept from the regression but not the slope. Estimates of genetic covariance will therefore not be biased by overlapping samples. Similarly, in case of population stratification, the intercept will be affected but it will have only minimal impact on the slope since population stratification does not correlate with LD between variants.

Because of the correlation between the imputation quality and LD score, imputation quality is a confounder for LD score regression. Therefore, SNPs were excluded for the following reasons: MAF <0.01 and INFO ≤0.9. The filtered GWAS results were uploaded on http://ldsc.broadinstitute.org/ldhub/, a website with many GWAS meta-analyses available on which LD score regression has been implemented by the developers of the LD score regression method. In case multiple GWAS meta-analyses were available for the same phenotype, the genetic correlation with childhood BMI was estimated using the most recent meta-analysis. Genetic correlations are shown in Fig 2 and S11 Table.

### Genetic risk score and percentage of variance explained

We combined the 25 genome-wide significant SNPs from the combined meta-analysis into a GRS by summing up the number of BMI SDS-increasing alleles, weighted by the effect sizes from the combined meta-analysis. The GRS was rescaled to a range from 0 to 50, which is the maximum number of BMI SDS increasing alleles and rounded to the nearest integer. Linear regression analysis was used to examine the associations of the risk score with childhood and adult BMI. For these analyses data from the TRAILS cohort (N = 1169), one of the largest replication cohorts, and data from the Rotterdam Study (RS-I-1; n = 5,957, RS-II-1; n = 2,147 and RS-III-1; n = 2,998) were used. Additionally, linear regression analysis was used to examine the associations of the GRS with birth weight and childhood metabolic phenotypes in Generation R in which detailed information on these phenotypes was available. When calculating the risk score for the TRAILS cohort and Generation R, effect estimates from the combined meta-analysis were used after excluding TRAILS and Generation R, respectively, from the meta-analysis. The variance explained was estimated by the adjusted R^2^ of the models.

## Supporting information

S1 Table Descriptive characteristics of studies–*Separate Excel sheets*. (XLSX)

S2 Table Results of the discovery, replication and combined analyses for all 47 independent loci with *P-*values <5 x 10^−6^ in the discovery analysis*—Separate Excel sheet*. (XLSX)

S3 Table Directions of effect for the individual discovery and replication studies for all 47 loci with *P*-values <5 x 10^−6^ in the discovery analysis. (DOCX)

S4 Table Results of the discovery analysis for all SNPs with *P*-values <5 x 10^−6^- *Separate Excel sheet*. (XLSX)

S5 Table Descriptive characteristics of studies with data on fat mass percentage available–*Separate Excel sheets*. (XLSX)

S6 Table Results for the 25 SNPs with *P*-values <5 x 10^−8^ in the combined analysis, restricted to children aged ≥ 6 years. (DOCX)

S7 Table Results of the combined analyses for the 25 SNPs excluding case-control studies and excluding studies with a sample size smaller than n = 500. (DOCX)

S8 Table Look up of the 25 nearest genes and candidate genes indicated by colocalization analysis- *Separate Excel sheet*. (XLSX)

S9 Table Expression levels of the 25 childhood BMI SNPs in the Adipose Tissue Childhood Cohort- *Separate Excel sheet*. (XLSX)

S10 Table Colocalization analyses with GTEx v7—*Separate Excel sheet*. (XLSX)

S11 Table Summary results of LD score regression analyses between childhood BMI and various metabolic, cardiovascular and lifestyle-related phenotypes. (DOCX)

S12 Table Results of adult BMI GWAS meta-analysis for 20 loci with *P-*values <5 x 10^−8^ in the combined analysis. (DOCX)

S13 Table Results of the GRS for birth weight and childhood cardio-metabolic phenotypes. (DOCX)

S1 Fig Quantile-Quantile plot of the SNPs in the discovery meta-analysis of 26 studies. (TIF)

S2 Fig Locus zoom plots of the 4 independent SNPs at known loci. (TIF)

S1 Text Cohort information. (DOCX)
