# Supplementary material for: Novel loci for childhood body mass index and shared heritability with adult cardiometabolic traits
Source: PLoS Genet. 2020 Oct 12;16(10):e1008718. doi: 10.1371/journal.pgen.1008718 (PMC7581004; doi:10.1371/journal.pgen.1008718)
Supplement: S3 Table — (DOCX) [file pgen.1008718.s003.docx]

**S3 Table** Directions of effect for the individual discovery and replication studies for all 47 loci with

*P*-values < 5 x 10^-6^ in the discovery analysis

| **SNP** | **CHR** | **Position** | **Nearest gene** | **EA/**  **Non-EA** | **Direction of effect discovery analysis** | **Direction of effect replication analysis** | **P–value combined analysis** |
| --- | --- | --- | --- | --- | --- | --- | --- |
| rs11676272^a,b^ | 2 | 25141538 | *ADCY3* | G/A | +++??++--+++?++++++++?++?+++ | ++++++-++-+++?++++ | **3.79 x 10^-30^** |
| rs7138803^a,b^ | 12 | 50247468 | *BCDIN3D* | A/G | +?+??+-+++?+?+++++++++++++++ | +++++++++++-++++++ | **4.23 x 10^-30^** |
| rs939584^a,b,c^ | 2 | 621558 | *TMEM18* | T/C | ++++++++++++++++++++-?++?+++ | +++++--++++++?++++ | **3.73 x 10^-29^** |
| rs17817449^a,b^ | 16 | 53813367 | *FTO* | G/T | +?+??+---+?+?+-++++-++++++++ | ++-++-+++-+++?++++ | **2.98 x 10^-27^** |
| rs12042908^a,b^ | 1 | 74997762 | *FPGT-TNNI3K, TNNI3K* | A/G | ++++++-+++-++++?+?++++++++-+ | +++++++++++++?+++- | **6.37 x 10^-25^** |
| rs543874^a,b^ | 1 | 177889480 | *SEC16B* | G/A | +?+??+++++?+?+++++++++-+++-+ | -------------?++-+ | **6.02 x 10^-22^** |
| rs56133711^a^ | 11 | 27723334 | *BDNF* | A/G | +-+-++-+++++++++?+++++++++++ | ++--++++++++-?++++ | **3.75 x 10^-15^** |
| rs2076308^a,b^ | 6 | 50791640 | *TFAP2B* | C/G | ++++-+-++--++++?+?-+++++++-+ | +--+++-++-++-?-+++ | **3.07 x 10^-13^** |
| rs4477562^a,b,d^ | 13 | 54104968 | *LINC00558* | T/C | ++++++++-+++++-+++++++++++++ | +++++-+-++-+-?+-+- | **5.81 x 10^-13^** |
| rs571312^a,b^ | 18 | 57839769 | *MC4R* | A/C | +?+??++-++?+?++-++-+-+++++++ | ++-+-+-++++++?++++ | **8.80 x 10^-13^** |
| rs12641981^a,b^ | 4 | 45179883 | *GNPDA2* | T/C | +?+??+---+++?+++?++++?++?+++ | ++-++++++-++-?++++ | **1.29 x 10^-12^** |
| rs62107261^e^ | 2 | 422144 | *FAM150B* | T/C | +-++-+++-?-+-+?-?+-+++++++++ | +-+++--++++++?+-++ | **9.93 x 10^-12^** |
| rs114285994^a^ | 16 | 19935763 | *GPR0C5B* | G/A | +++++++-++++-++++++++?++?+++ | +------++++++?++++ | **1.11 x 10^-11^** |
| rs144376234^a,b^ | 1 | 110114504 | *GNAI3* | T/C | ++++-++++-++++-+++-+++-+++++ | -+--++--+++++?++++ | **1.38 x 10^-10^** |
| rs1094647 | 1 | 205655378 | *SLC45A3* | G/A | +++++++-++-++--++++++-++++++ | +++-+--++++++?++++ | **7.20 x 10^-10^** |
| rs76227980^e^ | 18 | 58036384 | *MC4R* | C/T | ++++++-++++??+-++++-+?+-?-++ | +++----++-++-?+?+? | **8.68 x 10^-10^** |
| rs13107325^a^ | 4 | 103188709 | *SLC39A8* | T/C | +++++++-++?-?++??+-++-++-+++ | +++++--++--+-+--++ | **1.38 x 10^-9^** |
| rs62500888^b^ | 8 | 28061823 | *ELP3* | A/G | +++--++-+-+++++++-+-+-++++++ | +++-+-+++++-+?+-++ | **1.81 x 10^-9^** |
| rs114670539^b^ | 2 | 207064335 | *GPR1* | T/C | +-++++--++-+-+++++++--+++++- | ++++-+-++-++??++-- | **1.92 x 10^-9^** |
| rs61765651^a^ | 1 | 72754314 | *NEGR1* | C/T | ++++-++-++-++++?+?+++++++-++ | +++-++-++---+?-+++ | **4.99 x 10^-9^** |
| rs7719067^a^ | 5 | 153538241 | *GALNT10* | A/G | +++++++--+++++++-+++++++++-+ | +-+?????+-+++?+-++ | **6.54 x 10^-9^** |
| rs11030391^e^ | 11 | 28644626 | *METTL15* | A/G | ++++++--+-++-+++-+-++-++-+-- | -++-++++++++-?++++ | **1.51 x 10^-8^** |
| rs184566112 | 18 | 55943926 | *NEDD4L* | A/T | ++++++++++++?+++?++++-++-+-+ | +??++++++?+??????? | **4.24 x 10^-8^** |
| rs116664060 | 6 | 31592524 | *PRRC2A* | C/G | +?+-+++-+++??-?+?++-+??+?+++ | +++-++-++---+?-+++ | **4.63 x 10^-8^** |
| rs11215427^a^ | 11 | 115093438 | *CADM1* | G/C | +++++-+++++++-++?-++-+++++++ | +++??????-++-?+-+- | **4.64 x 10^-8^** |
| rs1336980^b^ | 9 | 129377855 | *LMX1B* | C/G | ++++++++++-+--+-+++++?++?-++ | ---++-+-+-++-?+++- | 1.17 x 10^-7^ |
| rs146823532^e^ | 1 | 74979126 | *FPGT-TNNI3K, TNNI3K* | A/G | +++-+++++?++++?+?-?-++++++-+ | -++--+++++++???+++ | 1.33 x 10^-7^ |
| rs79386556 | 13 | 71229046 | *LINC00348* | A/G | +-+++++-++-??++?-?-++-+++--+ | ++-++-+++-+-??+-++ | 4.83 x 10^-7^ |
| rs9942489 | 6 | 35323709 | *PPARD* | A/T | +++-++++-++++-+?++-+--+++?++ | --+++-+--++++?-+++ | 5.56 x 10^-7^ |
| rs17086809 | 9 | 86708695 | *RMI1* | C/T | ++++++++++-+++++?+++-?++?+-+ | +++--+++-+---?--+- | 9.30 x 10^-7^ |
| rs80332495 | 5 | 19191677 | *CDH18* | A/G | +++--++?++++++?+++++-+++++-+ | +-++++-----++?+-+- | 1.14 x 10^-6^ |
| rs4594227 | 15 | 84497207 | *ADAMTSL3* | A/G | ++++-++++++-+++++++++++--+++ | -+-++++++-+--?+-+- | 1.75 x 10^-6^ |
| rs2457463 | 10 | 70315687 | *TET1* | G/T | ?++??+++??++?+???+++--+-++++ | +???????-????????? | 2.69 x 10^-6^ |
| rs11865086 | 16 | 30130493 | *MAPK3* | C/A | +++-++++++-+++++++-+++++-+-+ | ---++++-+--++?+++- | 3.31 x 10^-6^ |
| rs1565356 | 6 | 34046065 | *GRM4* | C/A | +++-++++++++-++++-+++?++?+++ | ----+-+--+-+-?+?-+ | 4.01 x 10^-6^ |
| rs2952863^a^ | 4 | 130759647 | *C4orf33* | T/G | ++++-+-+++-+-+++?++-+++--+-+ | -++-----++--+?++++ | 4.27 x 10^-6^ |
| rs2358954 | 12 | 66379504 | *HMGA2* | T/G | +-+-+++++-++++++?+++-+-+++++ | -++-++-++++--?+-++ | 4.77 x 10^-6^ |
| rs7652876 | 3 | 179831733 | *PEX5L* | A/C | +-+++++-+-+++-+++-+++++-+++- | -++++?+++++-+?---+ | 6.93 x 10^-6^ |
| rs4923207 | 11 | 24757325 | *LUZP2* | T/G | +-++++++++-+--+-?+++++++-+++ | -??-+---+-++??-+?+ | 7.05 x 10^-6^ |
| rs7757288^a^ | 6 | 55205502 | *GFRAL* | G/A | +-++-+++-++++++-?+-++?++?-++ | -++-+++--+++-?+-+- | 1.03 x 10^-5^ |
| rs6876477 | 5 | 50878621 | *ISL1* | A/T | +-+++++-+++++++-++++-+++++++ | -+-+++-++-++-?+-++ | 1.31 x 10^-5^ |
| rs28599560 | 5 | 91791853 | *FLJ42709* | A/G | ++++-++-++++?++-+--+++++++++ | ++++-+--+++-+?--++ | 3.48 x 10^-5^ |
| rs117281273 | 8 | 42981400 | *SGK196* | C/G | +-++++++++++++?-+--+++++-+++ | --+--+-+++++??+--- | 3.48 x 10^-5^ |
| rs9695734 | 9 | 96407983 | *PHF2* | C/T | +++??+-++-?+?+-+++++++++-++- | ++-+-+-+++-+-?--++ | 4.99 x 10^-5^ |
| rs72833479 | 17 | 45960449 | *SP2* | A/G | ++++-+++++-+++++++++++++++++ | ----+---+--++?-++- | 6.54 x 10^-5^ |
| rs142367753 | 2 | 128938956 | *UGGT1* | C/G | +++--++++-?+++?++++?+?+?-+?+ | +---??-?-+--+?++-? | 1.34 x 10^-3^ |
| rs6896578 | 5 | 76423090 | *ZBED3-AS1* | C/T | ++++-+++++++++-+?--++++--+-+ | +-+---+--++--?-+-+ | 4.1 x 10^-3^ |

Direction of the effect for the effect allele for each individual study is shown: + indicates a positive effect estimate for the effect allele, - indicates a negative effect estimate for the effect allele, ? indicates no information available. Order of the studies in the discovery analysis: ALSPAC, BMDCS, CHOP, COPSAC2000, COPSAC2010, 1958BC-T1DGC, French Young Study- cases, French Young study- controls, GINIplus&LISA, GOYA male, TDCOB controls, NFBC1966, NFBC1986, HBCS, NTR, PANIC, Raine Study, STRIP, TEENAGE, 1958BC-WTCCC, MoBa, INMA Sabadell and Valencia, BREATHE, DNBC-PTB, INMA Menorca, YFS, MAAS, Generation R. Order of the studies in the replication analysis: Trails, TEDS OEE, TEDS AFFY, TDCOB controls, TDCOB cases, SKOT1, DNBC-GOYA offspring from obese mothers, DNBC-GOYA offspring from randomly selected mothers, Leipzig, EFSOCH, ABCD, CHOP, the FAMILY study, EDEN, PIAMA, SCOOP, MoBa, INMA Gipuzkoa.

^a^ Locus previously reported for adult BMI

^b^ Locus previously reported for childhood BMI

^c^ Locus previously reported for adult body fat
^d^ Locus previously reported for childhood obesity
^e^ Independent SNP at the same locus selected by conditional analysis

CHR: Chromosome; EA: Effect Allele; SE: Standard Error
