# Supplementary material for: Novel loci for childhood body mass index and shared heritability with adult cardiometabolic traits
Source: PLoS Genet. 2020 Oct 12;16(10):e1008718. doi: 10.1371/journal.pgen.1008718 (PMC7581004; doi:10.1371/journal.pgen.1008718)
Supplement: S6 Table — (DOCX) [file pgen.1008718.s006.docx]

**S6 Table** Results for the 25 SNPs with *P*-values <5 x 10^-8^ in the combined analysis, restricted to children aged ≥ 6 years

| **SNP** | **CHR** | **Position** | **Nearest gene** | **EA/non_EA** | **EAF^a^** | **Beta** | **SE** | **Sample size** | **P-value** |
| --- | --- | --- | --- | --- | --- | --- | --- | --- | --- |
| rs11676272^b,c^ | 2 | 25141538 | *ADCY3* | G/A | 0.47 | 0.072 | 0.006 | 53971 | **1.11 x 10^-28^** |
| rs7138803^b,c^ | 12 | 50247468 | *BCDIN3D* | A/G | 0.38 | 0.073 | 0.007 | 53374 | **1.22 x 10^-28^** |
| rs939584^b,c,d^ | 2 | 621558 | *TMEM18* | T/C | 0.83 | 0.093 | 0.009 | 55354 | **3.95 x 10^-28^** |
| rs17817449^b,c^ | 16 | 53813367 | *FTO* | G/T | 0.40 | 0.073 | 0.007 | 53270 | **4.09 x 10^-28^** |
| rs12042908^b,c^ | 1 | 74997762 | *FPGT-TNNI3K, TNNI3K* | A/G | 0.45 | 0.060 | 0.006 | 54429 | **5.12 x 10^-21^** |
| rs543874^b,c^ | 1 | 177889480 | *SEC16B* | G/A | 0.20 | 0.077 | 0.008 | 53270 | **2.92 x 10-^21^** |
| rs56133711^b^ | 11 | 27723334 | *BDNF* | A/G | 0.25 | 0.056 | 0.007 | 53896 | **2.36 x 10^-14^** |
| rs2076308^b,c^ | 6 | 50791640 | *TFAP2B* | C/G | 0.19 | 0.058 | 0.008 | 54429 | **1.34 x 10^-12^** |
| rs4477562^b,c,e^ | 13 | 54104968 | *LINC00558* | T/C | 0.13 | 0.069 | 0.009 | 55354 | **1.70 x 10^-13^** |
| rs571312^b,c^ | 18 | 57839769 | *MC4R* | A/C | 0.23 | 0.049 | 0.008 | 53375 | **1.26 x 10^-10^** |
| rs12641981^b,c^ | 4 | 45179883 | *GNPDA2* | T/C | 0.43 | 0.047 | 0.007 | 52076 | **1.46 x 10^-12^** |
| rs62107261^f^ | 2 | 422144 | *FAM150B* | T/C | 0.95 | 0.121 | 0.019 | 51810 | **8.51 x 10^-11^** |
| rs114285994^b^ | 16 | 19935763 | *GPRC5B* | G/A | 0.88 | 0.060 | 0.010 | 55354 | **3.53 x 10^-10^** |
| rs144376234^b,c^ | 1 | 110114504 | *GNAI3* | T/C | 0.04 | 0.112 | 0.018 | 55354 | **4.29 x 10^-10^** |
| rs1094647 | 1 | 205655378 | *SLC45A3* | G/A | 0.55 | 0.037 | 0.006 | 55354 | **5.69 x 10^-9^** |
| rs76227980^f^ | 18 | 58036384 | *MC4R* | C/T | 0.98 | 0.146 | 0.024 | 50055 | **6.61 x 10^-10^** |
| rs13107325^b^ | 4 | 103188709 | *SLC39A8* | T/C | 0.07 | 0.099 | 0.014 | 52413 | **5.67** **x 10^-12^** |
| rs62500888^c^ | 8 | 28061823 | *ELP3* | A/G | 0.57 | 0.037 | 0.006 | 55354 | **1.74 x 10^-9^** |
| rs114670539^c^ | 2 | 207064335 | *GPR1* | T/C | 0.05 | 0.088 | 0.015 | 55354 | **8.88 x 10^-9^** |
| rs61765651^b^ | 1 | 72754314 | *NEGR1* | C/T | 0.83 | 0.050 | 0.008 | 54429 | **2.33 x 10^-9^** |
| rs7719067^b^ | 5 | 153538241 | *GALNT10* | A/G | 0.42 | 0.037 | 0.006 | 53816 | **1.08 x 10^-8^** |
| rs11030391^f^ | 11 | 28644626 | *METTL15* | A/G | 0.63 | 0.035 | 0.007 | 55354 | 8.85 x 10^-8^ |
| rs184566112 | 18 | 55943926 | *NEDD4L* | A/T | 0.84 | 0.057 | 0.011 | 39459 | 1.77 x 10^-7^ |
| rs116664060 | 6 | 31592524 | *PRRC2A* | C/G | 0.19 | 0.052 | 0.009 | 44948 | 3.43 x 10^-8^ |
| rs11215427^b^ | 11 | 115093438 | *CADM1* | G/C | 0.73 | 0.039 | 0.007 | 50937 | **9.06 x 10^-8^** |

CHR, chromosome; EA, effect allele; EAF, effect allele frequency; SE, standard error.
Bolded P-values indicate genome-wide significance.
^a^ From combined analysis
^b^ Locus previously reported for adult BMI

^c^ Locus previously reported for childhood BMI

^d^ Locus previously reported for adult body fat
^e^ Locus previously reported for childhood obesity
^f^ Independent SNP at the same locus selected by conditional analysis

Discovery studies included in this analysis are: ALSPAC, BMDCS, CHOP, COPSAC2000, COPSAC2010, 1958BC-T1DGC, French Young Study- cases, French Young study- controls, GINIplus&LISA, GOYA male, TDCOB controls, MoBa, NFBC1966, NFBC1986, HBCS, NTR, PANIC, Raine Study, STRIP, TEENAGE, 1958BC-WTCCC, MoBa, INMA Sabadell and Valencia, BREATHE, DNBC-PTB, INMA Menorca, YFS, MAAS, Generation R. Replication studies included in this analysis are: Trails, TEDS OEE, TEDS AFFY, TDCOB controls, TDCOB cases, SKOT1, DNBC-GOYA offspring from obese mothers, DNBC-GOYA offspring from randomly selected mothers, Leipzig, EFSOCH, ABCD, CHOP, the FAMILY study, EDEN, PIAMA, SCOOP, MoBa, INMA Gipuzkoa.
