# Supplementary material for: Novel loci for childhood body mass index and shared heritability with adult cardiometabolic traits
Source: PLoS Genet. 2020 Oct 12;16(10):e1008718. doi: 10.1371/journal.pgen.1008718 (PMC7581004; doi:10.1371/journal.pgen.1008718)
Supplement: S7 Table — (DOCX) [file pgen.1008718.s007.docx]

| **Supplementary Table S7.** Results of the combined analyses for the 25 SNPs excluding case-control studies and excluding studies with a sample size smaller than n= 500 | | | | | | | | | | | | | | | | |
| --- | --- | --- | --- | --- | --- | --- | --- | --- | --- | --- | --- | --- | --- | --- | --- | --- |
| **SNP** | **CHR** | **Position** | **Nearest gene** | **EA/non_EA** | **Beta^a^** | **SE^a^** | **P-value^a^** | **N^a^** | **Beta^b^** | **SE^b^** | **P-value^b^** | **N^b^** | **Beta^c^** | **SE^c^** | **P-value^c^** | **N^c^** |
| rs11676272 | 2 | 25141538 | *ADCY3* | G/A | 0.071 | 0.006 | **3.79 x 10^-30^** | 57383 | 0.071 | 0.007 | **9.66 x 10^-28^** | 53813 | 0.070 | 0.006 | **1.22 x 10^-26^** | 53292 |
| rs7138803 | 12 | 50247468 | *BCDIN3D* | A/G | 0.072 | 0.006 | **4.23 x 10^-30^** | 58556 | 0.071 | 0.007 | **2.78 x 10^-27^** | 55145 | 0.073 | 0.007 | **1.32 x 10^-28^** | 54324 |
| rs939584 | 2 | 621558 | *TMEM18* | T/C | 0.092 | 0.008 | **3.73 x 10^-29^** | 59337 | 0.091 | 0.009 | **1.40 x 10^-26^** | 55767 | 0.089 | 0.009 | **1.24 x 10^-25^** | 54919 |
| rs17817449 | 16 | 53813367 | *FTO* | G/T | 0.069 | 0.006 | **2.98 x 10^-27^** | 57632 | 0.071 | 0.007 | **1.58 x 10^-26^** | 54220 | 0.067 | 0.007 | **8.83 x 10^-24^** | 53399 |
| rs12042908 | 1 | 74997762 | *FPGT-TNNI3K, TNNI3K* | A/G | 0.064 | 0.006 | **6.37 x 10^-25^** | 59362 | 0.063 | 0.006 | **1.67 x 10^-23^** | 55792 | 0.064 | 0.006 | **1.54 x 10^-23^** | 55018 |
| rs543874 | 1 | 177889480 | *SEC16B* | G/A | 0.075 | 0.008 | **6.02 x 10^-22^** | 57632 | 0.077 | 0.008 | **2.90 x 10^-21^** | 54220 | 0.071 | 0.008 | **3.83 x 10^-18^** | 53399 |
| rs56133711 | 11 | 27723334 | *BDNF* | A/G | 0.056 | 0.007 | **3.75 x 10^-15^** | 58829 | 0.057 | 0.007 | **8.66 x 10^-15^** | 55259 | 0.057 | 0.007 | **1.47 x 10^-14^** | 54111 |
| rs2076308 | 6 | 50791640 | *TFAP2B* | C/G | 0.058 | 0.008 | **3.07 x 10^-13^** | 59362 | 0.058 | 0.008 | **1.45 x 10^-12^** | 55792 | 0.056 | 0.008 | **1.19 x 10^-11^** | 55018 |
| rs4477562 | 13 | 54104968 | *LINC00558* | T/C | 0.065 | 0.009 | **5.81 x 10^-13^** | 60287 | 0.063 | 0.009 | **9.78 x 10^-12^** | 56717 | 0.063 | 0.009 | **1.65 x 10^-11^** | 55569 |
| rs571312 | 18 | 57839769 | *MC4R* | A/C | 0.052 | 0.007 | **8.80 x 10^-13^** | 57737 | 0.054 | 0.008 | **9.97 x 10^-13^** | 54325 | 0.053 | 0.008 | **3.83 x 10^-12^** | 53504 |
| rs12641981 | 4 | 45179883 | *GNPDA2* | T/C | 0.045 | 0.006 | **1.29 x 10^-12^** | 55488 | 0.044 | 0.007 | **2.90 x 10^-11^** | 51918 | 0.044 | 0.007 | **3.72 x 10^-11^** | 51397 |
| rs62107261 | 2 | 422144 | *FAM150B* | T/C | 0.121 | 0.018 | **9.93 x 10^-12^** | 56743 | 0.116 | 0.018 | **2.06 x 10^-10^** | 53173 | 0.116 | 0.018 | **3.65 x 10^-10^** | 52344 |
| rs114285994 | 16 | 19935763 | *GPRC5B* | G/A | 0.063 | 0.009 | **1.11 x 10^-11^** | 59337 | 0.062 | 0.010 | **8.62 x 10^-11^** | 55767 | 0.065 | 0.010 | **1.87 x 10^-11^** | 54919 |
| rs144376234 | 1 | 110114504 | *GNAI3* | T/C | 0.111 | 0.017 | **1.38 x 10^-10^** | 60287 | 0.105 | 0.018 | **3.71 x 10^-9^** | 56717 | 0.110 | 0.018 | **1.14 x 10^-9^** | 55569 |
| rs1094647 | 1 | 205655378 | *SLC45A3* | G/A | 0.038 | 0.006 | **7.20 x 10^-10^** | 60287 | 0.036 | 0.006 | **1.64 x 10^-8^** | 56717 | 0.038 | 0.006 | **3.34 x 10^-9^** | 55569 |
| rs76227980 | 18 | 58036384 | *MC4R* | C/T | 0.140 | 0.023 | **8.68 x 10^-10^** | 53353 | 0.148 | 0.024 | **3.21 x 10^-10^** | 50468 | 0.143 | 0.024 | **1.56 x 10^-9^** | 49229 |
| rs13107325 | 4 | 103188709 | *SLC39A8* | T/C | 0.082 | 0.014 | **1.38 x 10^-9^** | 58681 | 0.081 | 0.014 | **6.84 x 10^-9^** | 54755 | 0.085 | 0.014 | **1.91 x 10^-9^** | 53981 |
| rs62500888 | 8 | 28061823 | *ELP3* | A/G | 0.037 | 0.006 | **1.81 x 10^-9^** | 60287 | 0.038 | 0.006 | **4.20 x 10^-9^** | 56717 | 0.037 | 0.006 | **5.15 x 10^-9^** | 55569 |
| rs114670539 | 2 | 207064335 | *GPR1* | T/C | 0.088 | 0.015 | **1.92 x 10^-9^** | 59744 | 0.089 | 0.015 | **5.79 x 10^-9^** | 56174 | 0.092 | 0.015 | **1.64 x 10^-9^** | 55026 |
| rs61765651 | 1 | 72754314 | *NEGR1* | C/T | 0.047 | 0.008 | **4.99 x 10^-9^** | 59362 | 0.049 | 0.008 | **6.14 x 10^-9^** | 55792 | 0.047 | 0.008 | **1.58 x 10^-8^** | 55018 |
| rs7719067 | 5 | 153538241 | *GALNT10* | A/G | 0.036 | 0.006 | **6.54 x 10^-9^** | 58513 | 0.036 | 0.006 | **1.01 x 10^-8^** | 55696 | 0.033 | 0.006 | 1.51 x 10^-7^ | 55672 |
| rs11030391 | 11 | 28644626 | *METTL15* | A/G | 0.036 | 0.006 | **1.51 x 10^-8^** | 60287 | 0.038 | 0.007 | **9.57 x 10^-9^** | 56717 | 0.036 | 0.007 | **4.98 x 10^-8^** | 55569 |
| rs184566112 | 18 | 55943926 | *NEDD4L* | A/T | 0.057 | 0.011 | **4.24 x 10^-8^** | 42622 | 0.054 | 0.011 | 6.87 x 10^-7^ | 39737 | 0.053 | 0.011 | 1.19 x 10^-6^ | 38567 |
| rs116664060 | 6 | 31592524 | *PRRC2A* | C/G | 0.049 | 0.009 | **4.63 x 10^-8^** | 48931 | 0.044 | 0.009 | 2.72 x 10^-6^ | 45361 | 0.048 | 0.009 | 4.31 x 10^-7^ | 44513 |
| rs11215427 | 11 | 115093438 | *CADM1* | G/C | 0.039 | 0.007 | **4.64 x 10^-8^** | 55634 | 0.040 | 0.007 | **4.34 x 10^-8^** | 52817 | 0.037 | 0.007 | 2.84 x 10^-7^ | 52793 |

CHR, chromosome; EA, effect allele; EAF, effect allele frequency; SE, standard error.
Bolded P-values indicate genome-wide significance in the combined analysis.
^a^ From combined analysis, including all studies

^b^ From combined analysis, excluding case-control studies
^c^ From combined analysis, excluding studies with a sample size < n= 500
