# Supplementary material for: Novel loci for childhood body mass index and shared heritability with adult cardiometabolic traits
Source: PLoS Genet. 2020 Oct 12;16(10):e1008718. doi: 10.1371/journal.pgen.1008718 (PMC7581004; doi:10.1371/journal.pgen.1008718)
Supplement: S11 Table — (DOCX) [file pgen.1008718.s011.docx]

**S11 Table** Summary results of LD score regression analyses between childhood BMI and various metabolic, cardiovascular and lifestyle-related phenotypes

| **Phenotype 1** | **Phenotype 2** | **Genetic correlation (R_g_)** | **SE** | **Z** | **P-value** | **Reference of**  **phenotype 2 (PMID)** |
| --- | --- | --- | --- | --- | --- | --- |
| Childhood BMI | Infant head circumference | 0.32 | 0.08 | 3.90 | **9.68 x 10^-5^** | 22504419 |
| Childhood BMI | Birth Length | -0.04 | 0.07 | -0.55 | 0.58 | 25281659 |
| Childhood BMI | Adult height | -0.10 | 0.04 | -2.33 | **0.02** | 20881960 |
| Childhood BMI | Birth weight | 0.18 | 0.04 | 4.55 | **5.27 x 10^-6^** | 27680694 |
| Childhood BMI | Own birth weight (fetal effect), adjusted for maternal genotype | 0.20 | 0.05 | 41.59 | **3.19 x 10^-5^** | 31043758 |
| Childhood BMI | Offspring birth weight (maternal effect), adjusted for offspring genotype | 0.12 | 0.04 | 3.11 | **0.002** | 31043758 |
| Childhood BMI | Adult BMI | 0.76 | 0.03 | 22.55 | **1.45 x 10^-112^** | 20935630 |
| Childhood BMI | Adult overweight | 0.77 | 0.04 | 20.81 | **3.62 x 10^-96^** | 23563607 |
| Childhood BMI | Adult extreme BMI | 0.73 | 0.05 | 13.98 | **2.00 x 10^-44^** | 23563607 |
| Childhood BMI | Adult obesity class 1 | 0.71 | 0.04 | 19.69 | **2.65 x 10^-86^** | 23563607 |
| Childhood BMI | Adult obesity class 2 | 0.71 | 0.07 | 9.96 | **7.65 x 10^-61^** | 23563607 |
| Childhood BMI | Adult obesity class 3 | 0.69 | 0.01 | 16.46 | **2.35 x 10^-23^** | 23563607 |
| Childhood BMI | Weight | 0.53 | 0.03 | 17.25 | **1.16 x 10^-66^** | Data from UK Biobank |
| Childhood BMI | Waist circumference | 0.57 | 0.04 | 15.93 | **3.99 x 10^-57^** | 25673412 |
| Childhood BMI | Hip circumference | 0.53 | 0.04 | 14.06 | **6.38 x 10^-45^** | 25673412 |
| Childhood BMI | Waist-to-hip ratio | 0.39 | 0.04 | 9.29 | **1.57 x 10^-20^** | 25673412 |
| Childhood BMI | Body fat percentage | 0.46 | 0.03 | 13.88 | **7.99 x 10^-44^** | Data from UK Biobank |
| Childhood BMI | Age at menarche | -0.42 | 0.04 | -119.12 | **1.03 x 10^-32^** | 25231870 |
| Childhood BMI | Age at menopause | -0.07 | 0.05 | -14.38 | 0.15 | 26414677 |
| Childhood BMI | Diastolic blood pressure | 0.11 | 0.04 | 3.10 | **0.002** | Data from UK Biobank |
| Childhood BMI | Systolic blood pressure | 0.02 | 0.04 | 0.60 | 0.55 | Data from UK Biobank |
| Childhood BMI | Total cholesterol | -0.15 | 0.05 | -3.27 | **0.001** | 20686565 |
| Childhood BMI | Triglycerides | 0.50 | 0.04 | 1.18 | 0.24 | 20686565 |
| Childhood BMI | Low-density lipoprotein | -0.10 | 0.05 | -1.83 | 0.07 | 20686565 |
| Childhood BMI | High-density lipoprotein | -0.22 | 0.05 | -4.45 | **8.65 x 10^-6^** | 20686565 |
| Childhood BMI | Type 2 diabetes | 0.19 | 0.06 | 3.08 | **0.002** | 22885922 |
| Childhood BMI | Fasting glucose | 0.07 | 0.06 | 1.24 | 0.22 | 22581228 |
| Childhood BMI | Fasting insulin | 0.34 | 0.07 | 4.64 | **3.50 x 10^-6^** | 22581228 |
| Childhood BMI | HOMA-B | 0.20 | 0.08 | 2.56 | 0.01 | 20081858 |
| Childhood BMI | HOMA-IR | 0.28 | 0.09 | 3.01 | **0.003** | 20081858 |
| Childhood BMI | HbA1c | 0.09 | 0.08 | 1.20 | 0.23 | 20858683 |
| Childhood BMI | Coronary artery disease | 0.14 | 0.04 | 3.31 | **0.001** | 26343387 |
| Childhood BMI | Childhood IQ | -0.05 | 0.08 | -0.58 | 0.56 | 23358156 |
| Childhood BMI | Years of schooling | -0.10 | 0.03 | -3.16 | **0.002** | 27225129 |
| Childhood BMI | Crohns disease | 0.10 | 0.05 | 2.09 | **0.04** | 26192919 |
| Childhood BMI | Asthma | -0.04 | 0.07 | -0.48 | 0.63 | 17611496 |
| Childhood BMI | Rheumatoid Arthritis | 0.05 | 0.05 | 0.93 | 0.35 | 24390342 |
| Childhood BMI | Ulcerative colitis | -0.10 | 0.05 | -1.78 | 0.08 | 26192919 |
| Childhood BMI | Inflammatory bowel diseases | -0.01 | 0.05 | -0.15 | 0.88 | 26192919 |
| Childhood BMI | Celiac disease | 0.02 | 0.08 | 0.29 | 0.77 | 20190752 |
| Childhood BMI | Alzheimers disease | -0.13 | 0.09 | -1.45 | 0.15 | 24162737 |
| Childhood BMI | Parkinsons disease | -0.03 | 0.06 | -0.43 | 0.67 | 19915575 |
| Childhood BMI | Cigarettes per day | 0.26 | 0.09 | 2.78 | **0.01** | 20418890 |
| Childhood BMI | Ever vs never smoked | 0.16 | 0.06 | 2.53 | **0.01** | 20418890 |
| Childhood BMI | Age of smoking initation | -0.07 | 0.11 | -0.65 | 0.52 | 20418890 |
| Childhood BMI | Former vs current smoker | -0.11 | 0.08 | -1.28 | 0.20 | 20418890 |
| Childhood BMI | Sleep duration | -0.01 | 0.05 | -0.22 | 0.82 | 27494321 |
| Childhood BMI | Insomnia | -0.04 | 0.06 | -0.63 | 0.53 | 28604731 |
| Childhood BMI | Chronotype | 0.08 | 0.04 | 1.72 | 0.09 | 27494321 |
| Childhood BMI | Leptin | 0.34 | 0.09 | 3.95 | **7.85 x 10^-5^** | 26833098 |
| Childhood BMI | Leptin adjusted for BMI | -0.12 | 0.08 | -1.58 | 0.11 | 26833098 |
| Childhood BMI | Forced vital capacity (FVC) | -0.03 | 0.04 | -0.72 | 0.47 | 28166213 |
| Childhood BMI | Forced expiratory volume in 1 sec. (FEV1) | -0.04 | 0.04 | -1.07 | 0.28 | 28166213 |
| Childhood BMI | FEV1/FVC | -0.04 | 0.05 | -0.78 | 0.44 | 28166213 |
| Childhood BMI | Intracranial volume | 0.22 | 0.10 | 2.18 | **0.03** | 25607358 |

SE: Standard Error
