# Supplementary material for: Novel loci for childhood body mass index and shared heritability with adult cardiometabolic traits
Source: PLoS Genet. 2020 Oct 12;16(10):e1008718. doi: 10.1371/journal.pgen.1008718 (PMC7581004; doi:10.1371/journal.pgen.1008718)
Supplement: S12 Table — (DOCX) [file pgen.1008718.s012.docx]

**S12 Table** Results of adult BMI GWAS meta-analysis for 20 loci with *P-*values < 5 x 10^-8^ in the combined analysis

| **SNP** | **Proxy** | **CHR** | **Position** | **Nearest gene** | **EA/non_EA** | **EAF^a^** | **Beta^a^** | **SE^a^** | **P-value ^a^** | **N^a^** |
| --- | --- | --- | --- | --- | --- | --- | --- | --- | --- | --- |
| rs11676272^b,c^ | - | 2 | 25141538 | *ADCY3* | G/A | 0.48 | 0.033 | 0.002 | **8.0 x 10^-86^** | 740116 |
| rs7138803^b,c^ | - | 12 | 50247468 | *BCDIN3D* | A/G | 0.38 | 0.030 | 0.002 | **2.3 x 10^-71^** | 795588 |
| rs939584^b,c,d^ | - | 2 | 621558 | *TMEM18* | T/C | 0.83 | 0.054 | 0.004 | **5.3 x 10^-129^** | 691960 |
| rs17817449^b,c^ | - | 16 | 53813367 | *FTO* | G/T | 0.39 | 0.075 | 0.002 | **0.0 x 10^-0^** | 795552 |
| rs12042908^b,c^ | - | 1 | 74997762 | *FPGT-TNNI3K, TNNI3K* | A/G | 0.46 | 0.018 | 0.002 | **1.4 x 10^-29^** | 794074 |
| rs543874^b,c^ | - | 1 | 177889480 | *SEC16B* | G/A | 0.20 | 0.048 | 0.004 | **1.2 x 10^-122^** | 795504 |
| rs56133711^b^ | rs10835211 (r^2^= 0.87) | 11 | 27723334 | *BDNF* | A/G | 0.26 | 0.029 | 0.002 | **2.0 x 10^-54^** | 795539 |
| rs2076308^b,c^ | rs987237 (r2= 1) | 6 | 50791640 | *TFAP2B* | C/G | 0.28 | 0.041 | 0.0002 | **9.3 x 10^-84^** | 795612 |
| rs4477562^b,c,e^ | - | 13 | 54104968 | *LINC00558* | T/C | 0.12 | 0.032 | 0.003 | **4.6 x 10^-34^** | 685151 |
| rs571312^b,c^ | - | 18 | 57839769 | *MC4R* | A/C | 0.23 | 0.054 | 0.002 | **1.8 x 10^-176^** | 795371 |
| rs12641981^b,c^ | - | 4 | 45179883 | *GNPDA2* | T/C | 0.43 | 0.032 | 0.002 | **8.0 x 10^-86^** | 793326 |
| rs62107261^f^ | Not available | 2 | 422144 | *FAM150B* | T/C | - | - | - | **-** | **-** |
| rs114285994^b^ | rs8055543 (r2= 0.89) | 16 | 19935763 | *GPRC5B* | G/A | 0.86 | 0.033 | 0.003 | **5.0 x 10^-37^** | 563115 |
| rs144376234^b,c^ | rs7550711 (r^2^= 1) | 1 | 110114504 | *GNAI3* | T/C | 0.03 | 0.065 | 0.005 | **3.0 x 10^-38^** | 769184 |
| rs1094647 | rs823105 (r^2^= 0.96) | 1 | 205655378 | *SLC45A3* | G/A | 0.55 | 0.007 | 0.002 | 2.7 x 10^-5^ | 686054 |
| rs76227980^f^ | rs2229616 (r^2^= 1) | 18 | 58036384 | *MC4R* | C/T | 0.98 | 0.106 | 0.006 | **4.7 x 10^-71^** | 760183 |
| rs13107325^b^ | - | 4 | 103188709 | *SLC39A8* | T/C | 0.07 | 0.047 | 0.003 | **1.1 x 10^-47^** | 792045 |
| rs62500888^c^ | rs7821358 (r^2^= 1) | 8 | 28061823 | *ELP3* | A/G | 0.55 | 0.010 | 0.002 | **2.7 x 10^-8^** | 691190 |
| rs114670539^c^ | Not available | 2 | 207064335 | *GPR1* | T/C | - | - | - | **-** | **-** |
| rs61765651^b^ | Not available | 1 | 72754314 | *NEGR1* | C/T | - | - | - | **-** | **-** |
| rs7719067^b^ | - | 5 | 153538241 | *GALNT10* | A/G | 0.42 | 0.017 | 0.002 | **3.1 x 10^-23^** | 692582 |
| rs11030391^f^ | rs4434991 (r^2^= 0.96) | 11 | 28644626 | *METTL15* | A/G | 0.62 | 0.008 | 0.002 | 2.1 x 10^-6^ | 691283 |
| rs184566112 | Not available | 18 | 55943926 | *NEDD4L* | A/T | - | - | - | **-** | **-** |
| rs116664060 | Not available | 6 | 31592524 | *PRRC2A* | C/G | - | - | - | **-** | **-** |
| rs11215427^b^ | - | 11 | 115093438 | *CADM1* | G/C | 0.83 | 0.012 | 0.002 | **6.3 x 10^-10^** | 687136 |

^a^ From adult BMI analysis

^b^ Locus previously reported for adult BMI

^c^ Locus previously reported for childhood BMI

^d^ Locus previously reported for adult body fat
^e^ Locus previously reported for childhood obesity
^f^ Independent SNP at the same locus selected by conditional analysis

CHR: Chromosome; EA: Effect Allele; EAF: Effect Allele Frequency; SE: Standard Error

P*-*values < 5 x 10^-8^ are considered statistically significant.
