# Supplementary material for: Novel loci for childhood body mass index and shared heritability with adult cardiometabolic traits
Source: PLoS Genet. 2020 Oct 12;16(10):e1008718. doi: 10.1371/journal.pgen.1008718 (PMC7581004; doi:10.1371/journal.pgen.1008718)
Supplement: S13 Table — (DOCX) [file pgen.1008718.s013.docx]

**S13 Table** Results of the GRS for birth weight and childhood cardio-metabolic phenotypes

|  | **SDS Birth weight** | **Childhood insulin^a^** | **Childhood triglycerides^a^** | **Childhood LDL cholesterol^a^** | **Childhood HDL cholesterol^a^** | **Childhood total cholesterol^a^** | **Childhood diastolic blood pressure^a^** | **Childhood systolic blood pressure^a^** |
| --- | --- | --- | --- | --- | --- | --- | --- | --- |
| **BMI GRS** | 0,015 (0.008) | 0.066 (0.039) | 0.010 (0.005) | 0.007 (0.006) | -0.008 (0.003) | 0,004 (0.006) | 0.051 (0.058) | 0,169 (0.068) |

^a^Regression coefficients (SE) are linear regression coefficients adjusted for age and sex

P*-*values < 0.00625 are considered statistically significant.
