# Supplementary figures and images for: Novel loci for childhood body mass index and shared heritability with adult cardiometabolic traits
Source: PLoS Genet. 2020 Oct 12;16(10):e1008718. doi: 10.1371/journal.pgen.1008718 (PMC7581004; doi:10.1371/journal.pgen.1008718)

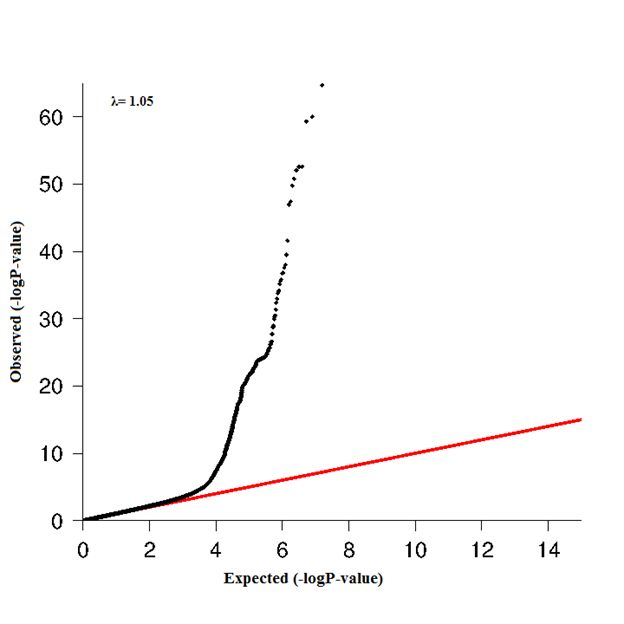

Supplement: S1 Fig — (TIF) [file pgen.1008718.s014.tif]

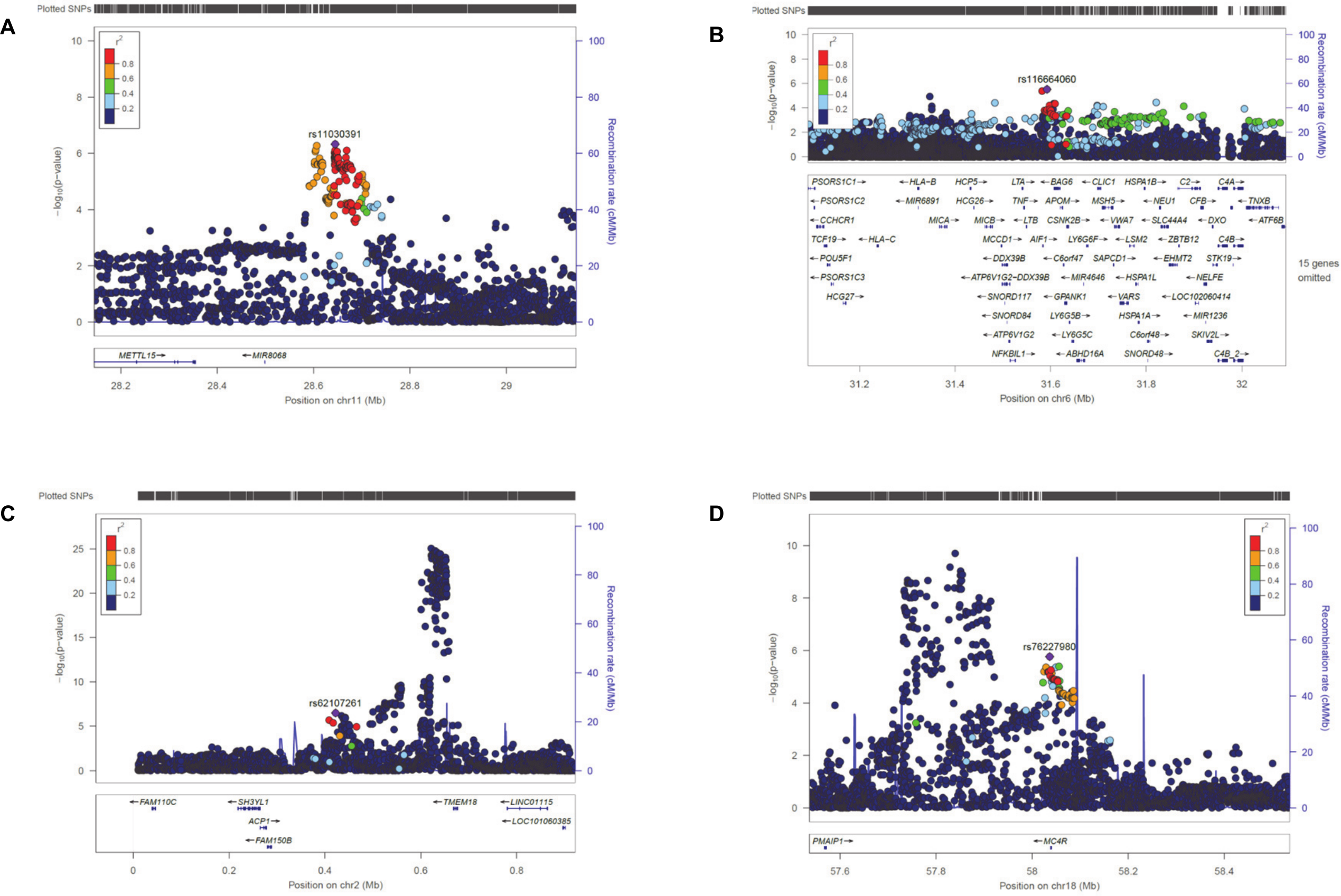

Supplement: S2 Fig — (TIF) [file pgen.1008718.s015.tif]
