# Supplementary material for: Novel loci for childhood body mass index and shared heritability with adult cardiometabolic traits
Source: PLoS Genet. 2020 Oct 12;16(10):e1008718. doi: 10.1371/journal.pgen.1008718 (PMC7581004; doi:10.1371/journal.pgen.1008718)
Supplement: S1 Text — (DOCX) [file pgen.1008718.s016.docx]

**S1 Text** *Cohort information***ABCD study**The Amsterdam Born Children and their Development (ABCD) cohort study is a large, community-based birth cohort, which started in 2003 with the inclusion of 8,000 pregnant women living in Amsterdam and its main aim is to study factors in early life (during pregnancy and infancy) that explain health later in life. Detailed information on life style habits, psychosocial determinants, obstetric complications, blood pressure course during pregnancy, as well as childhood growth patterns has been gathered. Data for this study comes from ABCD-Genetic Enrichment (ABCD-GE) study, a sub-study of 1192 ethnic Dutch children. The blood was collected from a simple finger prick during the 5-year health check-up of the children (2008-2010). DNA was extracted from the dried blood spots. The ABCD study protocol was approved by the Central Committee on Research Involving Human Subjects in The Netherlands, the medical ethics review committees of the participating hospitals and the Registration Committee of the Municipality of Amsterdam. All ABCD participants gave written informed consent for data collection of the phenotypes. Regarding the DNA collection and analysis, an opt-out procedure was used (METC approval 2002_039#B2013531).

**Avon Longitudinal Study of Parents and Children (ALSPAC)**

Pregnant women resident in Avon, UK with expected dates of delivery 1st April 1991 to 31st December 1992 were invited to take part in the study[1,2]. The initial number of pregnancies enrolled is 14,541 (for these at least one questionnaire has been returned or a “Children in Focus” clinic had been attended by 19/07/99). Of these initial pregnancies, there was a total of 14,676 foetuses, resulting in 14,062 live births and 13,988 children who were alive at 1 year of age.

When the oldest children were approximately 7 years of age, an attempt was made to bolster the initial sample with eligible cases who had failed to join the study originally. As a result, when considering variables collected from the age of seven onwards (and potentially abstracted from obstetric notes) there are data available for more than the 14,541 pregnancies mentioned above. The number of new pregnancies not in the initial sample (known as Phase I enrolment) that are currently represented on the built files and reflecting enrolment status at the age of 24 is 913 (456, 262 and 195 recruited during Phases II, III and IV respectively), resulting in an additional 913 children being enrolled. The phases of enrolment are described in more detail in the cohort profile paper and its update (see footnote 4 below). The total sample size for analyses using any data collected after the age of seven is therefore 15,454 pregnancies, resulting in 15,589 foetuses. Of these 14,901 were alive at 1 year of age.

A 10% sample of the ALSPAC cohort, known as the Children in Focus (CiF) group, attended clinics at the University of Bristol at various time intervals between 4 to 61 months of age. The CiF group were chosen at random from the last 6 months of ALSPAC births (1432 families attended at least one clinic). Excluded were those mothers who had moved out of the area or were lost to follow-up, and those partaking in another study of infant development in Avon.

Please note that the study website contains details of all the data that is available through a fully searchable data dictionary and variable search tool: http://www.bristol.ac.uk/alspac/researchers/our-data/

Ethical approval was obtained from the ALSPAC Ethics and Law Committee and the Local Research Ethics Committees.

*ALSPAC GWAS data*

A total of 9,912 participants were genotyped using the Illumina HumanHap550 quad genome-wide SNP genotyping platform by the Wellcome Trust Sanger Institute, Cambridge, UK and the Laboratory Corporation of America, Burlington, NC, USA using support from 23andMe. Details on the QC procedure have been published previously[3]. Individuals were excluded from further analysis on the basis of having incorrect sex assignments; extreme heterozygosity (<0.320 and >0.345 for the Sanger data and <0.310 and >0.330 for the LabCorp data); high levels of individual missingness (>3%); evidence of cryptic relatedness (>10% IBD) and being of non-European ancestry (as detected by a multidimensional scaling analysis seeded with HapMap 2 individuals). EIGENSTRAT analysis revealed no additional obvious population stratification and genome-wide analyses with other phenotypes indicate a low lambda. The resulting data set consisted of 8,365 individuals. SNPs with a minor allele frequency of <1% and call rate of <95% were removed. Only SNPs which passed an exact test of Hardy–Weinberg equilibrium (HWE) (*P-*value  >5 × 10^-7^) were considered for analysis. Known autosomal variants were imputed with IMPUTE2, using 1000G V1 CEPH. After imputation, SNPs with a minor allele frequency <0.01 and an r^2^ imputation quality score <0.3 were excluded and this resulted in 18486880 SNPs available for analysis. Association analyses were performed using SNPTEST.

**BMDCS**

The multi-center, longitudinal Bone Mineral Density in Childhood Study enrolled children 5 to 19 years of age in five locations in the United States; Children’s Hospital of Los Angeles (Los Angeles, CA), Cincinnati Children’s Hospital Medical Center (Cincinnati, OH), Creighton University (Omaha, NE), Children’s Hospital of Philadelphia (Philadelphia, PA), and Columbia University (New York, NY). As described previously, females 6-15 years and males 6-16 years of age were enrolled in 2002-2003, and measured annually for six years (up to seven visits)[4,5]. Additional study participants, 5 and 19 years of age were enrolled in 2006-2007 and evaluated annually for two years (up to three visits). In 2010 and 2011, additional 507 children of European ethnic origin aged 5 to 18 year old were enrolled for a one-time visit in the Creighton and Cincinnati centers. Criteria for study entry targeted normal development and healthy bones. Key criteria included term birth (≥37 wk gestation); birth weight >2.3 kg; no evidence of precocious or delayed puberty, and height, weight or BMI within the 3rd to the 97th percentile for age. Exclusion criteria included multiple fractures (more than two fractures if age <10 years or more than three fractures if age >10 years), current or previous medication use or medical condition known to affect bone health, and extended bed rest. Opposite sex siblings were not excluded from study participation; for the purpose of this analysis the older sibling was excluded. A blood or saliva sample was collected at the final study visit. Written informed consent was obtained the parent or guardian for study participants less than 18 years of age and from the study participant if age 18 years and older. Assent was obtained from participants less than age 18 years. The protocol was approved by the Institutional Review Board of each Clinical Center.

Measurements were obtained by trained personnel with the research participant wearing light clothing with shoes and hair adornments removed. Height was measured with a stadiometer to the nearest 0.1cm and weight was measured with a digital electronic scale to the nearest 0.1kg.

**BRain dEvelopment and Air polluTion ultrafine particles in scHool childrEn Project (BREATHE)**

This is a population-based study that aims to investigate the impact of air pollution on the cognitive development of children. Children from second, third and fourth levels of 39 primary schools in Barcelona and surroundings were visited four times during 2012 and 2013. Children were born in 2001-2004 and were 7-11 years old during the visits. Informed consent was obtained from all participants and the study was approved by the Research Center Ethics Committee.

Weight and height were measured with calibrated scales and a portable stadiometer, respectively, with the children standing in light clothing and barefoot, by trained field workers. BMI was calculated as weight in kilograms divided by the square of height in meters. Sex- and age-adjusted standard deviation scores (SDS) were constructed using LMS growth (Pan H, Cole TJ, 2012. http://www.healthforallchildren.co.uk).

DNA samples from 2,492 BREATHE children were obtained from saliva collected in Oragene DNA OG‐500kit (DNA Genotek) following manufacturer’s instructions with minor modifications. All DNA samples were quantified using Quant-iT™ PicoGreen® dsDNA Assay Kit (Life Technologies). A subset of 1,778 children was selected for genome-wide genotyping. Genome-wide genotyping was performed using the HumanCore BeadChip WG-330-1101 (Illumina) at the Spanish National Genotyping Center (CEGEN) at the Spanish National Cancer Research Centre (CNIO). Genotype calling was done using the GeneTrain2.0 algorithm (with a default threshold of 0.15) based on HapMap clusters implemented in the GenomeStudio software. Twenty CEU HapMap duplicates and twenty BREATHE duplicates were included in the study and gave consistent results.

PLINK was used for the genetic data quality control. Five samples were initially excluded because they were filtered from the epidemiological database. We applied the following sample quality control thresholds: sample call rate>97% (N=3 exclusions) and heterozigosity 4 SD (N=5 exclusions). Then, we checked sex discordances (N=18 exclusions, 1%), relatedness (N=80 exclusions: one twin, 32 siblings, 39 cousins, eight incongruent sibling's couples). In total we excluded 106 subjects (6%). Genetic variants were filtered by SNP call rate>95%, MAF>1% and HWE *P*-value>1.10 x 10^-6^ (N=58,827 exclusions, 19.7%). The final genetic data set consisted of 1,633 subjects and 240,103 SNPs coded in b37 and + strand.  Imputation of genetic variants was done using IMPUTE V2 and the cosmopolitan 1000 genome panel (release March 2012).

**British 1958 Birth Cohort (1958BC-WTCCC and 1958BC-T1DGC)**
The 1958 British Birth Cohort (1958BC) consists of all born during one week in March 1958 in England, Scotland, and Wales (n=17,638)[6]. The cohort has been followed-up from birth with contact at ages 7, 11,  16, 23, 33, 42, 45, 46 and 50 years. Measurements of height and weight were obtained at all childhood contacts and in this study; information from 7 years was used to calculate BMI defining centiles from within the study. At age 45 years, 11,971 cohort members who had not died or emigrated, were invited to a biomedical assessment. In total 9,377 participants provided data, including several clinical assessments and DNA collection. Ethical approval for the 45y survey was obtained from South East Multi-centre Research Ethics Committee (ref. 01/1/44)  and the Joint UCL/UCLH Committees on the Ethics of Human Research (Committee A) Ref: 08/H0714/40.

Genome-wide data for the 1958BC has been obtained through two sub-studies, both using the 1958BC members as a control population. First, 3000 DNA samples were randomly selected as part of the Wellcome Trust Case Control Consortium (WTCCC2) and genotyped on the Affymetrix SNP 6.0 platform[7]. Samples were genotyped through the JDRF/WT Diabetes and Inflammation Laboratory (DIL) using the Illumina Infinium 550K chip[8]. Imputation was done in IMPUTE after quality control. For B58C-WTCCC2 quality control included SNP exclusions (Minor allele frequency (MAF) < 0.01, HWE *P*-value <1 x 10^-20^, call rate < 0.98, genotype plate association <1 x 10^-5^), and sample exclusions (heterozgygosity, call rate, relatedness, non-European ancestry and sex discrepancy). For B58C-T1DGC, criteria for SNP exclusions were MAF < 0.01, HWE *P*-value <1 x 10^-7^, or SNP call rate < 0.95, and sample exclusions were made for heterozygosity, call rate, non-European ancestry and potential sex discrepancy.

**Cardiovascular Risk in Young Finns Study (YFS)**

YFS is a large prospective population-based multicentre study aimed at examining the risk factors and their determinants in children and adolescents of risk factors for atherosclerosis[9]. The baseline cross-sectional study was carried out in 1980, including 3596 subjects at ages 3, 6, 9, 12, 15, and 18. Between 1980 and 1992, these subjects were followed up at 3-year intervals, and then as adults in 2001, 2007 and 2010-2012. The main aim of the YFS is to determine the contribution made by childhood lifestyle, biological and psychological measures to the risk of cardiovascular diseases in adulthood.

Genotyping was performed with the Illumina 670K SNP chip at the Wellcome Trust Sanger Centre. Quality control measures were taken prior to imputation (excluding genotypes with call rate <0.95, MAF <0.01, HWE *P*-value <1 x 10^-6^). Genotype imputation was performed using IMPUTE2[10,11] and a reference panel from the 1000 Genomes. All subjects gave their written informed consent in 2007 and the study was approved by local ethics committees of the participating universities.

**Children’s Hospital of Philadelphia (CHOP)**

All subjects were consecutively recruited from the Greater Philadelphia area from 2006 to 2014 at the Children's Hospital of Philadelphia. Our study cohort consisted of children of European ancestry. All of these participants had their blood drawn in to an 8ml EDTA blood collection tube and were subsequently DNA extracted for genotyping. All subjects were biologically unrelated and were aged between 2 and 18 years old. This study was approved by the Institutional Review Board of the Children's Hospital of Philadelphia. Parental informed consent was given for each study participant for both the blood collection and subsequent genotyping. BMI was determined from height and weight measurements in the clinical record.

We performed high throughput genome-wide SNP genotyping, using the Illumina Infinium™ II HumanHap550 BeadChip technology (Illumina, San Diego), at the Center for Applied Genomics at CHOP. We used 750ng of genomic DNA to genotype each sample, according to the manufacturer’s guidelines.

Samples were genotyped on a combination of the HumanHap 550 version 1, HumanHap 550 Version 3 and 610 Quad SNP chips. The 535,931 SNPs in common with the three different chip versions used were the basis for all further analyses. 1,878 SNPs were excluded for having a HWE *P*-value less than 1 x 10^-6^. 321 SNPs were thrown out for missing more than 5 percent of genotypes and 20,902 SNPs were thrown out for having a minor allele frequency less than 1 percent. Impute2 was used to impute ~37 million SNPs using the 1000 Genomes Project Phase 1 reference haplotypes.

**CHOP (Europe)**

The CHOP study (European Childhood Obesity Project) is an ongoing European multicenter randomized prospective nutritional intervention study in 1678 healthy term newborns recruited between October 1, 2002 and July 31, 2004. Currently, infants are followed up until the age of 11 years. Main objective of the CHOP study is to assess the effect of early and later nutrition on children’s weight development, growth, body composition and risk of obesity and the role of genetic variation and epigenetic and metabolic programming plays in this context. A detailed description of the study design and the comprehensive prospective measurements can be found in recent publications[12-16]. The local ethics committees of each study center approved all study procedures: Belgium (Comitè d’Ethique de L’Hopital Universitaire des Enfants Reine Fabiola; no. CEH 14/02), Germany (Bayerische Landesärztekammer Ethik-Kommission; no. 02070), Italy (Azienda Ospedaliera San Paolo Comitato Etico; no. 14/2002), Poland (Instytut Pomnik–Centrum Zdrowia Dziecka Komitet Etyczny; no 243/KE/2001), and Spain (Comité ético de investigación clinica del Hospital Universitario de Tarragona Joan XXIII). Written informed parental consent was obtained for each participating infant and from children of age 8 years onwards.

*Genotyping , Quality Control and Imputation*

For this genome-wide-association analysis (GWAS) genetic data on n=374 children were available from the CHOP study. Buffy coats were collected from children of age 5.5 years during physical exam. Samples were genotyped from buffy coats with Illumina HumanOmniExpress-24 v1.0 arrays by Dr Eva Reischl and team at the Genome Analysis Center of Helmholtz Zentrum Muenchen, Germany by standard procedures according to manufactures instructions (Illumina Inc., San Diego, USA).

Quality control on genotyped data were performed at Helmholtz Zentrum Muenchen and in even more detail by Dr Linda Broer and team at the Department of Internal Medicine, Genetic Laboratory, Erasmus Medical Center, Rotterdam, The Netherlands. QC tests using mainly PLINK comprised SNP missingness test (GENO>0.05), frequency test (MAF<0.001), HWE-test (p<=1 × 10^-7^), Sample call rate >95% and SNP call rate >80% and Sample call rate >97.5% and SNP call rate >95%, test for evidence of heterozygosity or excess homozygosity, test for gender mismatch, test for Caucasian samples and test for familiar relationships (IBD). From the originally n=382 samples with 701,281 SNPs 264 SNPS failed the HWE-test (p<=1 × 10^-7^) and 14,627 SNPs failed the MAF test (<0.1%) leaving 686,390 SNPs. For 8 samples there was evidence of excess heterogeneity (F-value < mean –(4×SD)) and these samples were removed leaving n=374 samples with 686,390 SNPs. No gender mismatches were identified. However, 10 of the 374 samples were identified as potentially non-European by IBS/IBD distance analysis in PLINK but kept in the data set. This issue was accounted for by including 10 pruned principal components from multidimensional scaling procedure in the GWAS analysis as adjustment factors (see below). Overall, quality controlled genotyped data from 374 children with 686,390 SNPs were available.

Imputation of these quality controlled genotyped data with reference to the 1000G reference panel (phase1_v3) were performed by Dr Linda Broer and team at the Department of Internal Medicine, Genetic Laboratory, Erasmus Medical Center, Rotterdam, The Netherlands. Imputation was done using a two steps procedure using MINIMAC3 and MACH for phasing and imputation respectively for the 374 children and by using the Michigan Imputation server (<https://imputationserver.sph.umich.edu/index.html>). The final number of SNPs with MAF>1% and r^2^ >0.3 after the imputation process is 9,642,852. MACH R² when excluding variants MAF < 1% is 0.88 (median = 0.97, SD = 0.20).

*Conducted analysis and description of covariates*

Statistical analysis is based on all genotyped and imputed data (9,642,852 SNPs over 22 chromosomes) for n=374 children. However due to missing data in phenotype (standardized BMI) analysis is based on n=369 children only. Weight and height were measured with calibrated scales and stadiometer, respectively, with the children standing in light clothing and barefoot, by trained field workers. BMI was calculated as weight in kilograms divided by the square of height in meters. Sex- and age-adjusted standard deviation scores (SDS) were constructed using LMS growth (Pan H, Cole TJ, 2012. [http://www.healthforallchildren.co.uk](http://www.healthforallchildren.co.uk/) ).

The following additive linear model described in the analysis plan was conducted by statistical software rvtest (<https://github.com/zhanxw/rvtests>[17] and accounting for imputation of SNPs by analysing dosage data and by adjusting for the 10 pruned principal components derived from MDS analysis of genotyped data (see QC above).

*BMI_SDS ~ SNP + 10 pruned principal MDS-components*

**Copenhagen Study on Asthma in Childhood 2000 birth cohort (COPSAC2000)**

The COPSAC2000 birth cohort study is a prospective clinical study of a birth cohort of 411 infants born to mothers with a history of asthma. The newborns were enrolled at the age of 1 month, the recruitment of which was previously described in detail[18]. The study was conducted in accordance with the guiding principles of the Declaration of Helsinki and was approved by the Local Ethics Committee (KF 01-289/96), and the Danish Data Protection Agency (2008-41-1754). Both parents gave written informed consent before enrolment.

The families used doctors employed at the clinical research unit, and not the family practitioner, for diagnosis and treatment of any respiratory or skin-related symptoms. Participants were assessed at the COPSAC clinical research unit at six monthly intervals; additional visits were arranged immediately upon the onset of symptoms. All growth parameters were measured and obtained by the COPSAC physicians at each scheduled six monthly visit until age 7 and history were obtained using structured questions and closed response categories. At every visit weight was measured using calibrated digital weight scales and length by infantometer, Kiddimetre® (Raven Equipment Limited, Essex England). From 2.5 years, height was measured using a stadiometer (Harpenden; Holtain Ltd, Crymych, Dyfed, Wales)[19].

High throughput genome-wide SNP genotyping were performed using the Illumina Infinium™ II High throughput genome-wide SNP genotyping were performed using the Illumina HumanOmniExpressExome BeadChip (Illumina, San Diego), at AROS Applied Biotechnology[20]. Imputation was performed using IMPUTE2 and 1000 genomes (phase I v3 ALL) as reference panel. Statistical analysis was carried out using R project, assuming an additive model and taking genotype uncertainty into account.

**Copenhagen Study on Asthma in Childhood 2010 birth cohort (COPSAC2010)**

The COPSAC2010 birth cohort study is a prospective clinical study of a 700 unselected newborns, previously described in detail[19]. The study was conducted in accordance with the guiding principles of the Declaration of Helsinki and was approved by the Local Ethics Committee (H-B-2008-093), and the Danish Data Protection Agency (2008-41-2599). Both parents gave written informed consent before enrolment.

The families used doctors employed at the clinical research unit, and not the family practitioner, for diagnosis and treatment of any respiratory or skin-related symptoms. Participants were assessed at the COPSAC clinical research unit at six monthly intervals; additional visits were arranged immediately upon the onset of symptoms. All growth parameters were measured and obtained by the COPSAC physicians at each scheduled six monthly visit until age 3 and history were obtained using structured questions and closed response categories. At every visit weight was measured using calibrated digital weight scales and length by infantometer, Kiddimetre® (Raven Equipment Limited, Essex England). From 2.5 years, height was measured using a stadiometer (Harpenden; Holtain Ltd, Crymych, Dyfed, Wales)[19].

High throughput genome-wide SNP genotyping were performed using the Illumina HumanOmniExpressExome BeadChip (Illumina, San Diego), at AROS Applied Biotechnlogy[20]. Imputation was performed using IMPUTE2 and 1000 genomes (phase I v3 ALL) as reference panel. Statistical analysis was carried out using R project, assuming an additive model and taking genotype uncertainty into account.

**Danish National Birth Cohort (DNBC) - GOYA offspring**

The Danish National Birth cohort (DNBC) was established in 1996-2002 enrolling a total of 100,417 pregnancies among 92,274 women from all over Denmark[21]. From this cohort, the nested genome-wide association study GOYA (Genomics of extremely Overweight young Adults) sampled and genotyped 1960 mothers with the highest BMI and 1948 randomly selected mothers[22]. The GOYA offspring sample consists of 407 children born to the obese mothers and 481 children born to the randomly selected mothers, all with available cord blood. A questionnaire follow-up was conducted when the children were 7 years old including self-reported information on the child’s weight and height.

The children were genotyped using the Illumina Infinium HumanCoreExome Beadchip (Illumina, San Diego, CA, USA) and genotypes were called using the Genotyping module, version 1.9.4 of GenomeStudio software, Version 2011.1 (Illumina). During genotype quality control, we excluded closely related individuals and samples with extreme inbreeding coefficients, mislabeled gender or call rate < 95% as well as duplicates and individuals identified as ethnic outliers. We applied a > 95% genotype call rate filer for the inclusion of SNPs. Genotype imputations were performed to the 1000 Genome reference panel (phase 1) with SHAPEIT and IMPUTE2. Written informed consent was obtained at recruitment from all participating mothers on behalf of themselves and their children.

**DNBC-PTB**

The DNBC-PTB is a nested study within the DNBC focused on the genetics of preterm birth. GWAS genotyping of the DNBC-PTB samples was done using the Illumina 660 Quad chip. Prior to imputation, we required participants to have a genotype call rate >96%, and we

excluded SNPs based on a missing rate >2%, deviation from Hardy-Weinberg equilibrium (P<10^-6^), and a minor allele frequency <1%. We also excluded samples based on heterozygosity (>3SD from the mean), non-European ancestry (>6SD from the mean in any of the first 5 principal components), potential sex discrepancy, and Mendelian inconsistency. The GWAS data were imputed with SHAPEIT and IMPUTE2 using the 1000 Genomes phase I data as reference set. SNPTEST was used for association analysis. The BMI analysis was based on 1007 children (524 boys, 483 girls) and SNPTEST was used for association analysis.

**EDEN**

The EDEN Mother-Child Cohort Study EDEN is a population-based, prospective, mother-child cohort study on prenatal and early postnatal nutritional, environmental, and social determinants of the children’s development and health. The study was approved by the Ethics Committee of Kremlin Bicêtre (France) and by the Data Protection Authority Comission Nationale de l’Informatique et des Libertés. All mothers provided written informed consent for themselves and their child. Recruitment of pregnant women expecting singletons took place between 2003 and 2006 at the University Hospitals in Poitiers and Nancy, France. At birth, infants were weighed using electronic scales (Seca Ltd), and length was measured using a wooden somatometer (Testut). At age 1 year, the infants’ weight was obtained by subtraction of the weigh tof the mother alone (Terraillon SL-351) fromwhen holding their infant wearing light clothes; infant length was measured using a somatometer (NMMedical). At age 5 years, children were weighed with electronic scales (Seca Ltd), and standing height was measured with a wall-mounted stadiometer (Seca Ltd).

DNA was extracted from cord blood samples collected at birth. Genotypes at the SLC39A8 (rs13107325) locus was measured at the Medical Research Council Epidemiology Unit, Cambridge (iPLEX platform;Sequenom). The variant passed genotyping quality control criteria (call rate>95%; Hardy-Weinberg equilibrium, P > .01)

**EFSOCH**

The Exeter Family Study Of Childhood Health (EFSOCH) recruited 1017 families from a postcode-defined area of central Exeter, UK[23]. Detailed anthropometric measurements were taken from both parents at 28 weeks of gestation, and from their children at birth, 12 weeks, 1 year and 2 years of age. Fasting blood samples were taken from the parents at 28 weeks of gestation, and an umbilical cord blood sample at delivery. These were used for biochemical analysis and DNA extraction. Measurements taken on the offspring at birth, 12 weeks of age, and 1 and 2 years of age include length (to nearest 0.1 cm using the Harpenden stadiometer) and weight (to nearest 0.1 kg using Soehnle scales)[24]. The measurements at 2 years were analysed for the current study.
 Genotyping of the whole EFSOCH sample (2768 mothers, fathers and children) was performed using the Illumina Infinium HumanCoreExome-24 array (n=551,839 SNPs/indels). The genotyping has been described previously[25]. Included samples were of European ancestry (assessed using flashPCA[26]) had genotype call rate >98%. Genotype data was used to validate phenotypic sex. Kinship was validated using King[27]. SNPs were removed if they had call rates <95% , showed evidence of deviation from Hardy-Weinberg equilibrium (P<1x10-6), or had a minor allele frequency (MAF) <1%. Imputation was performed using the Michigan imputation server and samples were imputed to the Haplotype Reference Consortium HRC v1.1 reference panel.

**FAMILY**

The Family Atherosclerosis Monitoring In earLY life (FAMILY) study was designed to longitudinally examine the fetal and early childhood determinants for the development of adiposity, cardiovascular diseases and atherosclerosis in children and has been described in detail previously[28]. Briefly, 857 families including 901 newborns, 857 mothers and 530 fathers were enrolled from three hospitals in the greater Hamilton region (Ontario, Canada) from 2004 to 2009 and were followed for up to 5 years, with a planned follow-up for 10 years or more. We selected singletons (n=816 families) into our study because twins or triplets have a strong impact on birthweight and postnatal growth velocity. Genomic DNA was extracted from buffy coats and genotyping was conducted using the Illumina Cardio-Metabochip (San Diego, CA, USA). Standard procedures were conducted to assess the quality of genotyping. We found that 26 individuals had SNP missingness rates >10%, 16 individuals were from 5 families having non-biological fathers, and 11 individuals had sex-discordance between reported and SNP-identified sex; all of these were excluded from the analysis. Six pairs of individuals had cryptic relatedness (2^nd^ degree relatives) and one of each pair was randomly selected for analysis. The self-reported ethnicity was verified by principal component analysis (PCA) across array-wide SNPs (~200K) using EIGENSTRAT and the first 10 axes were used to adjust for population stratification. The majority of the FAMILY participants were white Caucasians (92.8% mothers, 89.3% fathers and 91.1% children). Considering the inclusion criteria of the present study, 543 children were included in the final analysis. Informed consent was obtained from all the adult participants, and the parents provided consent for their children. All experiments were performed in accordance with relevant guidelines and regulations. The research ethics boards at Hamilton Health Sciences and St Joseph’s Health Center in Hamilton, and Joseph Brant Memorial Hospital in Burlington, Ontario, Canada approved the FAMILY study.

**French Young**

We studied 670 French obese children, recruited through a multi-media campaign by the CNRS UMR8199 unit in Lille, in the Department of Pediatric Endocrinology of Jeanne de Flandres Hospital or in the Toulouse Children’s Hospital[29]. Obese children, recruited by the CNRS UMR8199 unit and by the Jeanne de Flandres hospital between 1997 and 2007, are issued from pedigrees having at least one obese child with a BMI ≥ 97th percentile for sex and age and both parents. Additional obese children were recruited in Toulouse Children’s Hospital between 1997 and 2001 during pediatric obesity consultation and all harbor a BMI ≥ 97th percentile for sex and age. The 349 lean French children were selected from the STANISLAS family study[30]^30^ or from the Fleurbaix Laventie Ville Santé II study, both being family-based recruitments[31]. The Fleurbaix Laventie Ville Santé II Study includes 224 nuclear families recruited in 1999 and representative of the Northern France general population. The STANISLAS cohort includes 1006 families consisting of the two biological parents with at least two children, recruited in the Nancy area between September 1993 and August 1995 and representative of the Eastern France general population. Cases and controls were genotyped using the Illumina Human CNV370-Duo array. Imputation was performed using SHAPEIT (for the pre-phasing step) and IMPUTE 2 (for the actual imputation) based on the 1,000 Genomes reference panel (release date: March 2012). Cases and controls were imputed separately. Prior to imputation, a quality control filter on genotyped SNPs was applied: i) call rate >= 95%, ii) *P*-value of the HWE test > 0.0001.
 **Generation R Study (Generation R)**

The Generation R Study is a population-based prospective cohort study from fetal life until young adulthood. All children were born between April 2002 and January 2006. This study is designed to identify early environmental and genetic determinants of growth, development and health from fetal life until young adulthood and has been described previously in detail[32]. Detailed measurements were performed using ultrasound and physical examinations, biological samples and advanced imaging techniques. The study has been approved by the Medical Ethics Committee of the Erasmus Medical Center, Rotterdam. Written informed consent was obtained from all participants.

Analysis were restricted to individuals of European ethnic origin with genome-wide data and at least one BMI measurement available between 2 and 6 years of age. Length was measured to the nearest millimeter and weight was recorded by well-trained staff in community health centers using standardized procedures[32].

Cord blood for DNA isolation was available in 58% of all live-born participating children. Sex-mismatch rate between genome based sex and midwife-record based sex was low (<0.5%), indicating that possible contamination of maternal DNA was extremely low. Missing cord blood samples were mainly due to logistical constraints at the delivery. Genome-wide association scans (GWAs) were run using the Illumina 610 Quad and 660 platforms[32]. MACH (version 1.0.15) software was used to impute genotypes to the 1000 Genomes (March 2010 release) cosmopolitan panel[33,34]. Before imputation, SNPs were excluded if they had high levels of missing data (SNP call rate <98%), strong departures from Hardy-Weinberg equilibrium (*P*-value <1 x10^-6^), or low MAF (<1%) (20).

**German Infant Study on the influence of Nutrition Intervention PLUS environmental and genetic influences on allergy development & Influence of life-style factors on the development of the immune system and allergies in East and West Germany (GINIplus&LISA)**

The influence of Life-style factors on the development of the Immune System and Allergies in East and West Germany (LISA) Study is a population based birth cohort study. A total of 3097 healthy, mature (gestational age over 37 weeks) neonates with a birth weight over 2500g were recruited between 1997 and 1999 in Munich, Leipzig, Wesel and Bad Honnef.

A total of 5991 mothers and their newborns were recruited into the German Infant study on the influence of Nutrition Intervention PLUS environmental and genetic influences on allergy development (GINIplus) between September 1995 and June 1998 in Munich and Wesel. Detailed descriptions of the LISA and GINIplus studies have been published elsewhere[35,36].

Weight and height were measured in both studies at age 2, 4, and 5 years by the family physician and at age 10 years by the physician of the study or by the parents. Sex- and age-adjusted standard deviation scores (SDS) were calculated using LMSGrowth ([http://www.healthforallchildren.co.uk](http://www.healthforallchildren.co.uk/), reference British1990). For both studies, approval by the local Ethics Committees and written consent from participant’s families were obtained.

In the discovery analysis, 1471 children from the GINIplus&LISA study from Munich with genome-wide data were included. DNA was analyzed using the Affymetrix Human SNP Array 5.0 or 6.0 for each individual. Genome-wide data was called using BRLMM-P algorithm (Affy 5.0) or BIRDSEEDv2 (Affy 6.0) and imputed after quality control (MAF>1%, HWE *P*-value >1 x 10^-6^, call rate per SNP and person >95%) in IMPUTE. Genome-wide association analysis of BMI was carried out in SNPTEST V2.

**GOYA (Male)**

The cohort was derived from a draft board examination cohort for men, constituted by young Danish adults with negligible admixture of other ethnicities. These men also had measurements of height and weight in school and the data were extracted from the school health records. BMI in the current study was calculated from yearly measurements of height and weight at ages between 7 and 10 years. Cases are defined as having at least one BMI above the age specific 95% percentile and controls as having all BMI below the 50th percentile. The controls were used during the discovery stage and both cases and controls in the replication phase.

The study was approved by the regional scientific ethics committee and by the Danish Data Protection Board. Genome-wide genotyping on the Illumina610 quad BeadChip was carried out at the Centre National de Génotypage (CNG), Evry, France.

Pre imputation quality control involved:

1. SNP exclusions based on a. Call Rate <95%, b. Hardy Weinberg Equilibrium (HWE) *p*<1e-07,  and c. Minor allele frequency (MAF) < 1%, and

2. Individual based exclusions: a. Call Rate <95%, b. gender mismatch, c. heterozygosity (HET<0.302 or HET>0.35 were removed), d. Relatedness (IBD>0.2 removed), and e. Non CEU ancestry individuals removed through MDS based clustering.

We carried out Phasing using mach-1.0.16 and imputation using minimac-omp based on the GIANT reference panel: 1000 Genomes Phase 1 Release version 3. Link: ALL GIANT.phase1_release_v3.20101123.snps_indels_svs.genotypes.refpanel.ALL.vcf.gz.tgz

**Helsinki Birth Cohort Study (HBCS)**The Helsinki Birth Cohort Study (HBCS) is a population-based prospective cohort of singletons born at the Helsinki University Central Hospital between the years 1934 and 1944. Birth records on 4630 men and 4130 women who lived in Finland in 1971 were taken, including measurements of weight and length. Serial measurements of height and weight were extracted from child welfare clinic and school health clinic records, with an average of ten measurements between birth and 2 years, and eight measurements between 2 and 11 years of age. Between 2000 and 2002, a representative subset of 928 males and 1075 females returned for clinical examinations. At this visit, blood was taken for DNA extraction. Genotyping was performed on a custom Illumina 670 Quad platform at the Wellcome Trust Sanger Centre. Quality control was performed before imputation (excluding genotypes with call rate <0.95, MAF <0.01, HWE *P*-value <1 x 10^-6^) with MACH. The current analysis includes 1575 males and females (43% male) with BMI adjusted to their exact birthday at age 10. Informed consent was collected from all study participants. The study design was approved by the local ethics committee.

**INfancia y Medio Ambiente [Environment and Childhood] (INMA) Project**Population-based birth cohorts were established as part of the INMA – INfancia y Medio Ambiente [Environment and Childhood] Project in several regions of Spain following a common protocol. This project aims to study the associations between pre- and postnatal environmental exposures and growth, health, and development from early foetal life until adolescence and has been described previously in detail[37]. Pregnant women were enrolled during the 1st trimester of pregnancy at public primary health care centers or public hospitals. Detailed measurements were performed using ultrasound and physical examinations and biological samples were collected. Informed consent was obtained from all participants and the study was approved by the Hospital Ethics Committees in each participating region.

This particular analysis uses the INMA cohorts of Menorca (MEN), Valencia (VAL), Sabadell (SAB) in the discovery phase, and of Gipuzkoa (GIP) in the replication phase. Analyses were restricted to individuals of European ethnic origin with genome-wide data and a BMI measurement available between 2 and 10 years of age. Weight and height were measured with calibrated scales and a wall-mounted stadiometer, respectively, with the children standing in light clothing and barefoot, by trained field workers. BMI was calculated as weight in kilograms divided by the square of height in meters. Sex- and age-adjusted standard deviation scores (SDS) were constructed using LMS growth (Pan H, Cole TJ, 2012. http://www.healthforallchildren.co.uk).

DNA was obtained from cord blood, whole blood collected at 4y or saliva using the Chemagen protocol at the Spanish National Genotyping Centre (CEGEN). Children whose parents reported to be white and to be born in Spain or in European countries and that were not lost during the follow-up were selected for genotyping. Genome-wide genotyping was performed using the HumanOmni1-Quad Beadchip (Illumina) at CEGEN (MEN, SAB, VAL subcohorts) and GSA Beadchip (Illumina) at the Human Genotyping Facility (HuGeF), Dept Internal Medicine, Erasmus MC, The Netherlands (GIP subcohort). Genotype calling was done using the GeneTrain2.0 algorithm based on HapMap clusters implemented in the GenomeStudio software. Quality control was done using PLINK and following standard criteria. First of all, SNPs were flipped to the human genome + strand. We applied the following initial quality control thresholds: sample call rate>98% and/or LRR SD<0.3. Then, we checked sex, relatedness, heterozygosity and population stratification. Genetic variants were filtered for SNP call rate>95%, MAF>1% and HWE *P*-value> 1.10E-06. Imputation of genetic variants was done using IMPUTE V2 and the cosmopolitan 1000 genome panel (release March 2012) (MEN, SAB, VAL) and the Michigan imputation server using the Haplotype Reference Consortium HRC v1.1 reference panel (GIP).

**LIFE-Child**

LIFE Child (NCT02550236) is an ongoing regional population-based study with comprehensive phenotyping including anthropometric and clinical data, laboratory data, genetic data and psychoscocial assessment in children and parents conducted in the city of Leipzig, Germany[38,39]. As a part of LIFE, the Leipzig Research Center for Civilization Diseases, it aims to monitor healthy child development from birth to adulthood and to understand the development of civilization diseases such as obesity. LEIPZIG childhood obesity cohort/LIFE Child Obesity is an obesity enriched subset with additional metabolic and cardiovascular phenotyping including OGTT. All procedures are performed in accordance with the ethical standards of the institutional and/or national research committee and with the 1964 Helsinki declaration and its later amendments or comparable ethical standards. All legal guardians gave written informed consent, and the study has been approved by Ethics Committee of the University of Leipzig.

**MAAS**

The Manchester Asthma and Allergy Study is an unselected (i.a. population-based), birth cohort study[40-44]. The setting is the maternity catchment area of Wythenshawe and Stepping Hill Hospitals, comprising of 50 square miles of South Manchester and Cheshire, UK, a stable mixed urban-rural population. Study was approved by the Local Research Ethics Committee. Informed consent was obtained from all parents.

*Screening & Recruitment*

All pregnant women were screened for eligibility at antenatal visits (8th-10th week of pregnancy). The study was explained to the parents, and informed consent for initial questionnaires and skin prick testing was obtained. Both parents completed a questionnaire about their and their partner’s history of asthma and allergic diseases and smoking habits. If the pregnant woman’s partner was not present at the antenatal clinic visit, an invitation was sent for him to attend an open-access evening clinic for skin prick testing and questionnaire. Once both parents had completed questionnaires and skin prick testing, a full explanation of the proposed future follow-up for the child was given. Of the 1499 couples who met the inclusion criteria (<10 weeks of pregnancy, maternal age >18 years, questionnaire and skin test data available for both parents), 288 declined to take part in the study. A total of 1185 participants had at least some evaluable data.

*Follow-up*

The children have been followed prospectively, and attended review clinics at ages 1, 3, 5, 8 and 11 years.

*Phenotyping*

BMI was measured as outlined in Wang *et al*[45]. Briefly we measured weight, without shoes or outer clothing (to nearest 0.01kg), using a weighing scale, and height (to nearest 0.1cm) using a stadiometer.

*Genotyping*

DNA samples were genotyping on an illumina 610 quad chip. The illumina genotypes were called using the Illumina GenCall application following the manufacturer’s instructions. Quality control criteria for samples included: 97% call rate, exclusion of samples with an outlier autosomal heterozygosity (scree-plot visualisation) gender validation and sequenome genotype concordance. Quality control criteria for SNPs included a 95% call rate, HWE *P*-value > 5.9 x 10^-7^, MAF > 0.005. Genotypes were prephased with SHAPIT shapeit.v2.644 and imputed with IMPUTE version 2.2.2 with 1March2012 release of the 1000 genomes Phase I integrated variant set release (v3; updated 26 Aug 2012) reference genotypes. Association analysis was carried out using SNPTEST version 2.4.1 using frequentist with the score method.

**Northern Finnish Birth Cohort Study 1966 (NFBC1966)**

The Northern Finland Birth Cohort study 1966 (NFBC1966) (http://www.oulu.fi/nfbc/) includes 12,058 live born individuals, of European descent, with expected dates of birth during 1966 in the two northernmost provinces of Finland, Oulu and Lapland [46]. The University of Oulu Ethics Committee and the Ethical Committee of Northern Ostrobothnia Hospital District have approved the study. The data on all cohort members were prospectively collected since pregnancy and supplemented at the ages of 1, 14, 31 and 46 years. Growth measurements were obtained from communal child health clinics or from clinical examinations [47]. All those living in northern Finland or in the capital area were invited to a clinical examination and blood sampling at age 31 years [48]. BMI used in this analysis was calculated from the measurements of height and weight obtained throughout the childhood from child clinics. For this analysis a total of 52,486 BMI measures obtained at ages 2-18 years were used. Blood samples were drawn and DNA was extracted when individuals were 31 years old. Participants provided written informed consent.

DNA was successfully extracted for 5,753 participants from fasted blood samples. Illumina’s HumanCNV370-Duo Analysis BeadChip was used for genome-wide genotyping. After SNP filtering (SNPs with MAF < 0.05, HWE p<5.7x10-7 or call rate < 0.95 removed) and sample filtering (sample call rate < 0.95, mean heterozygosity < 0.29, MDS outliers, duplicates, contaminated samples, IBS pairwise sharing < 0.20, consent withdrawal or gender mismatch excluded), imputation was done for 1000G Phase 1 reference panel using 364,590 SNPs for 5402 participants.

**Northern Finland Birth Cohort Study 1986 (NFBC1986)**

The NFBC1986 includes 9,432 live born children with expected dates of birth between 1st July 1985 and 30th June 1986 in the two northernmost provinces of Finland, Oulu and Lapland. The University of Oulu Ethics Committee and the Ethical Committee of Northern Ostrobothnia Hospital District have approved the study. The cohort has been followed up since early pregnancy until young adulthood. Growth measurements were obtained from communal child health clinics or from clinical measurements. All those alive with known address were invited to a clinical examination at the age of 15 to 16 years, when blood samples were drawn. Participants provided written informed consent. BMI was calculated from the measurements height and weight obtained at ages 2-18 years. A total of 65,034 BMI measures were used in this analysis. DNA was extracted for 6,266 subjects using standard methods. A total of 3,834 samples were genotyped using Illumina HumanOmniExpressExome-8v1.2 platform and Beadstudio calling algorithm. After SNP filtering (SNPs with HWE p<1x10-4, call rate < 0.99 removed) and samples filtering (call rate < 0.95, mean heterozygosity < 0.305, IBS pairwise sharing < 0.2, gender mismatch, duplicate samples or consent withdrawal), genotype data was available for 3,743 adolescents. This includes a selected set of 372 individuals exposed to gestational diabetes, gestational hypertensive disorders and preterm birth, and the rest is a random sample. Imputation was done based on 889,119 SNPs for 1000G Phase 3 imputation panel. For the current BMI analyses, full data with growth and GWAS information were available for 1,506 individuals.

**Norwegian Mother, Father and Child (MoBa) Cohort Study (Two contributing projects; Pål R. Njølstad (6828 samples in replication) & Bo Jacobsson (522 samples in discovery))**

The Norwegian Mother, Father and Child (MoBa) Cohort Study ([www.fhi.no/moba](http://www.fhi.no/moba)) is a prospective population-based pregnancy cohort study conducted by the Norwegian Institute of Public Health[49-52]. Participants were recruited from all over Norway from 1999-2008. The women consented to participation in 40.6% of the pregnancies. The cohort now includes 114.500 children, 95.200 mothers and 75.200 fathers.

Blood samples were obtained from both parents during pregnancy and from mothers and children (umbilical cord) at birth. MoBa has obtained a licence from the Norwegian Data Inspectorate. Measurements of height and weight were performed at health stations by health personnel during routine health station follow-ups.
 The establishment of MoBa and initial data collection was based on a license from the Norwegian Data protection agency and approval from The Regional Committee for Medical Research Ethics. The MoBa cohort is currently regulated by the Norwegian Health Registry Act. The current study was approved by The Regional Committee for Medical Research Ethics.

Pål R. Njølstad: Study was approved by the Regional Committee for Medical and Health Research Ethics in Norway (2012/67) on version 9 of the quality assured data files. 11.000 samples were genotyped using the Illumina HumanCoreExome-12 v.1.1 and HumanCoreExome-24 v.1.0 chips. Genotypes were called using Illumina Genome Studio using only samples with call rate >=0.98 and GenCall score >= 0.15 for defining cluster positions. We excluded variants with low call rates, signal intensity, quality scores, heterozygote excess and deviation from Hardy-Weinberg equilibrium based on the following QC parameters: call rate <98%, cluster separation <0.4, 10% GC-score <0.3, AA T Dev > 0.025, HWE P-value <1 x 10-6. As 214 samples had a duplicate available, we also removed SNPs with more than three discordant genotypes (where both samples had a non-missing genotype). Samples were excluded based on call rate <98% and heterozygosity excess >4SD. Study participants with non-Norwegian ancestry were excluded after merging with samples from the HapMap project (ver. 3) to remove ethnic outliers. Sample pairs with PI_HAT > 0.1 in identical-by-descent (IBD) calculations were resolved by removing the sample with the highest missing-phenotype value, or by random in case of ties. Of the 9256 samples with Norwegian ancestry 6828 were eligible for analyses in the current study.

Bo Jacobsson: Study is based on version 6 of the quality-assured data files released for research in 2011, and was approved by The Regional Committee for Medical Research Ethics. This sub-study included only 522 samples. BMI was calculated based on self-reported height and weight in questionnaire.

**The Netherlands Twin Register** **(NTR)**

*Phenotyping*

The Netherlands Twin Register (NTR) collects data on development and growth in twins who are registered at birth by their parents[53,54]. In the NTR, data on BMI were available from the surveys sent out at ages 2, 3, 5, 7 and 10 years. At age 2 and 3, parents were asked to fill out all the dates and measures of height and weight as measured by the Dutch Community Health Services at regular intervals. At age 5, parents were asked to fill out the height and weight of the twins with the dates of measurement since the 3th birthday. At age 7 and 10, parents were asked to fill out the current height and weight of the twins with the date of assessment. In addition, laboratory measures were available for a group of individuals that participated in smaller subprojects^55^. Improbable data points were checked for data entry errors and corrected or excluded where necessary. Sex- and age adjusted standardized BMI scores were calculated with the 1990 British growth reference charts using the program LMSGrowth (available from http://www.healthforallchildren.co.uk). The measurement point between 24 and 120 months closest to 120 months was selected.

*Genotyping and Imputation*

Genotyping was done on the Affymetrix 6.0 platform[56]. SNPs were lifted over to build 37 (HG19) and strand aligned to 1000 Genomes phase I Interim release ALL panel (23 Nov 2010 sequence and June 2011 haplotypes). SNPs were excluded if alleles did not match or differed in frequency (>0.20) with the imputation reference set, if MAF was <1%, HWE p-value was < 0.00001 or call rate was <95%. Sample were excluded if sex did not match between genotyped and expected, if Plink F-value (heterozygosity) was > 0.10 or < -0.10 or if genotype call rate was < 90%. One individual from each monozygotic (MZ) twin pair was excluded. For imputation this dataset was merged with other NTR data, genotyped on different genotyping platforms, namely Affymetrix Perlegen 5.0, Illumina 370, Illumina 660 and Illumina Omni Express 1M.  The same QC steps were applied to each dataset. Samples additionally were checked for IBD within the merged dataset and were excluded if IBD did not match expected family relationships. Samples genotyped on different platforms have concordance rate > 99.0% or were removed otherwise. Same HWE, MAF filters as well as allele frequency comparison with reference set were applied to the merged dataset. Finally SNPs with MAF within range if 0.35 to 0.50 and allele combinations C/G and A/T were removed to avoid strand issues during imputation. The data were phased using Mach and imputed using Minimac. After imputation SNPs were removed, if they showed association with genotyping platform (p < 0.00001), if HWE p-value for the full dataset was < 0.000001, if Mendelian error rate was > 2%, if MAF was < 0.004, or if imputation quality R2 was < 0.30.

*Statistical Analysis*

Children with non-Dutch / non-European ancestry were excluded from the analyses. The final sample size for the analysis of BMI was 1,767 individuals. Analyses were performed in Plink in a linear regression framework; age and sex adjusted standard deviation scores were regressed on genome-wide SNP data and 3 genotyping chips. Data included one twin of each monozygotic twin pair, and both dizygotic twins. Standard errors were adjusted for family clustering using the --family option in Plink.  

**The Physical Activity and Nutrition in Children (PANIC) Study**
The Physical Activity and Nutrition in Children (PANIC) Study is an 8-year controlled physical activity and dietary intervention study in a population sample of children from the city of Kuopio, Finland (<http://www.panicstudy.fi/en/panic-study-briefly>)[57]. The main aims of the study are to investigate behavioral, biological, environmental, and genetic risk factors for overweight, type 2 diabetes, atherosclerotic cardiovascular diseases, musculoskeletal diseases, psychic problems, dementia, and oral health problems and the effects of a long-term physical activity and dietary intervention on risk factors for these chronic diseases and conditions. Altogether 512 children 6-9 years of age participated in the baseline examinations in 2007-2009. Six children were excluded from the study at baseline because of physical disabilities that could hamper participation in the intervention or no time or motivation to attend in the study. The remaining 506 children were divided in the physical activity and dietary intervention group and the control group. The intervention included six physical activity and dietary counseling sessions for the children and their parents or caregivers during the 2-year follow-up (0.5, 1.5, 3, 6, 12, and 18 months after baseline) (<http://www.panicstudy.fi/en/intervention>). The control group received general advice on health improving physical activity and diet according to the Finnish recommendations at baseline but no active intervention. Altogether 440 (87%) of the 506 children participated in the 2-year follow-up examinations in 2009-2011. The intervention was continued between 2-year and 8-year follow-up examinations and included seven physical activity and dietary counseling sessions (24, 36, 48, 60, 72, 84, and 96 months after baseline). Altogether 277 adolescents participated in the 8-year follow-up examinations in 2016-2017. A large number of behavioral, biological, environmental, and genetic risk factors for obesity, type 2 diabetes, atherosclerotic cardiovascular diseases, musculoskeletal diseases, psychic problems, dementia, and oral health problems were assessed between fetal period and adolescence (<http://www.panicstudy.fi/en/assessments1>). Most of the assessments were performed at baseline in 2007-2009, at 2-year follow-up in 2009-2011, and at 8-year follow-up in 2016-2017. The most important assessments will also be repeated at 14-year follow-up in 2021-2023. The PANIC study protocol was approved by the Research Ethics Committee of the Hospital District of Northern Savo. A written informed consent was acquired from the parents or caregivers of the children, and the children also provided their assent to participation.

The present analyses were carried out among children of European ethnic origin with genome-wide data and BMI measured at the latest time point between 6 and 10 years of age. The analyses included 374 children (193 boys, 181 girls)  in the discovery phase. Body weight and height were measured without shoes and in light clothing in the PANIC study laboratory (72%) or were obtained from the health registers of the city of Kuopio (28%). Body weight was measured the children having fasted for 12 hours, having emptied the bladder, and standing in light underwear using a calibrated InBody^®^720 bioelectrical impedance device (Biospace Co. Ltd., Seoul, South Korea) to accuracy of 0.1 kg. Body height was measured using a wall-mounted stadiometer to accuracy of 0.1 cm. Sex- and age-adjusted weight, height, and BMI standard deviation scores (SDS) were computed using the LMSgrowth software ([http://www.healthforallchildren.co.uk](http://www.healthforallchildren.co.uk/)) using the British 1990 growth references. Genomic DNA was isolated from the blood mononuclear cells using the QIAamp DNA Blood kit (Qiagen, Hilden, Germany). Genotyping was performed using the Illumina Custom Infinium CardioMetabo BeadChip (Illumina, San Diego, CA, USA) in the discovery phase and the Illumina Infinium HumanCoreExome BeadChip in the replication phase (Illumina, San Diego, CA, USA).

**Prevention and incidence of asthma and mite allergy birth cohort study - (PIAMA)**
PIAMA is a birth cohort study consisting of two parts: a placebo controlled intervention study in which the effect of mite impermeable mattress covers on the development of asthma and allergy was studied and a natural history study in which no intervention took place. Details of the study design have been published previously[58]. Recruitment took place in 1996-1997 through prenatal clinics. A screening questionnaire was distributed to pregnant women visiting one of 52 prenatal clinics at three regions in the Netherlands. A total of 10,232 pregnant women completed a validated screening questionnaire. Mothers reporting a history of asthma, current hay fever or allergy to pets or house dust mite were defined as allergic. Based on this screening, 7,862 women were invited to participate, of whom 4,146 women (1,327 allergic and 2,819 non-allergic) gave written informed consent. Follow-up of the children took place at 3 months of age and yearly from 1 to 8 years of age. The Medical Ethical Committees of the participating institutes approved the study, and all participants gave written informed consent. DNA was collected from 2,162 children, and height and weight measures were obtained in 2,440 children during medical examinations at age 4 and/or 8 years. Genome-wide genotyping was performed in two phases. The first phase was performed within the framework of the GABRIEL Consortium using an Illumina Human 610K quad array[59]. Genotypes were available from 172 children with asthma and from 187 controls after quality control. A second group of 268 children who were more extensively examined during follow up was genotyped with an Illumina HumanOmniExpress array. A final group of 1377 children was genotyped with the Illumina Human Omni Express Exome Array.  The current replication analysis was restricted to individuals of European ethnic origin with genome-wide data and phenotype information (n=1958 children).

**SCOOP**
The Severe Childhood Onset Obesity Project (SCOOP) cohort comprised of ~4,800 British individuals of European ancestry is a sub-cohort of the Genetics of Obesity Study (GOOS)[60] (https://www.goos.org.uk/), which has recruited 7,000 individuals with severe early-onset obesity (BMI standard deviation score (SDS) > 3; onset of obesity before the age of 10 years). SCOOP individuals with congenital leptin deficiency or mutations in the melanocortin 4 receptor gene (MC4R) were excluded by prior Sanger sequencing. The current replication analysis includes 685 children aged 2-10 years genotyped on the Illumina HumanCoreExome-12v1-1 Beadchip imputed to the UK10K and 1000G Phase 3 reference panel[61] as described previously[62].

**SKOT I**
The Småbørns Kost Og Trivsel I (SKOT I) study is a population-based prospective cohort and recruitment and inclusion criteria have been described in detail elsewhere[63]. In short, the 329 children included in SKOT I were healthy singletons randomly recruited from the National Civil Registry and living in Copenhagen and Frederiksberg municipality (Denmark). The included children were born at term and had Danish-speaking parents. Prior to participation, written informed consent was obtained from all parents of the children. The Committees on Biomedical Research Ethics for the Capital Region of Denmark approved the study protocol (H-KF-2007-0003).The children were genotyped using the Illumina Infinium HumanCoreExome Beadchip (Illumina, San Diego, CA, USA) and genotypes were called using the Genotyping module, version 1.9.4 of GenomeStudio software, Version 2011.1 (Illumina). During genotype quality control, we excluded closely related individuals and samples with extreme inbreeding coefficients, mislabeled gender or call rate < 95% as well as duplicates and individuals identified as ethnic outliers. We applied a > 95% genotype call rate filer for the inclusion of SNPs. Additional genotypes were imputed into 1000 Genomes (phase 1) using IMPUTE2. Prior to participation, written informed consent was obtained from all parents of the children included in SKOT I. The Committees on Biomedical Research Ethics of the Capital Region of Denmark approved the study protocol of SKOT-I (H-KF-2007-0003).

**Special Turku Coronary Risk Factor Intervention Project (STRIP)**

The STRIP study is a prospective randomised life-style intervention trial that began in infancy and continued through childhood and adolescence to early adulthood[64]. Altogether 1,062 children born in 1989-1991 were recruited at the age of 5 months by the well-baby clinics in Turku, and were randomised into an intervention group (n=540) or a control group (n=522). The life-style intervention continued until the participants reached the age of 20 years (n=~500). At the moment, the study participants are 27 to 29 years old (2018).

The purpose of the life-style intervention was to reduce exposure to cardiovascular disease risk factors with main focus on diet. Primary target of the dietary counselling was replacement of saturated fat with unsaturated fat in the child’s diet. The counselling also promoted intake of vegetables, fruits, and whole-grain products, and low intake of salt. In addition to investigating the efficiency of dietary counselling in improving risk factor levels, safety of the low-saturated fat diet was assessed in terms of growth and development. The main outcome measures comprise nutrient intake, serum lipid and lipoprotein concentrations, blood pressure, measures of somatic growth and development, and ultrasonic measures of arterial intima-media thickness, elasticity and endothelial function.

Weight was measured  in light clothing using an S10 electronic scale (Soehnle, Murrhardt, Germany) to the nearest 0.1 kg and height to the nearest 0.1 cm using a Harpender stadiometer (Holtain, Crymych, U.K.). BMI was calculated in kilograms/square of height in meters[65].

Altogether, 666 STRIP children were genotyped using the custom Illumina genotyping array, Metabochip, at the Center for Inherited Disease Research, the Johns Hopkins University, USA. Genotype imputation was performed using IMPUTE2[10,11] and a reference panel from the 1000 Genomes.

The study was approved by the Joint Commission on Ethics of the Turku University and the Turku University Central Hospital. Informed consent was obtained from all parents at the beginning of the trial and from the children at 15 and 18 years of age.

**TEENS of Attica: Genes and Environment Study (TEENAGE)**
The TEENAGE study is a cross-sectional study. The study target population comprised 857 adolescent students aged 13–15 years attending the first three classes of public secondary schools located in the wider Athens area of Attica. Prior to recruitment all study participants gave their verbal assent along with their parents’/guardians’ written consent forms. The study protocol was approved by the Institutional Review Board of Harokopio University and the Greek Ministry of Education, Lifelong Learning and Religious Affairs[66]. DNA samples of 707 study participants were genotyped using Illumina HumanOmniExpress BeadChips (Illumina, San Diego, CA, USA) at the Wellcome Trust Sanger Institute, Hinxton, UK. Genotyping and data quality control have been described previously[67]. Genotypes were called using Illuminus algorithm[68] and SNPs were imputed using the program IMPUTE[69]. Body weight and height at earlier time points from recruitment were collected retrospectively from participants’ medical health records and BMI was calculated as weight (kg) / height (m^2^). Analysis was restricted to individuals with available BMI measurement at ages between 2 and 6 years of age (N=252). Sex- and age-adjusted standard deviation scores (SDS) were constructed using LMS growth (http://www.healthforallchildren.co.uk)

**TEDS**
TEDS recruited over 15,000 families of twins born in England and Wales in 1994, 1995 and 1996 and the sample remains representative of the UK population. Ethical approval for TEDS has been provided by the Institute of Psychiatry ethics committee, reference number 05/Q0706/228.

Height and weight data were collected by postal questionnaire from the parents when their children were 3, 4, 7 and 10 years of age. Correlations with measured heights and weights in a sub-sample of these children at age 11 were 0.83 and 0.90. BMI was calculated from the height and weight data (BMI = weight (kg)/(height (m)^2)) and converted to BMI z-scores. BMI z-scores take into consideration the child's age and sex. They were based on 1990 UK growth reference curves and were calculated using the program LMSGrowth (available from http://homepage.mac.com/tjcole).

DNA for 8,743 individuals (including 3,722 dizygotic co-twin samples) was extracted from saliva and buccal cheek swab samples and hybridized to HumanOmniExpressExome-8v1.2 genotyping arrays at the Institute of Psychiatry, Psychology and Neuroscience Genomics & Biomarker Core Facility, London, UK. The raw image data from the array were normalized, pre-processed, and filtered in GenomeStudio according to Illumina Exome Chip SOP v1.4. (<http://confluence.brc.iop.kcl.ac.uk:8090/display/PUB/Production+Version%3A+Illumina+Exome+Chip+SOP+v1.4>). In addition, prior to genotype calling, 919 multi-mapping SNPs and 501 samples with callrate <0.95 were removed. The ZCALL program was used to augment the genotype calling for samples and SNPs that passed the initial QC.

DNA from 3,747 samples was extracted from buccal cheek swabs and genotyped at Affymetrix, Santa Clara, California, USA. From this sample, 3,665 samples were successfully hybridized to AffymetrixGeneChip 6.0 SNP genotyping arrays (<http://www.affymetrix.com/support/technical/datasheets/genomewide_snp6_datasheet.pdf>) using experimental protocols recommended by the manufacturer (Affymetrix Inc., Santa Clara, CA). The raw image data from the arrays were normalized and pre-processed at the Wellcome Trust Sanger Institute, Hinxton, UK for genotyping as part of the Wellcome Trust Case Control Consortium 2 (<https://www.wtccc.org.uk/ccc2/>) according to the manufacturer’s guidelines (http://www.affymetrix.com/support/downloads/manuals/genomewidesnp6_manual.pdf). Genotypes for the Affymetrix arrays were called using CHIAMO (https://mathgen.stats.ox.ac.uk/genetics_software/chiamo/chiamo.html).

After initial quality control and genotype calling, the same quality control was performed on the samples genotyped on the Illumina and Affymetrix platforms separately using PLINK[70-73]

Samples were removed from subsequent analyses on the basis of call rate (<0.98), suspected non-European ancestry, heterozygosity, and relatedness other than dizygotic twin status. SNPs were excluded if the minor allele frequency was smaller than 0.5%, if more than 2% of genotype data were missing, or if the Hardy Weinberg p-value was lower than 10-5. Non-autosomal markers and indels were removed. Association between SNP and the platform, batch, plate or well on which samples were genotyped was calculated; SNPs with an effect p-value < 10-4 were excluded. A total sample of 10,346 samples (including 3,320 dizygotic twin pairs and 7,026 unrelated individuals), with 7,289 individuals and 559,772 SNPs genotyped on Illumina and 3,057 individuals and 635,269 SNPs genotyped on Affymetrix.

Genotypes from the two platforms were separately phased using EAGLE2[74] and imputed into the 1000 Genomes reference panel (phase 3) using the Positional Burrows-Wheeler Transform method[75] through the Sanger Imputation Service[76] .

**TDCOB (cases and controls)**

The Danish Childhood Obesity Data and Biobank (ClinicalTrials.giv ID-no.: NCT00928473) includes children and adolescents with normal weight, overweight or obesity. Data material was collected between Januari 2009 and March 2015. A total of 1,069 children and adolescents, aged 6-18 years, with overweight or obesity (defined as BMI-SDS > 1.28 according to Danish national standards[77] were recruited through the Children’s Obesity Clinic, Department of Pediatrics, Copenhagen University Hospital Holbaek, Denmark. Between September 2010 and March 2013, a population-based control sample of Danish children and adolescents aged 6-18 years was recruited from schools across 11 municipalities in Denmark[78]. In the discovery stage of the present analysis, children that were between 2-10 years of age at the baseline examination were included. In the replication stage of the present analysis, we included children for which we were able to retrieve information on BMI in the age range 2-10 years based on self-report, school records and other registry data. There was no overlap between children included in the discovery stage and children included in the replication stage. The TDCOB cases and controls were genotyped using the Illumina Infinium HumanCoreExome Beadchip (Illumina, San Diego, CA, USA) and genotypes were called using the Genotyping module, version 1.9.4 of GenomeStudio software, Version 2011.1 (Illumina). Individuals identified as duplicates, ethnic outliers, or with extreme inbreeding coefficients, mislabeled gender or a call-rate <95% were excluded during genotyping quality control. Additional genotypes were imputed into the 1000 genomes phase 1 panel using IMPUTE2. Informed written and oral consent was obtained from all parents (participant < 18 years) or participants. This study was approved by the Ethics Committee of Region Zealand, Denmark, (ID No. SJ-104) and by the Danish Protection Agency.

**Tracking Adolescents' Individual Lives Survey (TRAILS)**

TRAILS (TRacking Adolescents' Individual Lives Survey) is a prospective cohort study of Dutch adolescents with bi- or triennial measurements from age 11 to, at this moment, age 26 and consists of a general population and a clinical cohort[79]. Data on infant and childhood growth, including birth weight and length, were extracted from records of wellchild clinics. These clinics are attended by 95% of the Dutch population. Children attend at ages 1, 2, 3, 4, 6, 9,11, 14, and 18 months and 2, 3, 4, 5, 10, and 13 years. During all visits, weight and length/height were measured by trained nurses[80].

DNA of 1335 samples was genotyped using the llumina Cyto SNP12 v2 chip (Illumina, San Diego, CA, USA) at the department of Genetics, University Medical Center Groningen. Genotype calling and quality control were carried out using Illumina GenomeStudio and PLINK[71], respectively. Impute v2[69] was used for imputation. Association analysis was performed using SNPtest v2.5[81].

**The Raine Study**

The Raine Study was started as a randomized controlled trial to evaluate the effects of repeated ultrasound in pregnant women in Perth, Western Australia[82]. In total, 2,900 pregnant women were recruited between 1989 and 1991 prior to 18 weeks gestation at the King Edward Memorial Hospital (Perth, Western Australia). Women were randomized to repeated ultrasound measurements at 18, 24, 28, 34 and 38 weeks gestation or to a single ultrasound assessment at 18‐weeks. The cohort individuals have been assessed at average ages of 1, 2, 3, 5, 8, 10, 14, 17, 20, and 22 and both height and weight were collected at each assessment.

DNA was collected at the year 14 and 17 follow-ups. The study was conducted with appropriate institutional ethics approval, and written informed consent was obtained from mothers at all follow‐ups and participants at the year 17 follow-up. DNA was collected using standardized procedures from 74% of all adolescents who attended the 14 year follow-up on and a further 5% at the 17 year follow-up measurements. We performed high throughput genome-wide SNP genotyping using the genome‐wide Illumina 660 Quad Array for each individual. Genotype data were imputed against 1000 Genomes (March 2012 release) using MACH and Minimac after quality control (MAF>1%, HWE *P*-value >5 x 10^-7^, call rate per SNP and person >95%). Genome-wide association analysis of the obesity phenotype was carried out in Probabel.

**ACKNOWLEDGEMENTS**

**ABCD study**We thank all participating hospitals, obstetric clinics, general practitioners and primary schools for their assistance in implementing the ABCD study. We also gratefully acknowledge all the women and children who participated in this study for their cooperation.
 **Avon Longitudinal Study of Parents and Children (ALSPAC)**We are extremely grateful to all the families who took part in this study, the midwives for their help in recruiting them, and the whole ALSPAC team, which includes interviewers, computer and laboratory technicians, clerical workers, research scientists, volunteers, managers, receptionists and nurses.

**BMDCS**We are grateful for the support of Dr. Karen Winer, Scientific Director of the Bone Mineral Density in Childhood Study.

**BRain dEvelopment and Air polluTion ultrafine particles in scHool childrEn Project (BREATHE)**We are acknowledged with all the families participating into the study for their altruism and particularly to the schools Antoni Brusi, Baloo, Betània – Patmos, Centre d’estudis Montseny, Collegi Shalom, Costa i Llobera, El sagrer, Els Llorers, Escola Pia de Sarrià, Escola Pia Balmes, Escola concertada Ramon Llull, Escola Nostra Sra. de Lourdes, Escola Tècnica Professional del Clot, Ferran i Clua, Francesc Macià, Frederic Mistral, Infant Jesús, Joan Maragall, Jovellanos, La Llacuna del Poblenou, Lloret, Menéndez Pidal, Nuestra Señora del Rosario, Miralletes, Ramon Llull, Rius i Taulet, Pau Vila, Pere Vila, Pi d'en Xandri, Projecte, Prosperitat, Sant Ramon Nonat - Sagrat Cor, Santa Anna, Sant Gregori, Sagrat Cor Diputació, Tres Pins, Tomàs Moro, Torrent d'en Melis, Virolai.

**British 1958 Birth Cohort (1958BC-WTCCC and 1958BC-T1DGC)**The authors are deeply grateful to the 1958 birth cohort participants for their longstanding commitment and support, and to all staff for cohort coordination and data collection.

**Cardiovascular Risk in Young Finns Study (YFS)**We thank all the participants of the YFS study.
 **Children’s Hospital of Philadelphia (CHOP)**The authors thank the network of primary care clinicians and the patients and families for their contribution to this project and to clinical research facilitated by the Pediatric Research Consortium (PeRC) at The Children’s Hospital of Philadelphia. R. Chiavacci, E. Dabaghyan, A. (Hope) Thomas, K. Harden, A. Hill, C. Johnson-Honesty, C. Drummond, S. Harrison, F. Salley, C. Gibbons, K. Lilliston, C. Kim, E. Frackelton, F. Mentch, G. Otieno, K. Thomas, C. Hou, K. Thomas and M.L. Garris provided expert assistance with genotyping and/or data collection and management. The authors would also like to thank S. Kristinsson, L.A. Hermannsson and A. Krisbjörnsson of Raförninn ehf for extensive software design and contributions. 

**CHOP (Europe)**The authors thank the participating families and all project partners for their enthusiastic support of the project work. We thank Dr Eva Reischl and team at the Genome Analysis Center of Helmholtz Zentrum Muenchen for gentopyping and intial QC of the data and Dr Linda Broer and team at the Department of Internal Medicine, Genetic Laboratory, Erasmus Medical Center, Rotterdam, The Netherlands for extensive QC and imputation of the genotyped data. We also like to acknowledge the The European Childhood Obesity Trial Study Group for their continuous and salient support of the CHOP project: Philippe Goyens, Clotilde Carlier, Joana Hoyos, Pascale Poncelet, and Elena Dain (Universite Libre de Bruxelles – (ULB) –Brussels , Belgium); Jean-Noel Van Hees (CHC St Vincent– Françoise Martin, Annick Xhonneux, Jean-Paul Langhendries, and Jean-Noel Van Hees - Liège-Rocourt, Belgium); Ricardo Closa-Monasterolo, Joaquin Escribano, Veronica Luque, Georgina Mendez, Natalia Ferre, and Marta Zaragoza-Jordana (Universitat Rovira i Virgili, Institut d’Investigacio´ Sanitaria Pere Virgili, Taragona, Spain); Marcello Giovannini, Enrica Riva, Carlo Agostoni, Silvia Scaglioni, Elvira Verduci, Fiammetta Vecchi, and Alice Re Dionigi (University of Milano, Milano, Italy); Jerzy Socha, Piotr Socha and Anna Stolarczyk (Children’s Memorial Health Institute, Department of Gastroenterology, Hepatology and Immunology, Warsaw, Poland); Anna Dobrzanska and Dariusz Gruszfeld (Children’s Memorial Health Institute, Neonatal Intensive Care Unit, Warsaw, Poland); Roman Janas (Children’s Memorial Health Institute, Diagnostic Laboratory, Warsaw, Poland); Emmanuel Perrin (Danone Research Centre for Specialized Nutrition, Schiphol, the Netherlands); Rudiger von Kries (Division of Pediatric Epidemiology, Institute of Social Pediatrics and Adolescent Medicine, Ludwig Maximilians University of Munich, Munich, Germany); Helfried Groebe, Anna Reith, and Renate Hofmann (Klinikum Nurnberg Sued, Nurnberg, Germany); and Berthold Koletzko, Veit Grote, Martina Weber, Peter Rzehak, Sonia Schiess, Jeannette Beyer, Michaela Fritsch, Uschi Handel, Ingrid Pawellek, Sabine Verwied-Jorky, Iris Hannibal, Hans Demmelmair, Gudrun Haile, and Melissa Theurich (Division of Nutritional Medicine and Metabolism, Dr von Hauner Childrens Hospital, Ludwig-Maximilians Universität München (LMU), Munich, Germany).

**Copenhagen Study on Asthma in Childhood 2000 birth cohort (COPSAC2000)**We gratefully express our gratitude to the children and families of the COPSAC2000 cohort study for all their support and commitment. We acknowledge and appreciate the unique efforts of the COPSAC research team.

**Copenhagen Study on Asthma in Childhood 2010 birth cohort (COPSAC2010)**We gratefully express our gratitude to the children and families of the COPSAC2010 cohort study for all their support and commitment. We acknowledge and appreciate the unique efforts of the COPSAC research team.

**DNBC-PTB and DNBC-Goya offspring**We are very grateful to all DNBC families who took part in the study. We would also like to thank everyone involved in data collection and biological material handling.

**EDEN**We are extremely grateful to all the families who took part in this study, the midwives and psychologists for recruiting and following them, and the whole EDEN team, including research scientists, engineers, technicians and managers. We acknowledge the commitment of the EDEN mother-child cohort study group: I Annesi-Maesano, JY Bernard, J Botton, M-A Charles, P Dargent-Molina, B de Lauzon-Guillain, P Ducimetière, M de Agostini, B Foliguet, A Forhan, X Fritel, A Germa, V Goua, R Hankard, B Heude, M Kaminski, B Larroque, N Lelong, J Lepeule, G Magnin, L Marchand, C Nabet, F Pierre, R Slama, MJ Saurel-Cubizolles, M Schweitzer, O Thiebaugeorges.

**EFSOCH**We are grateful to all EFSOCH study participants and study staff.

**FAMILY**We would like to thank all the participants and investigators of the FAMILY study. We acknowledge the internal support from the Population Health Research Institute for centralizing the data collection

**French Young**We are grateful to the subjects and families who participated to this study.

**Generation R Study (Generation R)**The Generation R Study is conducted by the Erasmus Medical Center in close collaboration with the School of Law and Faculty of Social Sciences of the Erasmus University Rotterdam, the Municipal Health Service Rotterdam area, Rotterdam, the Rotterdam Homecare Foundation, Rotterdam and the Stichting Trombosedienst & Artsenlaboratorium Rijnmond (STAR-MDC), Rotterdam. We gratefully acknowledge the contribution of children and parents, general practitioners, hospitals, midwives and pharmacies in Rotterdam. The generation and management of GWAS genotype data for the Generation R Study were done at the Genetic Laboratory of the Department of Internal Medicine, Erasmus MC, The Netherlands. We would like to thank Karol Estrada, Dr. Tobias A. Knoch, Anis Abuseiris, Luc V. de Zeeuw, and Rob de Graaf, for their help in creating GRIMP, BigGRID, MediGRID, and Services@MediGRID/D-Grid, (funded by the German Bundesministerium fuer Forschung und Technology; grants 01 AK 803 A-H, 01 IG 07015 G) for access to their grid computing resources. We thank Mila Jhamai, Manoushka Ganesh, Pascal Arp, Marijn Verkerk, Lizbeth Herrera and Marjolein Peters for their help in creating, managing and QC of the GWAS database. Also, we thank Karol Estrada for their support in creation and analysis of imputed data.

**GINIplus & LISA**We thank all families for participation in the studies and the LISA and GINIplus study teams for their excellent work. The GINIplus study team wishes to acknowledge the following: Helmholtz Zentrum Muenchen - German Research Center for Environmental ealth, Institute of Epidemiology, Munich (Heinrich J, Brüske I, Schulz H, Standl M, Thiering E, Tiesler CMT, Chen C-M, Schnappinger M); Department of Pediatrics, Marien-Hospital, Wesel (Berdel D, von Berg A); Department of Pediatrics, Ludwig Maximilians University, Munich (Koletzko S); Department of Pediatrics, Technical University, Munich (Bauer CP, Hoffmann U); IUF – Leibniz Research Institute for Environmental Medicine, Düsseldorf (Schikowski T, Krämer U, Link E, Cramer C). The LISA study team wishes to acknowledge the following: Helmholtz Zentrum Muenchen - German Research Center for Environment and Health, Institute of Epidemiology, Neuherberg (Heinrich J, Brüske I, Schulz H, Standl M, Thiering E, Tiesler CMT, Che n C-M, Schnappinger M); Department of Pediatrics, Marien-Hospital, Wesel (von Berg A); Bad Honnef (Schaaf B); UFZ-Centre for Environmental Research Leipzig-Halle, Department of Environmental Immunology (Herberth G, Lehmann I); Department of Pediatrics, Technical University, Munich (Bauer CP, Hoffman U). **GOYA (Male)**We thank all the participants of the study.

**Helsinki Birth Cohort Study (HBCS)**The Helsinki Birth Cohort Study (HBCS/HBCS 1934-44) thanks Professor David Barker and Tom Forsen. The DNA extraction, sample quality control, biobank up-keep and aliquotting were performed at the National Institute for Health and Welfare, Helsinki, Finland.

**INfancia y Medio Ambiente [Environment and Childhood] (INMA) Project- Gipuzkoa subcohort, Menorca subcohort, and Sabadell and Valencia subcohort**The authors would like to thank all the participants for their generous collaboration. A full roster of the INMA Project Investigators can be found at http://www.proyectoinma.org/presentacion-inma/listado-investigadores/en_listado-investigadores.html. **LIFE-Child**We thank all children and families who participated in the studies. We gratefully appreciate the help of the study nurses, technical assistants and physicians who performed the clinical examinations and data collection.

**MAAS**We would like to thank the children and their parents for their continued support and enthusiasm. We greatly appreciate the commitment they have given to the project. We would also like to acknowledge the hard work and dedication of the study team (post-doctoral scientists, research fellows, nurses, physiologists, technicians and clerical staff).

**Northern Finnish Birth Cohort Study 1966 (NFBC66) and 1986 (NFBC86)**We thank the late professor Paula Rantakallio (launch of NFBC1966), the participants in the 31- and 46-years-old study and the NFBC project center. We thank professor AnnaLiisa Hartikainen (launch of NFBC1986), the participants in the study and the NFBC project center.

**Norwegian Mother, Father and Child (MoBa) Cohort Study**We are grateful to all the participating families in Norway who take part in this ongoing cohort study. Researchers interested in using MoBa data must obtain approval from the Scientific Management Committee of MoBa and from the Regional Committee for Medical and Health Research Ethics for access to data and biological material.

**The Netherlands Twin Register** **(NTR)**We very warmly thank all participants in the study. Netherlands Twin Register (NTR) is a longitudinal study of health and lifestyle of twins and their families across the Netherlands.

**The Physical Activity and Nutrition in Children (PANIC) Study**We are grateful for children, adolescents, and their families who have participated in The PANIC Study since the pilot study in 2006. We also thank all researchers and auxiliary personnel who have in many ways carried out The PANIC Study.

**Prevention and incidence of asthma and mite allergy birth cohort study - (PIAMA)**The study team gratefully acknowledges the participants in the PIAMA birth cohort study, and all coworkers who helped conducting the medical examinations, field work and data management.

**SCOOP**We are indebted to the patients and families involved in the Genetics of Obesity Study (GOOS) cohort. We also thank the staff of the NIHR Primary Care Research Network, the GPs, Physicians and nurses involved in identifying and recruiting participants. **SKOT I**The authors wish to thank all children and parents that were part of the SKOT I study.
 **Special Turku Coronary Risk Factor Intervention Project (STRIP)**We thank all the study participants and their families. The study was approved by the Joint Commission on Ethics of the Turku University and the Turku University Central Hospital. Informed consent was obtained from all parents at the beginning of the trial and from the children at 15 and 18 years of age. 
 **TEENS of Attica: Genes and Environment Study (TEENAGE)**We would like to thank all study participants and their families as well as all volunteers for their contribution in this study. We thank the following staff from the Sample Management and Genotyping Facilities at the Wellcome Trust Sanger Institute for sample preparation, quality control and genotyping: Dave Jones, Doug Simpkin, Emma Gray, Hannah Blackburn, Sarah Edkins.

**TEDS**We gratefully acknowledge the ongoing contribution of the participants in the Twins Early Development Study (TEDS) and their families. **TDCOB (cases and controls)**The Danish Childhood Obesity Biobank would like to thank all children, youths, and their families for participation in the studies and thus providing opportunities to detect novel insights in regards to childhood obesity.

**Tracking Adolescents' Individual Lives Survey (TRAILS)**We are grateful to everyone who participated in this research or worked on this project to make it possible. Statistical analyses were carried out on the Genetic Cluster Computer (<http://www.geneticcluster.org>) hosted by SURFsara and financially supported by the Netherlands Scientific Organization (NWO 480-05-003, PI: Posthuma) along with a supplement from the Dutch Brain Foundation and the VU University Amsterdam.

**The Raine Study**The Raine study would like to acknowledge the continued contribution of Raine Study participants and their families, Raine Study team for cohort coordination and data collection, NHMRC for long term funding over last 30 years, The University of Western Australia, Curtin University, Women and Infants Research Foundation, Telethon Kids Institute, Edith Cowan University, Murdoch University, The University of Notre Dame Australia, and The Raine Medical Research Foundation for providing funding for Core Management of the Raine Study. We also like to acknowledge The University of Western Australia (Division of Obstetrics and Gynaecology, King Edward Memorial Hospital and Medical School, Royal Perth Hospital), and Telethon Kids Institute for providing in-kind support for the storage and curation of biological samples, and Pawsey Supercomputing Centre with funding from Australian Government and the Government of Western Australia for providing computation resource to carry out analyses required.

**Study-specific funding information
ABCD study**

The ABCD study has been supported by grants from The Netherlands Organisation for Health Research and Development (ZonMW) and The Netherlands Heart Foundation. Genotyping was funded by the BBMRI-NL grant CP2013-50. Dr M.H. Zafarmand was supported by BBMRI-NL (CP2013-50). Dr. T.G.M. Vrijkotte was supported by ZonMW (TOP 40–00812–98–11010).

**Avon Longitudinal Study of Parents and Children (ALSPAC)**The UK Medical Research Council and Wellcome (Grant ref: 102215/2/13/2) and the University of Bristol provide core support for ALSPAC. A comprehensive list of grants funding is available on the ALSPAC website (http://www.bristol.ac.uk/alspac/external/documents/grant-acknowledgements.pdf); This research was specifically funded by Wellcome Trust and MRC (Grant ref: 076467/Z/05/Z). GWAS data was generated by Sample Logistics and Genotyping Facilities at Wellcome Sanger Institute and LabCorp (Laboratory Corporation of America) using support from 23andMe.

**BMDCS**Special thanks to the children and their families who participated in the BMDCS. Funded by NIH Grants: R01 HD058886 & UL1TR000003. D.L.C was supported by the American Diabetes Association Grant 1-17-PDF-077. S.F.A.G. is supported by the Daniel B. Burke Chair for Diabetes Research.

**BRain dEvelopment and Air polluTion ultrafine particles in scHool childrEn Project (BREATHE)**The research leading to these results has received funding from the European Research Council under the ERC Grant Agreement number 268479 – the BREATHE project. ISGlobal is a member of the CERCA Programme, Generalitat de Catalunya.

**British 1958 Birth Cohort (1958BC-WTCCC and 1958BC-T1DGC)**This work made use of data and samples generated by the 1958 Birth Cohort (NCDS) , which is managed by the Centre for Longitudinal Studies  at the UCL Institute of Education. The authors are deeply grateful to the 1958 birth cohort participants for their longstanding commitment and support, and to all staff for cohort coordination and data collection. The management of the 1958 Birth Cohort is funded by the Economic and Social Research Council (grant number ES/M001660/1). Access to these resources was enabled via the 58READIE Project funded by Wellcome Trust and Medical Research Council (grant numbers WT095219MA and G1001799). 1958 Birth Cohort data collection was funded by the Medical Research Council grant G0000934 and the Wellcome Trust grant 068545/Z/02. Genotyping for the B58C-WTCCC subset was funded by the Wellcome Trust grant 076113/B/04/Z.

**Cardiovascular Risk in Young Finns Study (YFS)**The Young Finns Study has been financially supported by the Academy of Finland: grants 286284, 134309 (Eye), 126925, 121584, 124282, 129378 (Salve), 117787 (Gendi), and 41071 (Skidi); the Social Insurance Institution of Finland; Competitive State Research Financing of the Expert Responsibility area of Kuopio, Tampere and Turku University Hospitals (grant X51001); Juho Vainio Foundation; Paavo Nurmi Foundation; Finnish Foundation for Cardiovascular Research ; Finnish Cultural Foundation; The Sigrid Juselius Foundation; Tampere Tuberculosis Foundation; Emil Aaltonen Foundation; Yrjö Jahnsson Foundation; Signe and Ane Gyllenberg Foundation; Diabetes Research Foundation of Finnish Diabetes Association; and EU Horizon 2020 (grant 755320 for TAXINOMISIS); and European Research Council (grant 742927 for MULTIEPIGEN project); Tampere University Hospital Supporting Foundation.
 **Children’s Hospital of Philadelphia (CHOP)**CHOP was financially supported by an Institute Development Award from the Children’s Hospital of Philadelphia, a Research Development Award from the Cotswold Foundation, NIH grant R01 HD056465, The Children’s Hospital of Philadelphia Endowed Chair for Genomics Research (HH) and the Daniel B. Burke Endowed Chair for Diabetes Research (SFAG).

**CHOP (Europe)**The research of the CHOP study reported herein was partially supported by the Commission of the European Community, specific RTD Programme “Quality of Life and Management of Living Resources,” within the 5th Framework Programme (research grant nos. QLRT-2001-00389 and QLK1-CT-2002-30582); the 6th Framework Programme contract no. 007036 (FP6-007036); the European Union’s Seventh Framework Programme Project EarlyNutrition under grant agreement no. 289346 (FP7-289346), the Horizon 2020 research and innovation programme DynaHEALTH (no. 633595) and the European Research Council Advanced Grant META-GROWTH (ERC-2012-AdG – no. 322605). Additional support from the German Ministry of Education and Research, Berlin (Grant Nr. 01 GI 0825) and the University of Munich Innovative Research Priority Project MC-Health is gratefully acknowledged. This manuscript does not necessarily reflect the views of the Commission and in no way anticipates the future policy in this area.

**Copenhagen Study on Asthma in Childhood 2000 birth cohort (COPSAC2000)**COPSAC greatly acknowledges the private and public research funding allocated to COPSAC and listed on www.copsac.com, with special thanks to The Lundbeck Foundation (Grant no R16-A1694); Ministry of Health (Grant no 903516); Danish Council for Strategic Research (Grant no 0603-00280B); The Danish Council for Independent Research and The Capital Region Research Foundation as core supporters. No pharmaceutical company was involved in the study.

**Copenhagen Study on Asthma in Childhood 2010 birth cohort (COPSAC2010)**COPSAC greatly acknowledges the private and public research funding allocated to COPSAC and listed on www.copsac.com, with special thanks to The Lundbeck Foundation (Grant no R16-A1694); Ministry of Health (Grant no 903516); Danish Council for Strategic Research (Grant no 0603-00280B); The Danish Council for Independent Research and The Capital Region Research Foundation as core supporters. No pharmaceutical company was involved in the study.

**Danish National Birth Cohort (DNBC) - GOYA offspring**The Danish National Research Foundation has established the Danish Epidemiology Science Centre that initiated and created the Danish National Birth Cohort (DNBC). The cohort is furthermore a result of a major grant from this foundation. Additional support for the Danish National Birth Cohort is obtained from the Pharmacy Foundation, the Egmont Foundation, the March of Dimes Birth Defects Foundation, the Augustinus Foundation, and the Health Foundation. The DNBC biobank is a part of the Danish National Biobank resource, which is supported by the Novo Nordisk Foundation. GOYA and GOYA-offspring are nested within the DNBC. The studies are conducted in collaboration with the MRC Integrative Epidemiology Unit at the University of Bristol (MC_UU_00011/1-9). The genotyping for DNBC-GOYA study was funded by the Wellcome Trust (WT 084762) and the genotyping in the GOYA-offspring has been financed by the Novo Nordisk Foundation Center for Basic Metabolic Research. The project was supported by a grant from the Danish Council for Independent Research (DFF-7016-00381).

**DNBC-PTB**Support for the Danish National Birth Cohort (DNBC) was obtained from the Danish National Research Foundation, the Danish Pharmacists’ Fund, the Egmont Foundation, the March of Dimes Birth Defects Foundation, the Augustinus Foundation and the Health Fund of the Danish Health Insurance Societies. The DNBC biobank is a part of the Danish National Biobank resource, which is supported by the Novo Nordisk Foundation. The DNBC preterm study is a nested study within the DNBC. The generation of GWAS genotype data for the DNBC preterm sample was carried out within the Gene Environment Association Studies (GENEVA) consortium with funding provided through the National Institutes of Health's Genes, Environment, and Health Initiative (U01HG004423; U01HG004446; U01HG004438).

**EDEN**Support for the EDEN study was provided by the following organisations: Fondation pour la Recherche Médicale, French Ministry of Research, Institut Fédératif de Recherche and Cohort Program, INSERM Nutrition Research Program, French Ministry of Health Perinatal Program, French Agency for Environment Security (AFFSET), French National Institute for Population Health Surveillance (INVS), Paris-Sud University, French National Institute for Health Education (INPES), Nestlé, Mutuelle Généale de l'éducation Nationale, French Speaking Association for the Study of Diabetes and Metabolism (Alfediam), National Agency for Research (ANR nonthematic program), and National Institute for Research in Public Health (IRESP TGIR Cohorte Santé 2008 Program).

**EFSOCH**The Exeter Family Study of Childhood Health (EFSOCH) was supported by South West NHS Research and Development, Exeter NHS Research and Development, the Darlington Trust and the Peninsula National Institute of Health Research (NIHR) Clinical Research Facility at the University of Exeter. The opinions given in this paper do not necessarily represent those of NIHR, the NHS or the Department of Health. Genotyping of the EFSOCH study samples was funded by the Welcome Trust and Royal Society grant WT104150

**FAMILY**The FAMILY study was funded by the Hamilton Health Science Foundation, the Canadian Institutes of Health Research and by Heart & Stroke Foundation of Ontario as well as additional grants from the PHRI internal funds. D.M. is supported by a Canada Research Chair in Genetics of Obesity.
 **French Young**This work was supported by grants from the Agence Nationale de la Recherche, the Conseil Régional Nord-Pas de Calais as part of Fonds Européen de Développement Economique et Regional, Genome Quebec as part of Genome Canada and the UK MRC.

**Generation R Study (Generation R)**The general design of Generation RStudy is made possible by financial support from the Erasmus Medical Center, Rotterdam, the Erasmus University Rotterdam, the Netherlands Organization for Health Research and Development (ZonMw), the Netherlands Organisation for Scientific Research (NWO), the Ministry of Health, Welfare and Sport and the Ministry of Youth and Families. This project received funding from the European Union’s Horizon 2020 Research and Innovation Programme under grant agreements 633595 (DynaHEALTH) and 733206 (LifeCycle).

**GINIplus**

The GINIplus study was mainly supported for the first 3 years of the Federal Ministry for Education, Science, Research and Technology (interventional arm) and Helmholtz Zentrum Munich (former GSF) (observational arm). The 4 year, 6 year, 10 year and 15 year follow-up examinations of the GINIplus study were covered from the respective budgets of the 5 study centres (Helmholtz Zentrum Munich (former GSF), Research Institute at Marien-Hospital Wesel, LMU Munich, TU Munich and from 6 years onwards also from IUF - Leibniz Research-Institute for Environmental Medicine at the University of Düsseldorf) and a grant from the Federal Ministry for Environment (IUF Düsseldorf, FKZ 20462296). Further, the 15 year follow-up examination of the GINIplus study was supported by the Commission of the European Communities, the 7th Framework Program: MeDALL project, and as well by the companies Mead Johnson and Nestlé.

**LISA**

The LISA study was mainly supported by grants from the Federal Ministry for Education, Science, Research and Technology and in addition from Helmholtz Zentrum Munich (former GSF), Helmholtz Centre for Environmental Research - UFZ, Leipzig, Research Institute at Marien-Hospital Wesel, Pediatric Practice, Bad Honnef for the first 2 years. The 4 year, 6 year, 10 year and 15 year follow-up examinations of the LISA study were covered from the respective budgets of the involved partners (Helmholtz Zentrum Munich (former GSF), Helmholtz Centre for Environmental Research - UFZ, Leipzig, Research Institute at Marien-Hospital Wesel, Pediatric Practice, Bad Honnef, IUF – Leibniz-Research Institute for Environmental Medicine at the University of Düsseldorf) and in addition by a grant from the Federal Ministry for Environment (IUF Düsseldorf, FKZ 20462296). Further, the 15 year follow-up examination of the LISA study was supported by the Commission of the European Communities, the 7th Framework Program: MeDALL project.

**GOYA (Male)**GOYA study was conducted as part of the activities of the Gene-diet Interactions in Obesity project (GENDINOB, www.gendinob.dk) and the MRC Integrative Epidemiology Unit at the University of Bristol (MC_UU_00011/1-9).
 **Helsinki Birth Cohort Study (HBCS)**We thank all study participants as well as everybody involved in the Helsinki Birth Cohort Study. Helsinki Birth Cohort Study has been supported by grants from the Academy of Finland (JGE grant no. 129369, 129907, 135072, 129255 and 126775), the Finnish Diabetes Research Society, Samfundet Folkhälsan, Novo Nordisk Foundation, Finska Läkaresällskapet, Juho Vainio Foundation, Signe and Ane Gyllenberg Foundation, Liv och Hälsa, University of Helsinki and Ministry of Education.

**INfancia y Medio Ambiente [Environment and Childhood] (INMA) Project- Gipuzkoa subcohort**This study was funded by Consortium for Biomedical Research in Epidemiology and Public Health (CIBER en Epidemiologia y Salud Publica-CIBERESP, Group 28), Instituto de Salud Carlos III (FIS-PI06/0867, FIS-PI09/00090, FIS-PI13/02187 and FIS-PI18/01142, co-funded by ERDF, “A way to make Europe”), Department of Health of the Basque Government (2005111093, 2009111069, 2013111089, 2015111065 and 2018111086), and the Provincial Government of Gipuzkoa (DFG06/002, DFG08/001 and DFG15/221) and annual agreements with the municipalities of the study area (Zumarraga, Urretxu, Legazpi, Azkoitia, Azpeitia and Beasain).

**INfancia y Medio Ambiente [Environment and Childhood] (INMA) Project- Menorca subcohort**This study was funded by grants from Instituto de Salud Carlos III (CB06/02/0041, G03/176, FIS PI041436, PI081151, PI041705, and PS09/00432, FIS-FEDER 03/1615, 04/1509, 04/1112, 04/1931 , 05/1079, 05/1052, 06/1213, 07/0314, and 09/02647), Fundació La Marató de TV3 (090430), and Fundación Roger Torné. ISGlobal is a member of the CERCA Programme, Generalitat de Catalunya. **INfancia y Medio Ambiente [Environment and Childhood] (INMA) Project- Sabadell and Valencia subcohort**This study was funded by grants from Instituto de Salud Carlos III (CB06/02/0041, G03/176, FIS PI041436, PI081151, PI041705, and PS09/00432, FIS-FEDER 03/1615, 04/1509, 04/1112, 04/1931 , 05/1079, 05/1052, 06/1213, 07/0314, and 09/02647), Fundació La Marató de TV3 (090430), Generalitat de Catalunya-CIRIT (1999SGR 00241), Conselleria de Sanitat Generalitat Valenciana, and Fundación Roger Torné. ISGlobal is a member of the CERCA Programme, Generalitat de Catalunya.
 **LIFE-Child**LIFE-Child is funded by the LIFE – Leipzig Research Center for Civilization Diseases, University of Leipzig. LIFE is funded by means of the European Union, by means of the European Social Fund (ESF), by the European Regional Development Fund ERDF), and by means of the Free State of Saxony within the framework of the excellence initiative. Genotyping was supported within the scope of the Beta-JUDO project funded by the European Community’s Seventh Framework Programme (FP7/2007-2013 n° 279153). M.S. and A.K. are supported by Federal Ministry of Education and Research (BMBF), Germany, FKZ: 01EO1001 (IFB AdiposityDiseases ADI K7-113), and A.K. is supported by German Research Foundation (DFG) for the Clinical Research Center “Obesity Mechanisms” CRC1052/ C05, and H.K. is supported by the Leipzig Research Center for Civilization Diseases (LIFE).

**MAAS**MAAS was supported by the Asthma UK Grants No 301 (1995-1998), No 362 (1998-2001), No 01/012 (2001-2004), No 04/014 (2004-2007), the Medical Research Council UK (Grants G0601361, MR/K002449/1 and MR/L012693/1), the BMA James Trust, The Moulton Charitable Foundation (2004-current) the North West Lung Centre Charity (1995-current) and National Institute for Health Research Respiratory Clinical Research Facility at Manchester University NHS Foundation Trust. Angela Simpson and Clare Murray are supported by the NIHR Manchester Biomedical Research Centre. The views expressed in this publication are those of the authors and not necessarily those of the NHS, the National Institute for Health Research or the Department of Health.

**Northern Finnish Birth Cohort Study 1966 (NFBC66) and 1986 (NFBC1986)**

The Northern Finland Birth Cohort (NFBC) Research program, including the study of the NFBC 1966 and 1986 studies, received financial support from Academy of Finland (1114194, 24300796), NHLBI grant 5R01HL087679 through the STAMPEED program (1RL1MH083268-01), ENGAGE project and grant agreement HEALTH-F4-2007-201413, the Medical Research Council (grant G0500539, centre grant G0600705, PrevMetSyn), and the Wellcome Trust (project grant GR069224), UK. The program is currently being funded by the H2020-633595 DynaHEALTH action and Academy of Finland EGEA-project.

**Norwegian Mother, Father and Child (MoBa) Cohort Study**The Norwegian Mother, Father and Child Cohort Study is supported by the Norwegian Ministry of Health and Care Services and the Ministry of Education and Research. We are grateful to all the participating families in Norway who take part in this on-going cohort study. We thank the Norwegian Institute of Public Health (NIPH) for generating high-quality genomic data. This research is part of the HARVEST collaboration, supported by the Research Council of Norway (#229624). We further thank the Center for Diabetes Research, the University of Bergen for providing genotype data and performing quality control and imputation of the data funded by the ERC AdG project SELECTionPREDISPOSED, Stiftelsen Kristian Gerhard Jebsen, Trond Mohn Foundation, the Research Council of Norway, the Novo Nordisk Foundation, the University of Bergen, and the Western Norway health Authorities (Helse Vest). We also received contribution from Jane and Dan Olsson Foundations (Gothenburg, Sweden), Swedish Medical Research Council (2015-02559), Norwegian Research Council/FUGE (grant no. 151918/S10; FRI-MEDBIO 249779) and March of Dimes (21-FY16-121), Swedish government grants to researchers in the public health service (ALFGBG-507701). Researchers interested in using MoBa data must obtain approval from the Scientific Management Committee of MoBa and from the Regional Committee for Medical and Health Research Ethics for access to data and biological material.

**The Netherlands Twin Register** **(NTR)**This study makes use of data in the Netherlands Twin Register (NTR). We acknowledge support from the BBMRI - NL Consortium, a research infrastructure financed by the Dutch government (NWO, no. 184.021.007 and 184.033.111), the Genetic Association Information Network (GAIN) of the Foundation for the National Institutes of Health, Rutgers University Cell and DNA Repository (NIMH U24 MH068457-06), the Avera Institute, Sioux Falls (USA) and the National Institutes of Health (NIH R01 HD042157-01A1, MH081802, Grand Opportunity grants 1RC2 MH089951 and 1RC2 MH089995), the European Research Council (ERC-230374), the European Union Seventh Framework Program (FP7/2007-2013): ACTION Consortium (Aggression in Children: Unravelling gene-environment interplay to inform Treatment and InterventiON strategies; grant number 602768) and grant NWO 480-15-001/674: Netherlands Twin Registry Repository: researching the interplay between genome and environment.

**The Physical Activity and Nutrition in Children (PANIC) Study**The PANIC Study has financially been supported by grants from Ministry of Education and Culture of Finland, Ministry of Social Affairs and Health of Finland, Research Committee of the Kuopio University Hospital Catchment Area (State Research Funding), Finnish Innovation Fund Sitra, Social Insurance Institution of Finland, Finnish Cultural Foundation, Foundation for Paediatric Research, Diabetes Research Foundation in Finland, Finnish Foundation for Cardiovascular Research, Juho Vainio Foundation, Paavo Nurmi Foundation, Yrjö Jahnsson Foundation, and the city of Kuopio.

**Prevention and incidence of asthma and mite allergy birth cohort study - (PIAMA)**The PIAMA study was funded by grants from the Dutch Asthma Foundation (grant 3.4.01.26, 3.2.06.022, 3.4.09.081 and 3.2.10.085CO), the ZON-MW Netherlands Organization for Health Research and Development (grant 912-03-031), the Stichting Astmabestrijding and the Ministry of the Environment. Genome-wide genotyping was funded by the European Commission as part of GABRIEL (A multidisciplinary study to identify the genetic and environmental causes of asthma in the European Community) contract number 018996 under the Integrated Program LSH-2004-1.2.5-1 Post genomic approaches to understand the molecular basis of asthma aiming at a preventive or therapeutic control and a Grant from BBMRI-NL (CP 29).

**SCOOP**

I.S.F. was supported by the European Research Council, Wellcome Trust (098497/Z/12/Z), Medical Research Council (MRC_MC_UU_12012/5), the NIHR Cambridge Biomedical Research Centre, the Botnar Foundation, the Bernard Wolfe Health Neuroscience Endowment and the European Community’s Seventh Framework Programme (FP7/2007-2013) project Beta-JUDO n°279153. IB acknowledges funding from Wellcome (WT206194).
 **SKOT I**The SKOT I study was supported by grants from The Danish Directorate for Food, Fisheries and Agri Business as part of the ‘Complementary and young child feeding (CYCF) – impact on short- and long-term development and health’ project and partly by contributions from the research program ‘Governing Obesity’ by the University of Copenhagen Excellence Program for Interdisciplinary Research (www.go.ku.dk).

**Special Turku Coronary Risk Factor Intervention Project (STRIP)**STRIP was supported by the Academy of Finland (grants 206374, 294834, 251360, and 275595), the Juho Vainio Foundation, the Finnish Foundation for Cardiovascular Research, the Finnish Ministry of Education and Culture, the Finnish Cultural Foundation, the Sigrid Juselius Foundation, Special Governmental grants for Health Sciences Research (Turku University Hospital), the Yrjö Jahnsson Foundation, and the Turku University Foundation.
 **TEENS of Attica: Genes and Environment Study (TEENAGE)**TEENAGE study has been co-financed by the European Union (European Social Fund—ESF) and Greek national funds through the Operational Program “Education and Lifelong Learning” of the National Strategic Reference Framework (NSRF)—Research Funding Program: Heracleitus II. Investing in knowledge society through the European Social Fund. This work was funded by the Wellcome Trust (098051). We would like to thank all study participants and their families as well as all volunteers for their contribution in this study.

**TEDS**We gratefully acknowledge the ongoing contribution of the participants in the Twins Early Development Study (TEDS) and their families. TEDS is supported by a program grant to RP from the UK Medical Research Council (MR/M021475/1 and previously G0901245), with additional support from the US National Institutes of Health (AG046938). The research leading to these results has also received funding from the European Research Council under the European Union's Seventh Framework Programme (FP7/2007-2013)/ grant agreement n° 602768 and ERC grant agreement n° 295366. RP is supported by a Medical Research Council Professorship award (G19/2). SS is supported by the MRC/IoPPN Excellence Award and by the US National Institutes of Health (AG046938). High performance computing facilities were funded with capital equipment grants from the GSTT Charity (TR130505) and Maudsley Charity (980).
 **TDCOB (cases and controls)**TDCOB received supports from the Danish Innovation Foundation and The Region Zealand Health and Medical Research Foundation. This study was partly supported by grants from the Program Committee for Individuals, Disease and Society of the Danish Innovation Foundation (grant numbers: 0603-00484B and 0603-00457B) and was part of the research activities in The Impact of our Genomes on Individual Treatment Response in Obese Children (TARGET, www.target.ku.dk) and the Indo-Danish bi-lateral project, Genetics and Systems Biology of Childhood Obesity in India and Denmark (BioChild, www.biochild.ku.dk). This study received further funding from The Novo Nordisk Foundation (grant number NNF15OC0016544 and NNF15OC0016692). The Novo Nordisk Foundation Center for Basic Metabolic Research is an independent Research Center at the University of Copenhagen and was partially funded by an unrestricted donation from the Novo Nordisk Foundation (NNF18CC0034900).
 **Tracking Adolescents' Individual Lives Survey (TRAILS)**TRAILS has been financially supported by various grants from the Netherlands Organization for Scientific Research NWO (Medical Research Council program grant GB-MW 940-38-011; ZonMW Brainpower grant 100-001-004; ZonMw Risk Behavior and Dependence grants 60-60600-97-118; ZonMw Culture and Health grant 261-98-710; Social Sciences Council medium-sized investment grants GB-MaGW 480-01-006 and GB-MaGW 480-07-001; Social Sciences Council project grants GB-MaGW 452-04-314 and GB-MaGW 452-06-004; NWO large-sized investment grant 175.010.2003.005; NWO Longitudinal Survey and Panel Funding 481-08-013 and 481-11-001; NWO Vici 016.130.002 and 453-16-007/2735; NWO Gravitation 024.001.003), the Dutch Ministry of Justice (WODC), the European Science Foundation (EuroSTRESS project FP-006), the European Research Council (ERC-2017-STG-757364 en ERC-CoG-2015-681466), Biobanking and Biomolecular Resources Research Infrastructure BBMRI-NL (CP 32), the Gratama foundation, the Jan Dekker foundation, the participating universities, and Accare Centre for Child and Adolescent Psychiatry. **The Raine Study**The Raine Study was supported by the National Health and Medical Research Council of Australia (grant numbers 572613, 403981 and 003209) and the Canadian Institutes of Health Research (grant number MOP-82893).

The funders had no role in study design, data collection and analysis, data interpretation, decision to publish, or preparation of the manuscript.

**References**

1 Boyd, A. *et al.* Cohort Profile: the 'children of the 90s'--the index offspring of the Avon Longitudinal Study of Parents and Children. *Int J Epidemiol* **42**, 111-127 (2013).

2 Fraser, A. *et al.* Cohort Profile: the Avon Longitudinal Study of Parents and Children: ALSPAC mothers cohort. *Int J Epidemiol* **42**, 97-110 (2013).

3 Stergiakouli, E. *et al.* Genome-wide association study of height-adjusted BMI in childhood identifies functional variant in ADCY3. *Obesity (Silver Spring)* **22**, 2252-2259 (2014).

4 Kalkwarf, H. J. *et al.* The bone mineral density in childhood study: bone mineral content and density according to age, sex, and race. *J Clin Endocrinol Metab* **92**, 2087-2099 (2007).

5 Zemel, B. S. *et al.* Revised reference curves for bone mineral content and areal bone mineral density according to age and sex for black and non-black children: results of the bone mineral density in childhood study. *J Clin Endocrinol Metab* **96**, 3160-3169 (2011).

6 Power, C. & Elliott, J. Cohort profile: 1958 British birth cohort (National Child Development Study). *Int J Epidemiol* **35**, 34-41 (2006).

7 Sawcer, S. *et al.* Genetic risk and a primary role for cell-mediated immune mechanisms in multiple sclerosis. *Nature* **476**, 214-219 (2011).

8 Barrett, J. C. *et al.* Genome-wide association study and meta-analysis find that over 40 loci affect risk of type 1 diabetes. *Nat Genet* **41**, 703-707 (2009).

9 Raitakari, O. T. *et al.* Cohort profile: the cardiovascular risk in Young Finns Study. *Int J Epidemiol* **37**, 1220-1226 (2008).

10 Howie, B. N., Donnelly, P. & Marchini, J. A flexible and accurate genotype imputation method for the next generation of genome-wide association studies. *PLoS Genet* **5**, e1000529 (2009).

11 Howie, B., Fuchsberger, C., Stephens, M., Marchini, J. & Abecasis, G. R. Fast and accurate genotype imputation in genome-wide association studies through pre-phasing. *Nat Genet* **44**, 955-959 (2012).

12 Koletzko, B. *et al.* Lower protein in infant formula is associated with lower weight up to age 2 y: a randomized clinical trial. *Am J Clin Nutr* **89**, 1836-1845 (2009).

13 Weber, M. *et al.* Lower protein content in infant formula reduces BMI and obesity risk at school age: follow-up of a randomized trial. *Am J Clin Nutr* **99**, 1041-1051 (2014).

14 Rzehak, P. *et al.* DNA-Methylation and Body Composition in Preschool Children: Epigenome-Wide-Analysis in the European Childhood Obesity Project (CHOP)-Study. *Sci Rep* **7**, 14349 (2017).

15 Rzehak, P. *et al.* Maternal Smoking during Pregnancy and DNA-Methylation in Children at Age 5.5 Years: Epigenome-Wide-Analysis in the European Childhood Obesity Project (CHOP)-Study. *PLoS One* **11**, e0155554 (2016).

16 Kirchberg, F. F. *et al.* Dietary protein intake affects amino acid and acylcarnitine metabolism in infants aged 6 months. *J Clin Endocrinol Metab* **100**, 149-158 (2015).

17 Zhan, X., Hu, Y., Li, B., Abecasis, G. R. & Liu, D. J. RVTESTS: an efficient and comprehensive tool for rare variant association analysis using sequence data. *Bioinformatics* **32**, 1423-1426 (2016).

18 Bisgaard, H. The Copenhagen Prospective Study on Asthma in Childhood (COPSAC): design, rationale, and baseline data from a longitudinal birth cohort study. *Ann Allergy Asthma Immunol* **93**, 381-389 (2004).

19 Bisgaard, H. *et al.* Deep phenotyping of the unselected COPSAC2010 birth cohort study. *Clin Exp Allergy* **43**, 1384-1394 (2013).

20 Waage, J. *et al.* Genome-wide association and HLA fine-mapping studies identify risk loci and genetic pathways underlying allergic rhinitis. *Nat Genet* **50**, 1072-1080 (2018).

21 Olsen, J. *et al.* The Danish National Birth Cohort--its background, structure and aim. *Scand J Public Health* **29**, 300-307 (2001).

22 Paternoster, L. *et al.* Genome-wide population-based association study of extremely overweight young adults--the GOYA study. *PLoS One* **6**, e24303 (2011).

23 Knight, B., Shields, B. M. & Hattersley, A. T. The Exeter Family Study of Childhood Health (EFSOCH): study protocol and methodology. *Paediatr Perinat Epidemiol* **20**, 172-179 (2006).

24 Knight, B. *et al.* The impact of maternal glycemia and obesity on early postnatal growth in a nondiabetic Caucasian population. *Diabetes Care* **30**, 777-783 (2007).

25 Hughes, A. E. *et al.* Fetal Genotype and Maternal Glucose Have Independent and Additive Effects on Birth Weight. *Diabetes* **67**, 1024-1029 (2018).

26 Abraham, G. & Inouye, M. Fast principal component analysis of large-scale genome-wide data. *PLoS One* **9**, e93766 (2014).

27 Manichaikul, A. *et al.* Robust relationship inference in genome-wide association studies. *Bioinformatics* **26**, 2867-2873 (2010).

28 Morrison, K. M. *et al.* The Family Atherosclerosis Monitoring In earLY life (FAMILY) study: rationale, design, and baseline data of a study examining the early determinants of atherosclerosis. *Am Heart J* **158**, 533-539 (2009).

29 Meyre, D. *et al.* Genome-wide association study for early-onset and morbid adult obesity identifies three new risk loci in European populations. *Nat Genet* **41**, 157-159 (2009).

30 Visvikis-Siest, S. & Siest, G. The STANISLAS Cohort: a 10-year follow-up of supposed healthy families. Gene-environment interactions, reference values and evaluation of biomarkers in prevention of cardiovascular diseases. *Clin Chem Lab Med* **46**, 733-747 (2008).

31 Lafay, L. *et al.* Determinants and nature of dietary underreporting in a free-living population: the Fleurbaix Laventie Ville Sante (FLVS) Study. *Int J Obes Relat Metab Disord* **21**, 567-573 (1997).

32 Kooijman, M. N. *et al.* The Generation R Study: design and cohort update 2017. *Eur J Epidemiol* **31**, 1243-1264 (2016).

33 Li, Y., Willer, C., Sanna, S. & Abecasis, G. Genotype imputation. *Annu Rev Genomics Hum Genet* **10**, 387-406 (2009).

34 Li, Y., Willer, C. J., Ding, J., Scheet, P. & Abecasis, G. R. MaCH: using sequence and genotype data to estimate haplotypes and unobserved genotypes. *Genet Epidemiol* **34**, 816-834 (2010).

35 Rzehak, P. *et al.* Period-specific growth, overweight and modification by breastfeeding in the GINI and LISA birth cohorts up to age 6 years. *Eur J Epidemiol* **24**, 449-467 (2009).

36 Zutavern, A. *et al.* Timing of solid food introduction in relation to atopic dermatitis and atopic sensitization: results from a prospective birth cohort study. *Pediatrics* **117**, 401-411 (2006).

37 Guxens, M. *et al.* Cohort Profile: the INMA--INfancia y Medio Ambiente--(Environment and Childhood) Project. *Int J Epidemiol* **41**, 930-940 (2012).

38 Quante, M. *et al.* The LIFE child study: a life course approach to disease and health. *BMC Public Health* **12**, 1021 (2012).

39 Poulain, T. *et al.* The LIFE Child study: a population-based perinatal and pediatric cohort in Germany. *Eur J Epidemiol* **32**, 145-158 (2017).

40 Custovic, A. *et al.* The National Asthma Campaign Manchester Asthma and Allergy Study. *Pediatr Allergy Immunol* **13**, 32-37 (2002).

41 Lowe, L. A. *et al.* Wheeze phenotypes and lung function in preschool children. *Am J Respir Crit Care Med* **171**, 231-237 (2005).

42 Murray, C. S. *et al.* Lung function at one month of age as a risk factor for infant respiratory symptoms in a high risk population. *Thorax* **57**, 388-392 (2002).

43 Nicolaou, N. C. *et al.* Exhaled breath condensate pH and childhood asthma: unselected birth cohort study. *Am J Respir Crit Care Med* **174**, 254-259 (2006).

44 Nicolaou, N. C. *et al.* Day-care attendance, position in sibship, and early childhood wheezing: a population-based birth cohort study. *J Allergy Clin Immunol* **122**, 500-506 e505 (2008).

45 Wang, R. *et al.* Differing associations of BMI and body fat with asthma and lung function in children. *Pediatr Pulmonol* **49**, 1049-1057 (2014).

46 Rantakallio, P. Groups at risk in low birth weight infants and perinatal mortality. *Acta Paediatr Scand* **193**, Suppl 193:191+ (1969).

47 Sovio, U. *et al.* How do changes in body mass index in infancy and childhood associate with cardiometabolic profile in adulthood? Findings from the Northern Finland Birth Cohort 1966 Study. *Int J Obes (Lond)* **38**, 53-59 (2014).

48 Jarvelin, M. R. *et al.* Early life factors and blood pressure at age 31 years in the 1966 northern Finland birth cohort. *Hypertension* **44**, 838-846 (2004).

49 Magnus, P. *et al.* Cohort Profile Update: The Norwegian Mother and Child Cohort Study (MoBa). *Int J Epidemiol* **45**, 382-388 (2016).

50 Nilsen, R. M. *et al.* Self-selection and bias in a large prospective pregnancy cohort in Norway. *Paediatr Perinat Epidemiol* **23**, 597-608 (2009).

51 Paltiel L *et al*. The biobank of the Norwegian Mother and Child Cohort Study – present status. *Norsk Epidemiologi* 2014; **24** (1-2): 29-35 29.

52 Paltiel L *et al*. The biobank of the Norwegian Mother and Child Cohort Study – present status *Nor J Epidemiol* 2014; **24** (1-2): 29-35.

53 Boomsma, D. I., Orlebeke, J. F. & van Baal, G. C. The Dutch Twin Register: growth data on weight and height. *Behav Genet* **22**, 247-251 (1992).

54 van Beijsterveldt, C. E. *et al.* The Young Netherlands Twin Register (YNTR): longitudinal twin and family studies in over 70,000 children. *Twin Res Hum Genet* **16**, 252-267 (2013).

55 Estourgie-van Burk, G. F., Bartels, M., Boomsma, D. I. & Delemarre-van de Waal, H. A. Body size of twins compared with siblings and the general population: from birth to late adolescence. *J Pediatr* **156**, 586-591 (2010).

56 Scheet, P. *et al.* Twins, tissue, and time: an assessment of SNPs and CNVs. *Twin Res Hum Genet* **15**, 737-745 (2012).

57 Eloranta, A. M. *et al.* Dietary factors associated with overweight and body adiposity in Finnish children aged 6-8 years: the PANIC Study. *Int J Obes (Lond)* **36**, 950-955 (2012).

58 Brunekreef, B. *et al.* The prevention and incidence of asthma and mite allergy (PIAMA) birth cohort study: design and first results. *Pediatr Allergy Immunol* **13**, 55-60 (2002).

59 Moffatt, M. F. *et al.* A large-scale, consortium-based genomewide association study of asthma. *N Engl J Med* **363**, 1211-1221 (2010).

60 Bochukova, E. G. *et al.* Large, rare chromosomal deletions associated with severe early-onset obesity. *Nature* **463**, 666-670 (2010).

61 Huang, J. *et al.* Improved imputation of low-frequency and rare variants using the UK10K haplotype reference panel. *Nat Commun* **6**, 8111 (2015).

62 Riveros-McKay, F. *et al.* Genetic architecture of human thinness compared to severe obesity. *PLoS Genet* **15**, e1007603 (2019).

63 Madsen, A. L., Larnkjaer, A., Molgaard, C. & Michaelsen, K. F. IGF-I and IGFBP-3 in healthy 9 month old infants from the SKOT cohort: breastfeeding, diet, and later obesity. *Growth Horm IGF Res* **21**, 199-204 (2011).

64 Simell, O. *et al.* Cohort Profile: the STRIP Study (Special Turku Coronary Risk Factor Intervention Project), an Infancy-onset Dietary and Life-style Intervention Trial. *Int J Epidemiol* **38**, 650-655 (2009).

65 Lagstrom, H. *et al.* Growth patterns and obesity development in overweight or normal-weight 13-year-old adolescents: the STRIP study. *Pediatrics* **122**, e876-883 (2008).

66 Ntalla, I. *et al.* Body composition and eating behaviours in relation to dieting involvement in a sample of urban Greek adolescents from the TEENAGE (TEENs of Attica: Genes & Environment) study. *Public Health Nutr* **17**, 561-568 (2014).

67 Ntalla, I. *et al.* Replication of established common genetic variants for adult BMI and childhood obesity in Greek adolescents: the TEENAGE study. *Ann Hum Genet* **77**, 268-274 (2013).

68 Teo, Y. Y. *et al.* A genotype calling algorithm for the Illumina BeadArray platform. *Bioinformatics* **23**, 2741-2746 (2007).

69 Marchini, J. & Howie, B. Genotype imputation for genome-wide association studies. *Nat Rev Genet* **11**, 499-511 (2010).

70 Chang, C. C. *et al.* Second-generation PLINK: rising to the challenge of larger and richer datasets. *Gigascience* **4**, 7 (2015).

71 Purcell, S. *et al.* PLINK: a tool set for whole-genome association and population-based linkage analyses. *Am J Hum Genet* **81**, 559-575 (2007).

72 Li, H. A statistical framework for SNP calling, mutation discovery, association mapping and population genetical parameter estimation from sequencing data. *Bioinformatics* **27**, 2987-2993 (2011).

73 Patterson, N., Price, A. L. & Reich, D. Population structure and eigenanalysis. *PLoS Genet* **2**, e190 (2006).

74 Loh, P. R. *et al.* Reference-based phasing using the Haplotype Reference Consortium panel. *Nat Genet* **48**, 1443-1448 (2016).

75 Durbin, R. Efficient haplotype matching and storage using the positional Burrows-Wheeler transform (PBWT). *Bioinformatics* **30**, 1266-1272 (2014).

76 McCarthy, S. *et al.* A reference panel of 64,976 haplotypes for genotype imputation. *Nat Genet* **48**, 1279-1283 (2016).

77 Nysom, K., Molgaard, C., Hutchings, B. & Michaelsen, K. F. Body mass index of 0 to 45-y-old Danes: reference values and comparison with published European reference values. *Int J Obes Relat Metab Disord* **25**, 177-184 (2001).

78 Graae, A. S. *et al.* An adult-based insulin resistance genetic risk score associates with insulin resistance, metabolic traits and altered fat distribution in Danish children and adolescents who are overweight or obese. *Diabetologia* **61**, 1769-1779 (2018).

79 Oldehinkel, A. J. *et al.* Cohort Profile Update: the TRacking Adolescents' Individual Lives Survey (TRAILS). *Int J Epidemiol* **44**, 76-76n (2015).

80 Liem, E. T. *et al.* Growth during infancy and childhood, and adiposity at age 16 years: ages 2 to 7 years are pivotal. *J Pediatr* **162**, 287-292 e282 (2013).

81 Marchini, J., Howie, B., Myers, S., McVean, G. & Donnelly, P. A new multipoint method for genome-wide association studies by imputation of genotypes. *Nat Genet* **39**, 906-913 (2007).

82 Newnham, J. P., Sharon, S. F., Michael, C. A., Stanley, F. J. & Landau, L. I. [Effects of frequent ultrasound during pregnancy: a randomised controlled trial]
